# In utero human cytomegalovirus infection expands NK-like Fc**γ**RIII^+^CD8^+^ T cells that mediate Fc antibody functions

**DOI:** 10.1172/JCI181342

**Published:** 2024-11-12

**Authors:** Eleanor C. Semmes, Danielle R. Nettere, Ashley N. Nelson, Jillian H. Hurst, Derek W. Cain, Trevor D. Burt, Joanne Kurtzberg, R. Keith Reeves, Carolyn B. Coyne, Genevieve G. Fouda, Justin Pollara, Sallie R. Permar, Kyle M. Walsh

**Affiliations:** 1 Boston Children’s Hospital/Boston Medical Center, Boston, Massachusetts, USA.; 2 Medical Scientist Training Program, and; 3 Duke Human Vaccine Institute, Duke University, Durham, North Carolina, USA.; 4 Department of Surgery, Duke University School of Medicine, Durham, North Carolina, USA.; 5 Children’s Health and Discovery Initiative,; 6 Division of Infectious Diseases, and; 7 Division of Neonatology, Department of Pediatrics, Duke University, Durham, North Carolina, USA.; 8 Carolinas Cord Blood Bank, Marcus Center for Cellular Cures, Durham, North Carolina, USA.; 9 Center for Human Systems Immunology, and; 10 Department of Integrative Immunobiology, Duke University, Durham, North Carolina, USA.; 11 Department of Pediatrics, Weill Cornell Medicine, New York City, New York, USA.; 12 Department of Neurosurgery, Duke University, Durham, North Carolina, USA.

**Keywords:** Immunology, Infectious disease, Immunoglobulins, NK cells, T cells

## Abstract

Human cytomegalovirus (HCMV) profoundly impacts host T and NK cells across the lifespan, yet how this common congenital infection modulates developing fetal immune cell compartments remains underexplored. Using cord blood from neonates with and without congenital HCMV (cCMV) infection, we identify an expansion of Fcγ receptor III–expressing (FcγRIII-expressing) CD8^+^ T cells following HCMV exposure in utero. Most FcγRIII^+^CD8^+^ T cells express the canonical αβ T cell receptor (TCR), but a proportion express noncanonical γδ TCR. FcγRIII^+^CD8^+^ T cells are highly differentiated and have increased expression of NK cell markers and cytolytic molecules. Transcriptional analysis reveals FcγRIII^+^CD8^+^ T cells upregulate T-bet and downregulate BCL11B, known transcription factors that govern T/NK cell fate. We show that FcγRIII^+^CD8^+^ T cells mediate antibody-dependent IFN-γ production and degranulation against IgG-opsonized target cells, similar to NK cell antibody-dependent cellular cytotoxicity (ADCC). FcγRIII^+^CD8^+^ T cell Fc effector functions were further enhanced by IL-15, as has been observed in neonatal NK cells. Our study reveals that FcγRIII^+^CD8^+^ T cells elicited in utero by HCMV infection can execute Fc-mediated effector functions bridging cellular and humoral immunity and may be a promising target for antibody-based therapeutics and vaccination in early life.

## Abstract 2

In a cohort of maternal-fetal dyads, we reveal that human cytomegalovirus infection expands FcγRIII-expressing CD8+ T cells in utero that can mediate NK-like ADCC functions.

## Graphical abstract

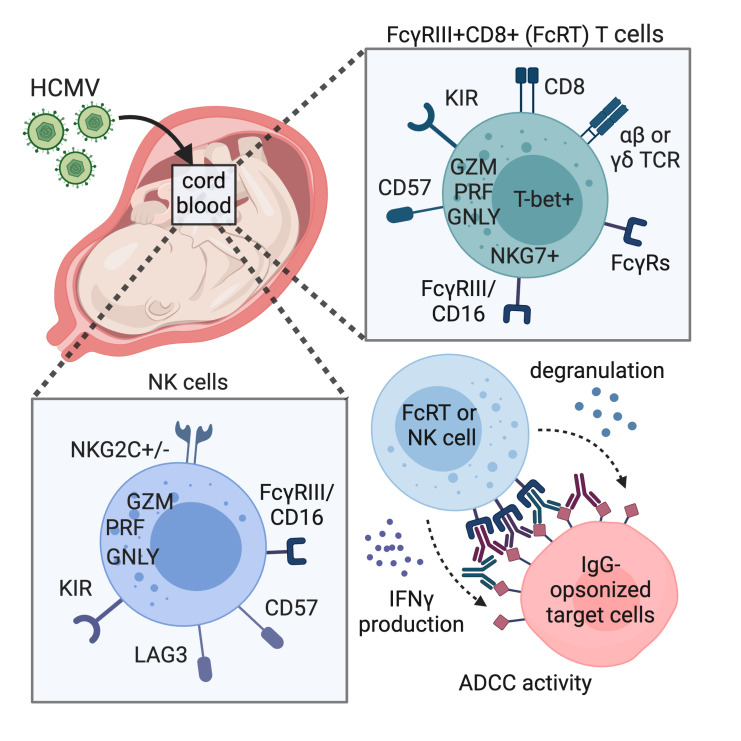

## Introduction

Human cytomegalovirus (HCMV) is a ubiquitous β-herpesvirus that has coevolved with humans and is an important member of the human virome, a dynamic network of commensal and pathogenic viruses (1, 2). Most individuals are latently infected with HCMV (3), and few human pathogens are known to exert such a profound imprint on host immunity across the lifespan (4, 5). While primary infection, latency reactivation, and reinfection are often asymptomatic in healthy children and adults, HCMV can cause severe disease in immunocompromised populations including fetuses, transplant recipients, and persons living with HIV/AIDS. HCMV is the most common congenital infection worldwide and can cause devastating neurologic disease, yet most infants born with HCMV are asymptomatic (6). Intriguingly, while HCMV is a danger to prenatal populations, emerging evidence suggests HCMV may enhance heterologous immunity to other pathogens and vaccines in young, healthy individuals (2, 5, 7).

HCMV infection shapes global immune cell profiles, not just HCMV-specific cells, creating long-lasting shifts in NK and T cell compartments and expanding effector populations bridging innate and adaptive immunity (4, 8). “Memory-like” or “adaptive” NK cells generated by interactions between the HCMV peptide UL40 and NKG2 killer lectin-like (KLR) receptors are persistently expanded in HCMV seropositive individuals and can mediate enhanced antiviral responses upon restimulation (9, 10). HCMV seropositivity has also been associated with the activation and terminal differentiation of bystander non-HCMV specific CD8^+^ T cells (11, 12). Additionally, γδ T cells and canonical CD8^+^ T cells expressing NK cell receptors such as Fcγ receptor III (FcγRIII), also known as CD16), NKG2C, and killer-like immunoglobulin receptors (KIRs) and demonstrating hybrid T-NK cell functions have also been observed in adults with chronic HCMV infection (13–15).

Despite HCMV’s well-known impacts on the adult immune system, our understanding of how HCMV modulates NK and T cells in early life remains limited. Fetal HCMV-specific T cells and γδ T cell subsets can expand following infection (16–20), yet the global impact of HCMV on developing T and NK cells has been underexplored. Vaaben et al. recently reported that fetal NK cells in congenital HCMV (cCMV) infection highly express markers of maturation, activation, and cytotoxicity (21), though the functional capacity of these NK cells is unclear (21). The fetal and neonatal immune landscape is fundamentally distinct from the adult immune system, as it is biased toward immunotolerance and innate immune responses (22, 23), leading us to question how HCMV exposure in utero influences developing T and NK cells.

In this study, we investigated how HCMV impacts fetal T and NK cell populations using banked cord blood from US donors with and without cCMV infection. We characterized cord blood T and NK cells using high-dimensional flow cytometry, machine learning immune cell clustering, transcriptome profiling, and functional assays, identifying a striking expansion of CD8^+^ T cells expressing the NK cell–associated marker FcγRIII in cCMV infection. Fcγ receptor III^+^ (FcγRIII^+^) CD8^+^ T cells were a heterogenous population, mostly expressing αβ T cell receptor (TCR), but some expressing γδ TCR, with an NK-like transcriptional profile and the capacity to mediate Fc antibody effector functions. Our findings suggest that fetal CD8^+^ T cells can be stimulated to differentiate into NK-like T cells that mediate antibody-dependent cellular cytotoxicity (ADCC), an Fc effector function traditionally associated with NK cells. FcγRIII^+^CD8^+^ T cells may have translational potential as an effector cell population linking cellular and humoral immunity that could be harnessed by antibody-based therapeutics or vaccines in early life.

## Results

### Cord blood donor immunophenotyping highlights distinct immune landscape in cCMV-infected versus uninfected neonates.

**Figure 1:**
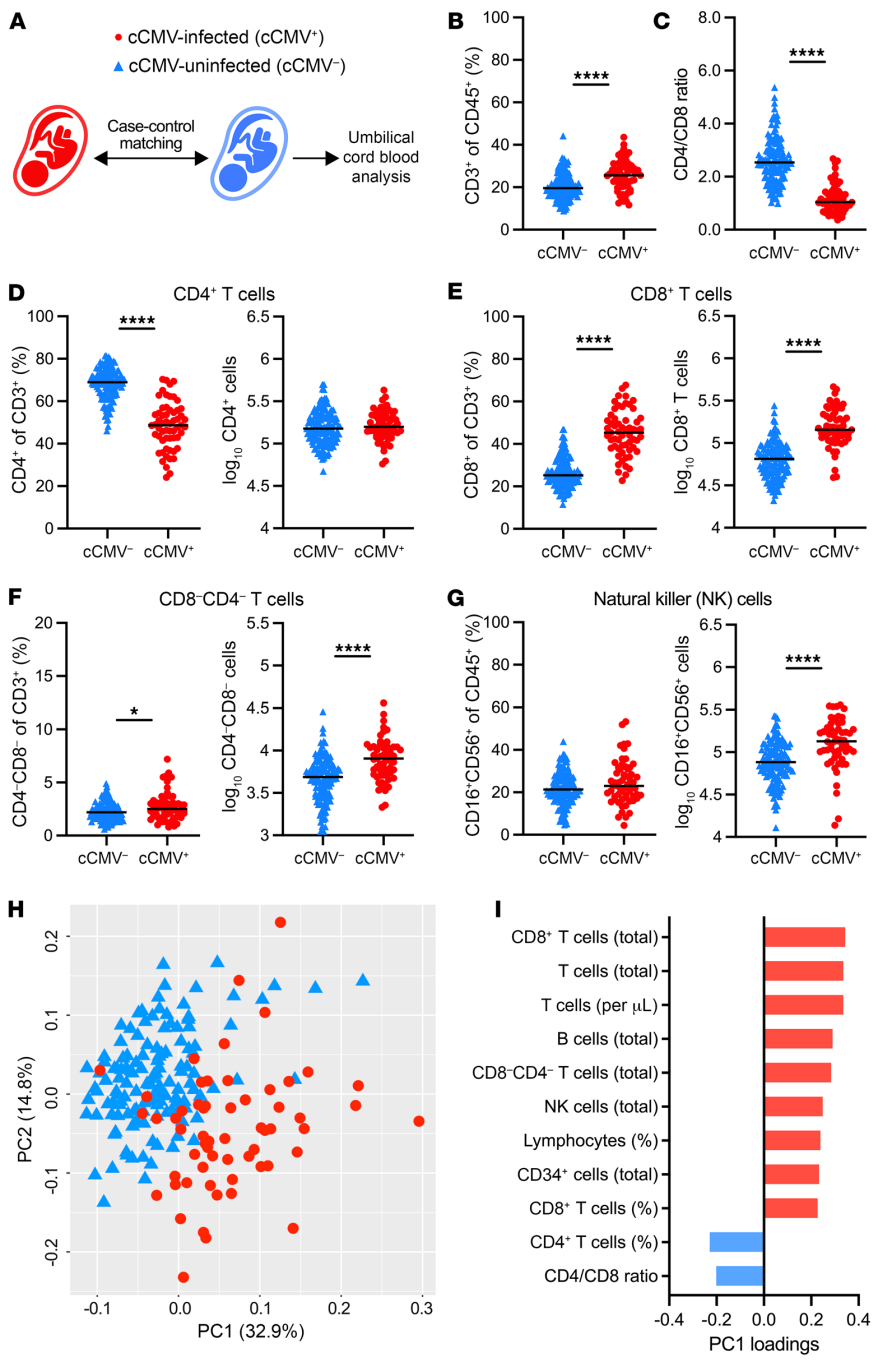
Cord blood donor phenotyping reveals distinct immune landscape in cCMV-infected versus uninfected neonates. Flow cytometry phenotyping of umbilical cord blood from cCMV-infected (cCMV^+^, red circles, *n* = 59) and cCMV-uninfected (cCMV^–^, blue triangles, *n* = 135) neonates was performed by the CCBB at the time of donation. (**A**) Case-control study overview. (**B**–**G**) Frequencies and total immune cell counts (per cord blood collection unit) from CCBB cord blood phenotyping. (**H** and **I**) PCA of 18 immune cell parameters from CCBB phenotyping. (**H**) Scatterplot of PC1 and PC2. (**I**) Immune cell parameter loading variables ordered by magnitude of contribution to PC1 (positive loading variables shown in red due to clustering with cCMV^+^ samples, negative loading variables shown in blue due to clustering with cCMV^–^ samples). FDR-corrected *P* values for Mann-Whitney *U* test. *P < 0.05; ****P < 0.0001.

In this study, we analyzed samples from the US Carolinas Cord Blood Bank (CCBB). In the CCBB donor database, we identified cases of cCMV infection based on positive screening for cord blood HCMV DNAemia (Supplemental Figure 1; supplemental material available online with this article; https://doi.org/10.1172/JCI181342DS1). Using infant sex, race/ethnicity, maternal age, and delivery year as matching variables, HCMV-positive neonates were matched to 2 HCMV-negative donors (Figure 1A). Demographic and clinical characteristics were similar between cCMV-infected (cCMV^+^, *n* = 59) and uninfected (cCMV^–^, *n* = 135) donors (Supplemental Table 1) with no significant differences between groups after correcting for multiple comparisons.

First, we compared cord blood donor immunophenotyping from cCMV^+^ and cCMV^–^ samples. There was a higher proportion of T cells and an inverted ratio of CD4^+^/CD8^+^ T cells, driven by an expansion of CD8^+^ T cells, in cCMV^+^ neonates (Figure 1, B–E). To a lesser degree, CD4^–^CD8^–^ “double negative” T cells, likely representing γδ T cells, (Figure 1F) and CD16^+^CD56^+^ lymphocytes, likely mostly NK cells (Figure 1G), were also expanded in cCMV infection. Next, we used principal components analysis (PCA) to visualize the cord blood immunophenotyping data by cCMV status. PC1 and PC2 accounted for approximately 50% of the variance between donors (Figure 1H). Cord blood immunophenotypes from cCMV^+^ and cCMV^–^ neonates clustered distinctly, with increased CD8^+^ T cells as the top parameter associated with cCMV infection (Figure 1, H and I). PCA visualization by infant sex, race/ethnicity, and delivery mode showed no evidence that these characteristics were underlying differences between groups (Supplemental Figure 2). To explore how HCMV exposure in utero influences developing immune cell compartments, we performed multiparameter flow cytometry and transcriptional profiling of NK and T cells in a subset of cord blood samples from cCMV^+^ (*n* = 21) and cCMV^–^ (*n* = 20) neonates (Supplemental Figures 3 and 4).

### CD56^neg^ FcγRIII/CD16^+^ and NKG2C^+^ NK cells expand in cord blood from cCMV-infected neonates.

**Figure 2:**
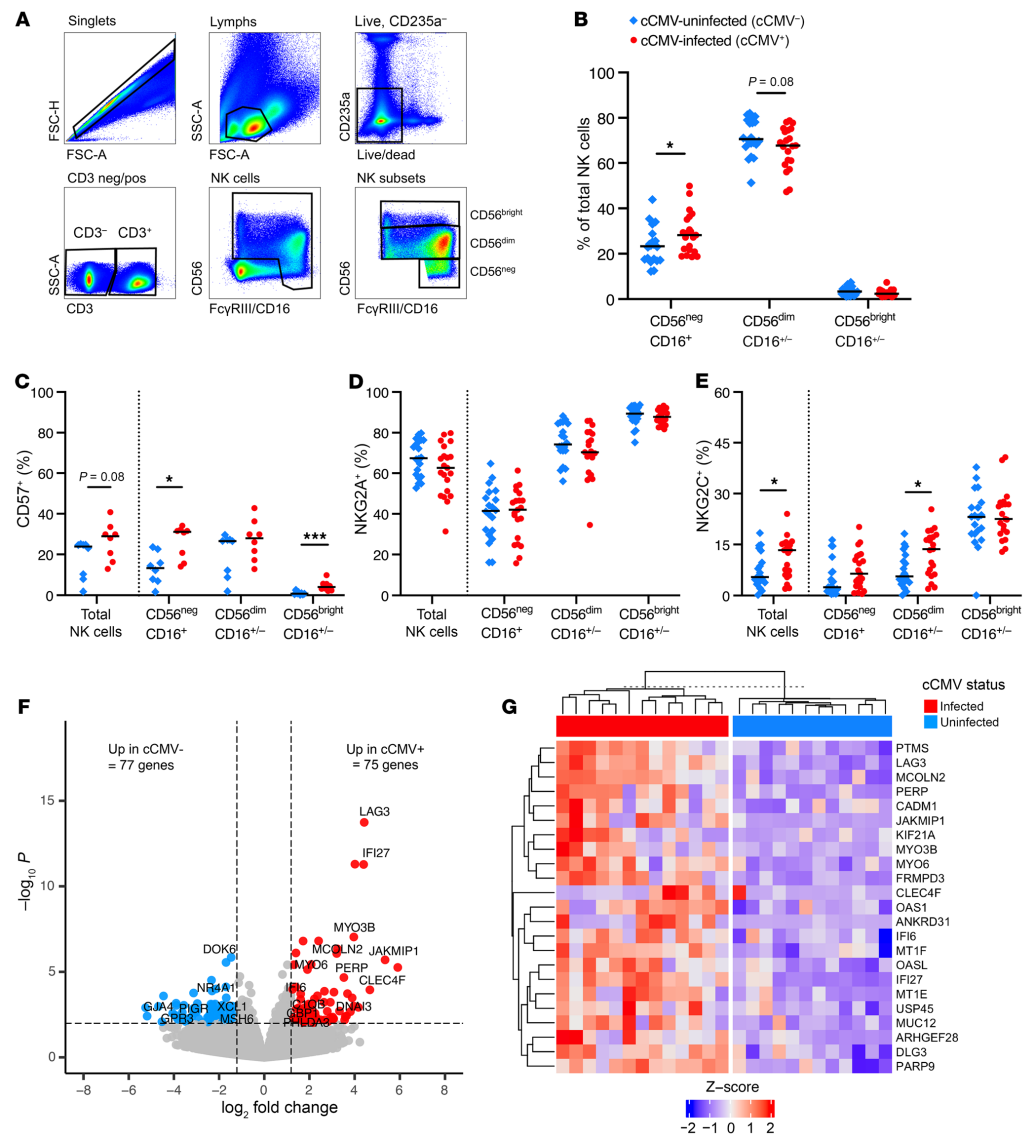
CD56^neg^FcγRIII/CD16^+^ and NKG2C^+^ NK cells expand in cord blood from cCMV-infected neonates. NK cell immunophenotypes and transcriptional profiles were compared in cord blood from cCMV-infected (cCMV^+^, red circles) versus cCMV-uninfected (cCMV^–^, blue diamonds) neonates. (**A**) NK cell gating strategy. (**B**) Frequencies of NK cell subsets in cCMV^+^ (*n* = 21) versus cCMV^–^ (*n* = 20) neonates. (**C**) Frequency of total NK cells and NK cell subsets expressing CD57 in cCMV^+^ (*n* = 8) versus cCMV^–^ (*n* = 8) neonates. (**D** and **E**) Frequency of total NK cells and NK cell subsets expressing (**D**) NKG2A and (**E**) NKG2C in cCMV^+^ (*n* = 21) versus cCMV^–^ (*n* = 20) neonates. (**F** and **G**) RNA-Seq analysis of FAC-sorted NK cells from cCMV^+^ (*n* = 13) and cCMV^–^ (*n* = 12) neonates. (**F**) Volcano plot of differentially expressed genes (*P* < 0.01, log_2_foldchange +/– 1.2). Red circles indicate genes enriched in cCMV^+^, blue circles indicate genes enriched in cCMV^–^. (**G**) Heatmap of top 23 enriched genes (FDR *P* < 0.1, log*2*foldchange > 1.2). *z* score shows gene expression based on rlog-transformed data. FDR-corrected *P* values for Mann-Whitney *U* test. *P < 0.05, ***P < 0.001.

Total NK cells and 3 major NK cell subsets including CD56^neg^CD16^+^, CD56^bright^CD16^+/–^, and CD56^dim^CD16^+/–^ NK cells (21, 24) were compared (Figure 2, A and B). In cCMV infection, CD56^neg^CD16^+^ NK cells were significantly expanded (Figure 2B) and several NK cell subsets had higher expression of CD57 (Figure 2C), a marker of activation and differentiation. NKG2C, but not NKG2A, was also more frequently expressed on NK cells from cCMV^+^ versus cCMV^–^ neonates (Figure 2, D and E).

Next, we compared the transcriptome of FAC-sorted NK cells from cCMV^+^ (*n* = 13) versus cCMV^–^ (*n* = 12) neonates. Differential gene expression analysis identified 75 upregulated and 77 downregulated genes (Figure 2, F and G), though only 29 upregulated and 12 downregulated genes remained significant after FDR correction (*P*_FDR_ < 0.1). Expression of LAG3, a checkpoint inhibitor induced by type I IFN that is highly expressed by ADCC-mediating CD56^neg^CD16^+^ NK cells (24), was 4-fold higher in NK cells from cCMV^+^ versus cCMV^–^ neonates (*P*_FDR_ = 2.53 × 10^–10^). JAKMIP1, a marker of adaptive NK cells in chronic HCMV infection (25), was also elevated 5-fold (*P*_FDR_ = 2.53 × 10^–3^). Enriched gene ontology (GO) pathways in upregulated genes included innate immune response (*P_adj_* = 1.0 × 10^–4^), defense to virus (*P_adj_* = 2.5 × 10^–4^), and type I interferon signaling (*P_adj_* = 6.7 × 10^–4^). Together, these data demonstrate that NK cells with an antiviral transcriptional program expand in cCMV infection but limited subsets express NKG2C, the characteristic marker of “memory-like” NK cells that expand in adult infection (8).

### Minor phenotypic and transcriptional changes in CD4^+^ T cells following cCMV infection.

The proportion of total CD4^+^ T cells was lower in cCMV^+^ versus cCMV^–^ infants, yet there were no differences in naive, central memory (Tcm), effector memory (Tem), terminally differentiated effector memory T cells reexpressing CD45RA^+^ (Temra), or Treg subsets (Supplemental Figure 5, A and B). CD4^+^ T cells expressing activation (HLA-DR), differentiation (CD57), and antigen stimulation (PD-1) markers were increased in cCMV infection, but there was overall low abundance of these CD4^+^ subsets (Supplemental Figure 5C). Differential gene expression analysis identified 180 upregulated and 368 downregulated genes in CD4^+^ T cells from cCMV^+^ (*n* = 11) versus cCMV^–^ (*n* = 12) neonates (Supplemental Figure 5D), yet only 25 upregulated and 37 downregulated genes were significant after FDR correction (*P*_FDR_ < 0.1). Expression of CCL5 (*P*_FDR_ = 1.10 × 10^–8^), NK gene 7 (NKG7, *P*_FDR_ = 1.97 × 10^–6^), which helps traffic cytotoxic vesicles to the immunological synapse, and granzyme H (*P*_FDR_ = 5.8 × 10^–6^) were upregulated 5- to 7-fold (Supplemental Figure 5, D and E). Taken together, these data suggest that HCMV activates a minor subset of fetal CD4^+^ T cells, particularly those that may recruit cytotoxic cells or direct cytotoxic activity.

### CD8^+^ T cells upregulate cytolytic and NK cell–associated genes in cCMV infection.

**Figure 3:**
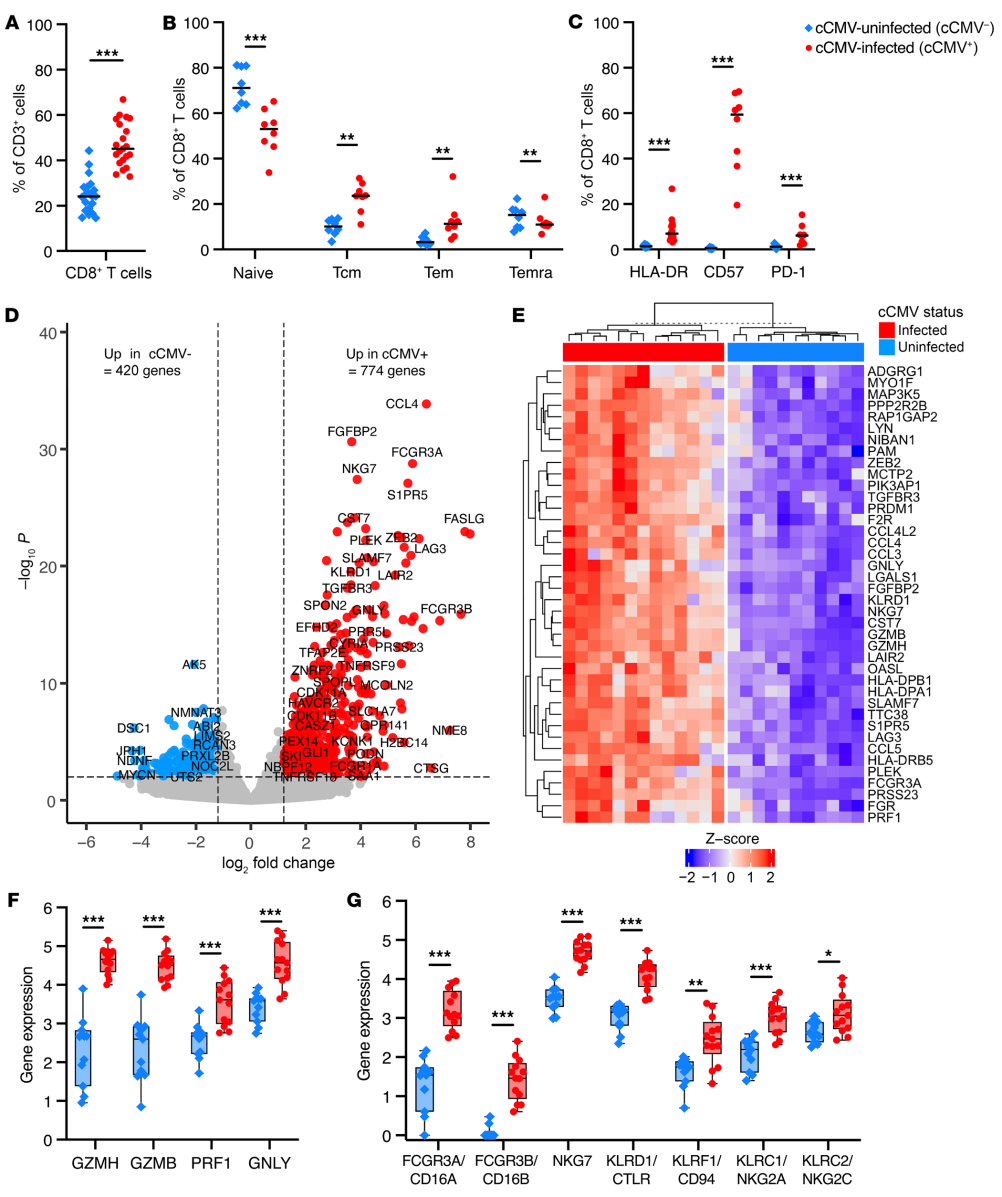
CD8^+^ T cells upregulate cytotoxicity and NK cell genes in cord blood from cCMV-infected neonates. (**A** and **B**) CD8^+^ T cell immunophenotypes were compared in cord blood from cCMV-infected (cCMV^+^, red circles, *n* = 21) versus cCMV-uninfected (cCMV^–^, blue diamonds, *n* = 20) neonates. (**A** and **B**) Frequency of total, naive, Tcm, Tem, and Temra CD8^+^ T cells. (**C**) Frequency of CD8^+^ T cells expressing HLA-DR, CD57, and PD-1 in cCMV^+^ (*n* = 8) versus cCMV^–^ (*n* = 8) neonates. (**D**–**G**) RNA-Seq analysis of FAC-sorted total CD8^+^ T cells from cCMV^+^ (*n* = 13) and cCMV^–^ (*n* = 11) neonates. (**D**) Volcano plot demonstrating differentially expressed genes (*P* < 0.01, log_2_foldchange +/– 1.2). Red circles indicate genes enriched in cCMV^+^, blue circles indicate genes enriched in cCMV^–^, and gray circles indicate genes whose expression did not differ significantly. (**E**) Heatmap of top 40 enriched genes (FDR *P* < 0.1, log_2_foldchange > 3.0). (**F** and **G**) Expression of genes encoding (**F**) cytolytic molecules and (**G**) NK-associated cell markers. *z* score shows gene expression based on rlog-transformed data. FDR-corrected *P* values for Mann-Whitney *U* test. *P < 0.05; **P < 0.01; ***P < 0.001.

Total CD8^+^ T cells and Tcm/Tem subsets were increased in cCMV^+^ versus cCMV^–^ neonates (Figure 3, A and B). CD57 was expressed on the majority of CD8^+^ T cells from cCMV^+^ infants (median = 59%) versus less than 1% of cCMV^–^ infants (Figure 3C). Differential gene expression analysis identified 774 upregulated and 420 downregulated genes in CD8^+^ T cells from cCMV^+^ (*n* = 13) versus cCMV^–^ (*n* = 11) groups (Figure 3, C and D), which all remained statistically significant after FDR correction (*P*_FDR_ < 0.1). Chemokines CCL3 (*P*_FDR_ = 1.1 × 10^–18^), CCL4 (*P*_FDR_ = 2.1 × 10^–30^), and CCL5 (*P*_FDR_ = 1.7 × 10^–20^) were upregulated (Figure 3, D and E). Expression of cytolytic molecules granzyme H (*P*_FDR_ = 1.6 × 10^–11^), granzyme B (*P*_FDR_ = 3.8 × 10^–18^), perforin (PRF1) (*P*_FDR_ = 9.2 × 10^–12^), granulysin (GNLY) (*P*_FDR_ = 2.7 × 10^–14^), and NKG7 (*P*_FDR_ = 1.5 × 10^–24^) were increased 3- to 5-fold in cCMV infection (Figure 3, D–F). Expression of genes encoding FcγRIIIa/CD16A (*P*_FDR_ = 8.8 × 10^–26^), FcγRIIIb/CD16B (*P*_FDR_ = 1.7 × 10^–13^), and KLRs were also increased (Figure 3, E and G). Gene set enrichment analysis revealed that the top induced pathways in CD8^+^ T cells from cCMV^+^ infants included NK cell–mediated immunity (*P*_adj_ = 1.3 × 10^–3^), NK cell–mediated cytotoxicity (*P*_adj_ = 1.3 × 10^–3^), and regulation of NK cell immunity (*P*_adj_ = 2.9 × 10^–3^) (Supplemental Table 2). Together, these data indicate that CD8^+^ T cells exposed to HCMV in utero have high cytotoxic potential and upregulate NK cell–associated genes that may contribute to antiviral functions.

### CD8^+^ T cells expressing FcγRIII and NKG2A/C expand in cCMV infection.

**Figure 4:**
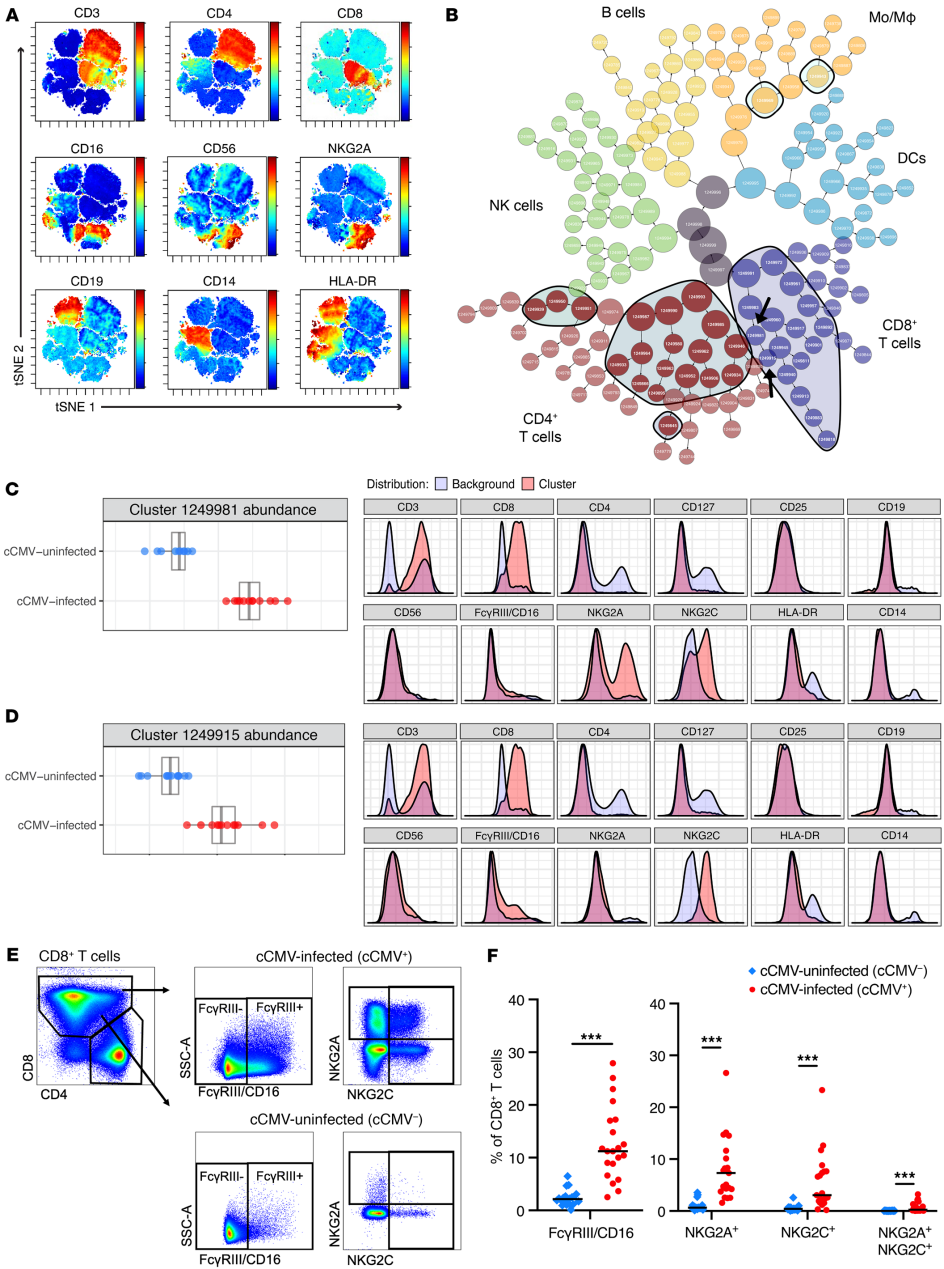
CD8^+^ T cells expressing NK cell receptors FcγRIII and NKG2A/C expand in cord blood from cCMV-infected neonates. (**A**–**D**) CITRUS analysis of flow cytometry data was used to identify immune cell populations with differing abundance in cord blood from cCMV-infected (*n* = 13) versus cCMV-uninfected (*n* = 12) neonates. (**A**) t-SNE-CUDA dimensionality reduction of flow cytometry data prior to CITRUS. (**B**) CITRUS cluster map with black outlines and shaded areas indicating clusters that differed significantly (FDR *P* < 0.01) between cCMV^+^ and cCMV^–^ groups. Clusters colored by CD8^+^ T cells (purple), CD4^+^ T cells (red), NK cells (green), B cells (yellow), monocytes/macrophages (MO/Mϕ; orange) and dendritic cells (DCs; blue) based on marker expression (General Lineage CITRUS in the Supplemental Material). (**C** and **D**) Select clusters (black arrows in panel **B**) of CD8^+^ T cells expressing NK cell markers. Dot plots indicate cluster abundance in cCMV^+^ (red circles) versus cCMV^–^ (blue circles) neonates. Histograms indicate fluorescent marker expression of select cluster (pink) relative to background (blue). (**E**) Gating strategy to identify CD8^+^ T cells expressing NK cell markers. (**F**) Frequency of FcγRIII and NKG2A/C expression on CD8^+^ T cells from cCMV^+^ (red circles, *n* = 21) versus cCMV^–^ (blue diamonds, *n* = 20) neonates. FDR-corrected *P* values for Mann-Whitney U test. ***P < 0.001.

To define the CD8^+^ T cell populations underlying these transcriptional changes, we used CITRUS (cluster identification, characterization, and regression), a machine learning algorithm that employs unsupervised hierarchical clustering of flow cytometry data to identify immune cell populations that differ between groups rather than traditional Boolean gating (26). We first used t-SNE-CUDA, a CUDA (compute unified device architecture) optimized method of T distributed stochastic neighborhood embedding (t-SNE) for data dimensionality reduction (27), to visualize our data (Figure 4A) and selected fluorescent channels for CITRUS analysis. CD3, CD4, CD8, CD127, CD25, CD19, CD56, FcγRIII/CD16, NKG2A, NKG2C, HLA-DR, and CD14 marker expression on 1,250,000 cells (50,000 cells/sample) was used to generate the CITRUS cluster map (Figure 4B and Supplemental Figure 6). CITRUS identified 38 immune cell clusters that differed significantly (*P*_FDR_ < 0.01) between cCMV^+^ and cCMV^–^ groups, including clusters of activated CD8^+^ and CD4^+^ T cells that we previously identified with manual gating (General Lineage CITRUS in the Supplemental Material, Figure 4B). Two clusters in the CD8^+^ T cell “branch,” one coexpressing NKG2A and NKG2C (Figure 4C) and the other coexpressing FcγRIII and NKG2C (Figure 4D), were also more abundant in cCMV infection. These populations clustered distinctly from the NK cell “branch” and expressed the T cell marker CD3 (Figure 4, B–D). Using manual gating to confirm our CITRUS analysis (Figure 4E), we found that CD8^+^ T cells expressing FcγRIII were significantly enriched in cord blood from cCMV^+^ neonates (Figure 4F). Frequency of CD8^+^ T cells expressing NKG2A/NKG2C was also higher in cCMV infection and nearly absent (median <1%) in cCMV^–^ infants (Figure 4F). Together, these data demonstrate that CD8^+^ T cells expressing the NK cell–associated receptors FcγRIII and NKG2A/C expand in utero following HCMV infection.

### FcγRIII^+^CD8^+^ T cells include canonical αβ and nontraditional γδ T cell populations.

**Figure 5:**
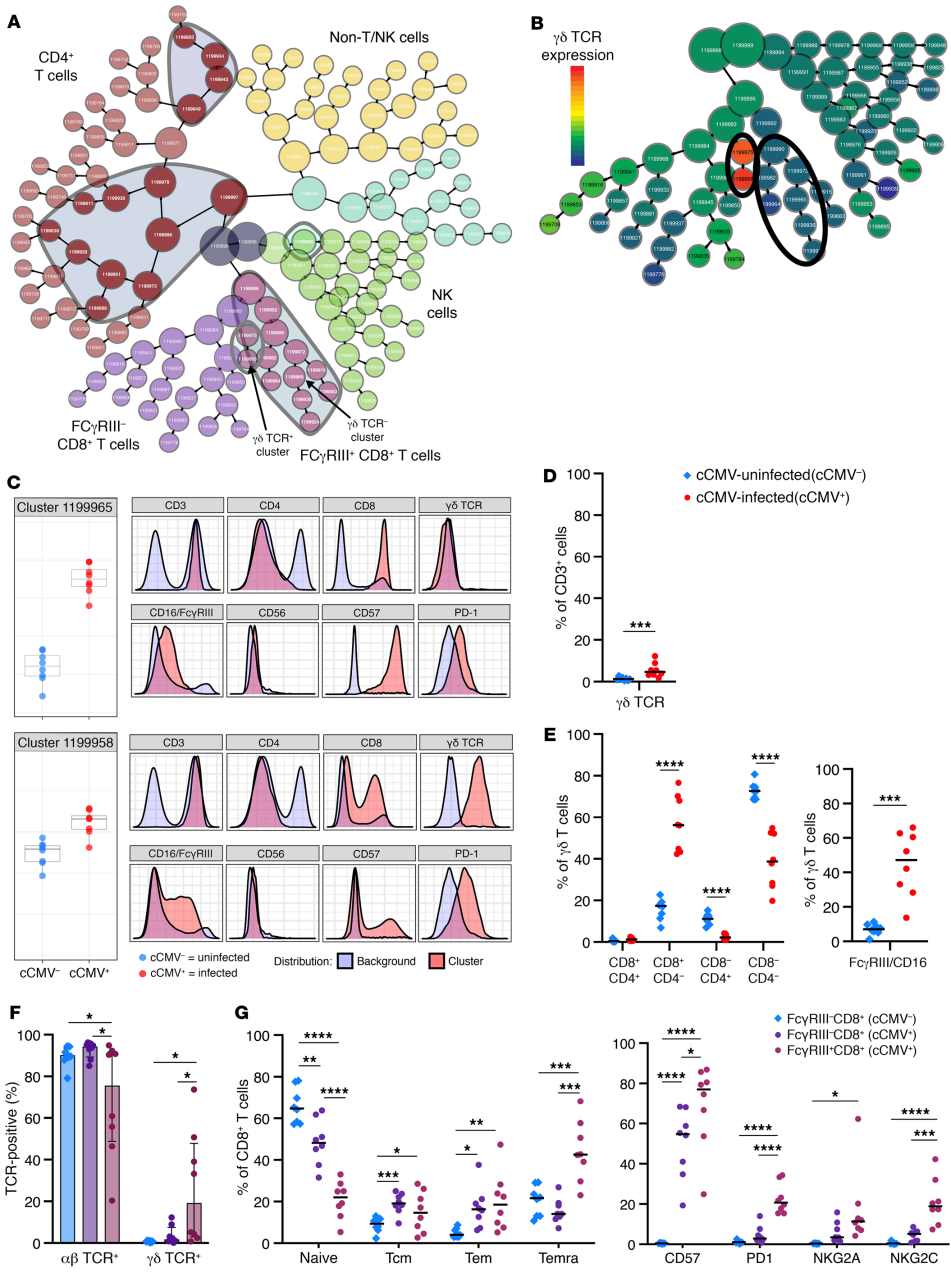
FcγRIII^+^ CD8^+^ T cells include canonical αβ and unconventional γδ T cell populations. (**A**–**G**) Immunophenotypes in cord blood from cCMV-infected (*n* = 8, circles) versus uninfected (*n* = 8, diamonds) neonates. (**A** and **B**) CITRUS cluster map with gray outlines and shaded areas indicating immune cell clusters that differed significantly (FDR *P* < 0.01) between groups. Clusters colored by CD8^+^ T cells (purple, plum shade for increased FcγRIII expression), CD4^+^ T cells (red), NK cells (green), and non-T/NK cells (yellow, aqua) based on marker expression (NK T CITRUS in the Supplemental Material). (**B**) Expression of γδ TCR with black outlines showing FcγRIII^+^ CD8^+^ T cell clusters. (**C**) FcγRIII^+^ CD8^+^ T cell clusters (arrows in panel **A**). Dot plots indicate cluster abundance in cord blood from cCMV^+^ (red) versus cCMV^–^ (blue) neonates. Histograms indicate fluorescent marker expression of select cluster (pink) relative to background (blue). (**D** and **E**) γδ T cells in cord blood from cCMV^+^ (red) versus cCMV^–^ (blue) neonates. (**D**) Frequency of total γδ T cells and (**E**) γδ T cells expressing CD8/CD4 and FcγRIII. (**F**) Expression of αβ and γδ TCR and (**G**) differentiation/functional markers (**F**) on FcγRIII^–^ (cCMV^+^ purple; cCMV^–^ blue) and FcγRIII^+^ (plum) CD8^+^ T cells. FDR-corrected *P* values for Mann-Whitney *U* test (**D** and **E**) and ANOVA followed by Tukey’s post hoc test (**F** and **G**). *P < 0.05; **P < 0.01; ***P < 0.001; ****P < 0.0001.

CD8^+^ T cells expressing NK cell markers have been described in chronic HCMV, EBV, HIV, and hepatitis C virus (HCV) infections in adults (28–32), prompting us to perform additional T and NK cell phenotyping to define these populations in the fetal immune context. CITRUS analysis of CD3, CD4, CD8, CD56, FcγRIII/CD16, γδ TCR, CCR7, CD45RA, PD-1, CD57, NKG2A, and NKG2C marker expression on 1,200,000 cells (75,000 cells/sample) generated a map with distinct “branches” of T and NK cell clusters and identified 28 clusters that differed significantly (*P*_FDR_ < 0.01) between cCMV^+^ (*n* = 8) and cCMV^–^ (*n* = 8) groups (Figure 5A and Supplemental Figure 7). Multiple CD8^+^ T cell clusters expressing FcγRIII were enriched in cCMV infection (Figure 5A). Most FcγRIII^+^CD8^+^ T cell clusters resembled canonical CD8^+^ T cells, whereas 2 clusters expressed γδ TCR (Figure 5, B and C).

Though low abundance overall, γδ T cells were expanded in cCMV^+^ (median = 4.7%) versus cCMV^–^ (median = 1.3%) groups (Figure 5D). While γδ T cells are typically CD8^–^CD4^–^ “double negative,” the proportion of CD8^+^CD4^–^ γδ T cells was particularly increased in cCMV^+^ (median = 56%) versus cCMV^–^ (median = 17%) groups (Figure 5E), though all γδ T cell subsets were expanded. Frequency of FcγRIII expression was higher on γδ T cells from cCMV^+^ (median = 47%) versus cCMV^–^ (median = 7%) groups (Figure 5E). Within the FcγRIII^+^CD8^+^ T cell population, γδ TCR was expressed on a minority of cells (median = 19%) whereas αβ TCR was expressed on a majority of cells (median = 76%), though there was heterogeneity across samples (Figure 5F). Most FcγRIII^+^CD8^+^ T cells were Temra and had increased expression of CD57, PD-1, and NKG2C compared with CD8^+^ T cells lacking FcγRIII (Figure 5G). Overall, these data suggest that HCMV stimulates fetal CD8^+^ T cells, including αβ and γδ T cells, to differentiate and acquire NK cell–associated receptors in utero.

### FcγRIII^+^CD8^+^ T cells in cord blood from cCMV-infected neonates upregulate NK cell genes.

**Figure 6:**
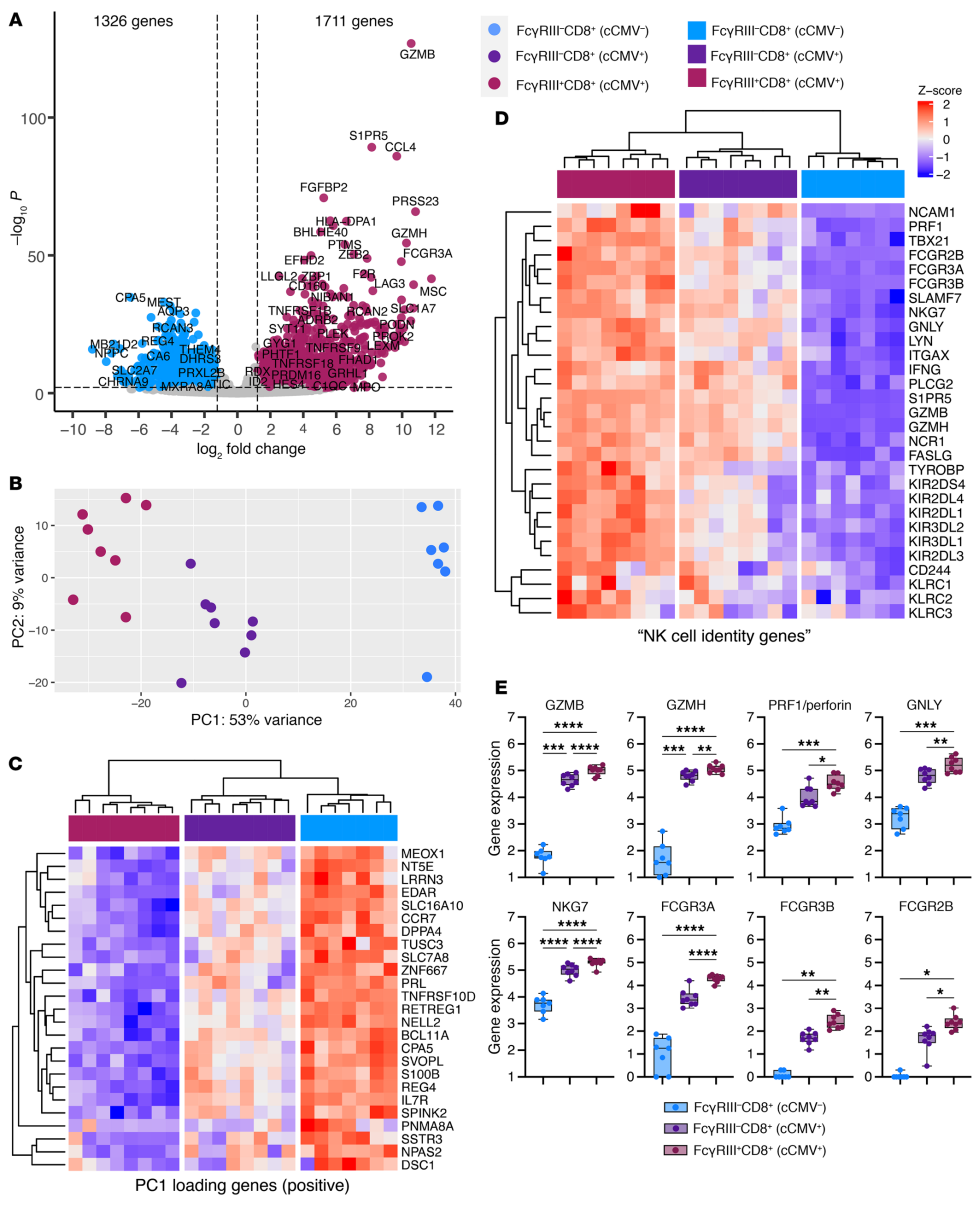
FcγRIII^+^ CD8^+^ T cells in cord blood from cCMV-infected neonates upregulate NK cell identity genes. (**A**–**E**) Transcriptome analysis of FAC-sorted FcγRIII^+^ and FcγRIII^–^ CD8^+^ T cells from cCMV-infected (*n* = 8) and cCMV-uninfected (*n* = 7) neonates. (**A**) Volcano plot of differentially expressed genes in FcγRIII^+^ versus FcγRIII^–^CD8^+^ T cells (*P* < 0.01, log_2_foldchange +/– 1.2). Plum circles indicate genes enriched in FcγRIII^+^CD8^+^ T cells (cCMV^+^ only), blue circles indicate genes enriched in FcγRIII^–^CD8^+^ T cells (cCMV^–^ only). (**B**) PCA of top 500 differentially expressed genes in FcγRIII^+^ versus FcγRIII^–^CD8^+^ T cells. (**C**) Heatmap of top 25 PC1 loading genes (panel **B**). (**D**) Heatmap of NK cell identity genes. (**E**) Cytotoxicity and FcγR gene expression levels. *z* score shows gene expression based on rlog-transformed data. FDR-corrected *P* values for ANOVA followed by Tukey’s post hoc test. *P < 0.05; **P < 0.01; ***P < 0.001; ****P < 0.0001.

Next, we compared the transcriptome of FAC-sorted FcγRIII^+^CD8^+^ T (which we refer to as FcRT cells) and FcγRIII^–^CD8^+^ T cells from cCMV^+^ and cCMV^–^ neonates (Supplemental Figure 3). Cytolytic molecules and chemokines were upregulated in FcγRIII^+^ and FcγRIII^–^CD8^+^ T cells in cCMV infection (Figure 6A and Supplemental Figure 8, A–D). FcγRIII^–^CD8^+^ T cells from cCMV^–^ infants had a distinct transcriptional profile with enriched expression of IL-7R and CCR7, markers of naive T cells (Figure 6, B and C). KIR and KLR expression were upregulated in FcRT cells, as were additional NK cell identity genes including CD244, NCR1/NKp46, NCAM1, and TYROBP (Figure 6D and Supplemental Figure 8, C and D), which were examined based on prior literature (14, 32). FcRT cells had increased expression of genes encoding granzyme B, granzyme H, PRF1, GNLY, and NKG7, indicating high cytolytic potential, and multiple FcγRs that can mediate Fc effector functions like ADCC and antibody-dependent cellular phagocytosis (ADCP) (Figure 6, D and E).

**Figure 7:**
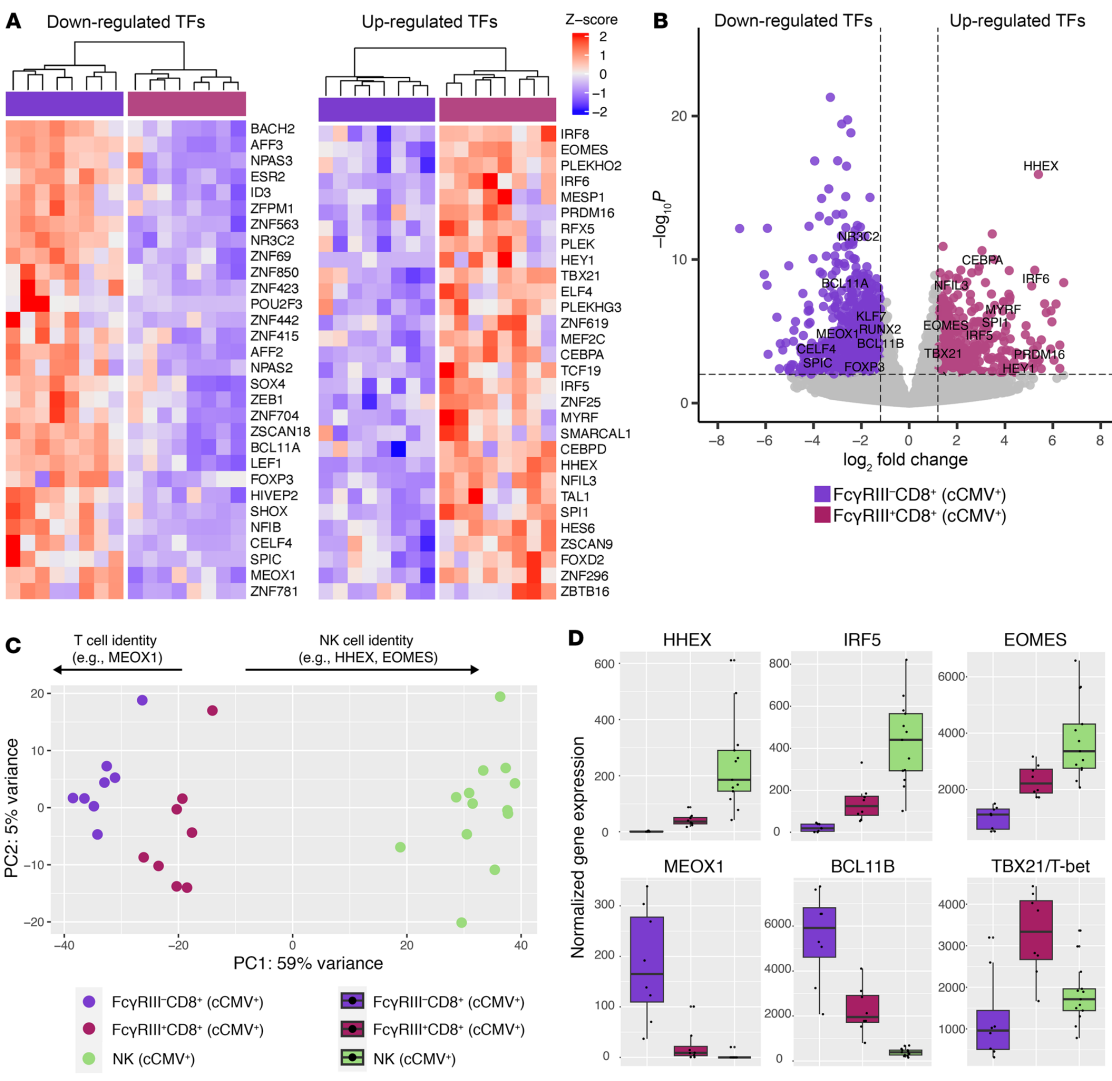
TFs expressed by FcγRIII^+^CD8^+^ T cells suggest shift from T to NK identity. (**A**–**D**) RNA-Seq analysis of TF expression from FAC-sorted FcγRIII^+^CD8^+^ T cells (*n* = 8, plum), FcγRIII^–^CD8^+^ T cells (*n* = 8, dark purple), and NK cells (*n* = 13, green) in cord blood from cCMV-infected neonates. (**A** and **B**) Heatmap and volcano plot showing top downregulated (left) and upregulated (right) TFs (FDR *P* <0.05, log_2_foldchange +/– 1.2) in FcγRIII^+^ versus FcγRIII^–^CD8^+^ T cells. (**B**) Volcano plot of all differentially expressed genes with TFs labeled. (**C**) PCA of top 500 differentially expressed genes in CD8^+^ T and NK cells. (**D**) Boxplots show normalized gene expression levels of TFs. *z* score shows gene expression based on rlog-transformed data.

Next, we compared transcription factor (TF) expression in CD8^+^ T cells and NK cells from cCMV^+^ infants. We found 57 TFs were upregulated and 101 TFs were downregulated in FcRT versus FcγRIII^–^CD8^+^ T cells (Figure 7, A and B). To assess which TFs may be driving CD8^+^ T cells toward an NK-like profile, we performed a PCA of bulk RNA-Seq data from sorted CD8^+^ T cells and NK cells (Figure 7C). We identified TFs HHEX, IRF5, and EOMES as associated with NK cell identity and TFs MEOX1 and BCL11B as associated with T cell identity, whereas the TF T-bet was enriched most in FcRT cells (Figure 7, C and D). Together, these data demonstrate that FcRT cells elicited in utero during HCMV infection acquire an NK-like transcriptional profile, likely governed by shifts in TFs regulating T/NK maturation.

### Cord blood NK and Fcγ0RIII^+^CD8^+^ T cells produce IFN-γ and degranulate against antibody-opsonized target cells.

**Figure 8:**
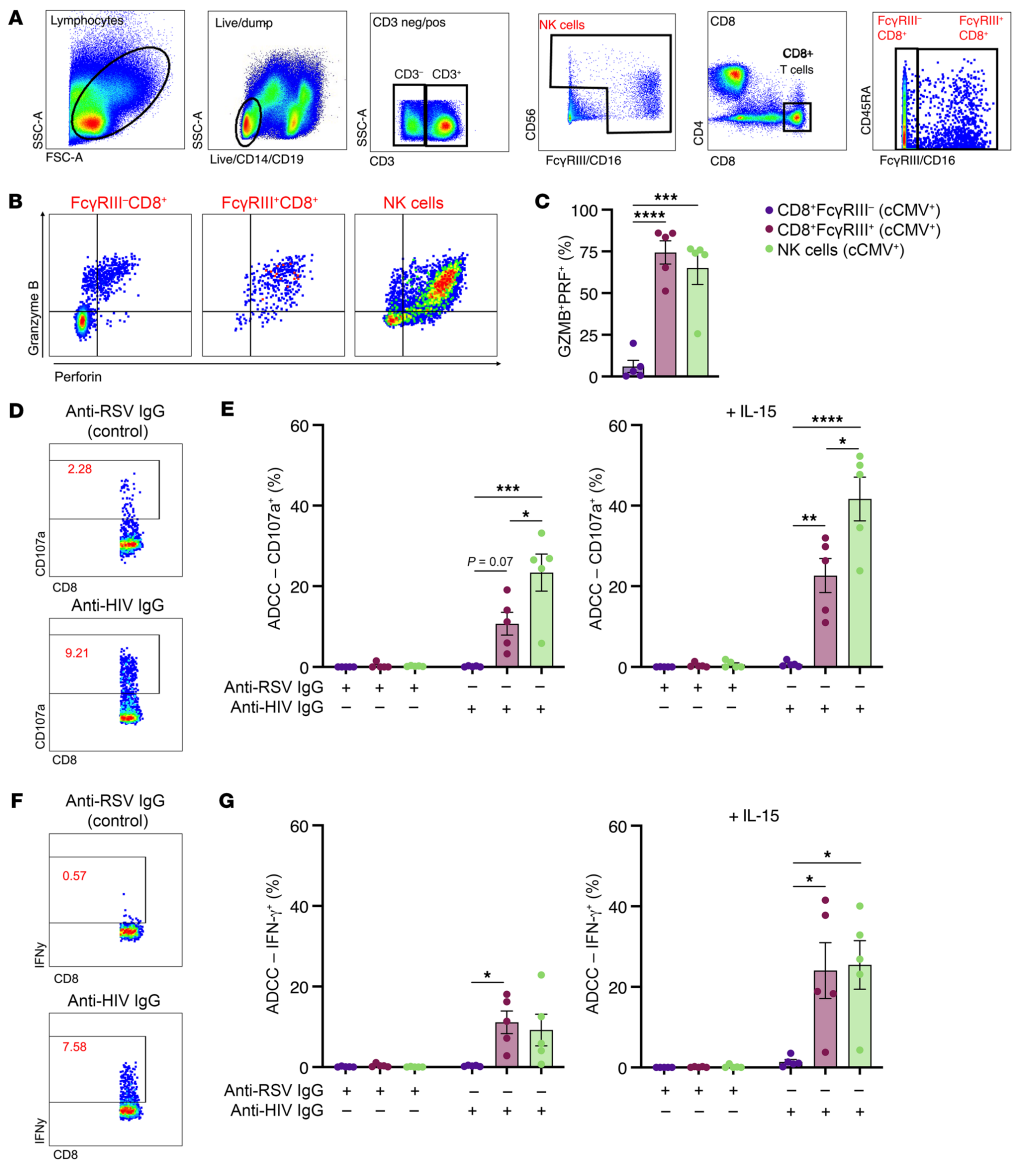
Cord blood FcγRIII^+^ CD8^+^ T cells and NK cells mediate ADCC functions. (**A**–**G**) Degranulation (CD107a positivity) and IFN-γ production following antibody stimulation with anti-RSV IgG (nonspecific antibody) or anti-HIV IgG (target cell specific antibody) were measured as markers of ADCC in cord blood from cCMV-infected (*n* = 5) and cCMV-uninfected (*n* = 5, Supplemental Figure 9) in 2 independent experiments. (**A**) Gating strategy to identify NK cells and CD8^+^ T cells with and without FcγRIII expression. (**B**) Gating strategy for granzyme B and PRF1 expression. (**C**) Percent of population coexpressing granzyme B and PRF1. (**D**–**G**) ADCC activity in FcγRIII^–^CD8^+^ T cells (dark purple), FcγRIII^+^CD8^+^ T cells (plum), and NK cells (light green) from cCMV-infected infants. (**D** and **E**) T cell and NK degranulation (CD107a positivity) following antibody stimulation with and without IL-15 pretreatment. (**F** and **G**) T cell and NK IFN-γ production following antibody stimulation with and without IL-15 pretreatment. FDR-corrected *P* values for ANOVA followed by Tukey’s post hoc test. *P < 0.05; **P < 0.01; ***P < 0.001; ****P < 0.0001.

To assess whether FcRT cells are activated by Fc-IgG binding and mediate Fc effector functions, we measured antibody-dependent IFN-γ production and degranulation on CD8^+^ T and NK cells (Figure 8A). We observed high intracellular expression of PRF1 and granzyme B in FcRT cells with levels similar to autologous NK cells (Figure 8, B and C). To test FcγR function, we employed a validated assay for NK cell ADCC (33) using an HIV model system (all donors were HIV negative, ensuring responses to the antigen were mediated by antibodies and not memory T cell responses). NK cell degranulation and IFN-γ production to antibody stimulation were comparable in cord blood from cCMV^+^ and cCMV^–^ neonates (Supplemental Figure 9, A and B). FcγRIII^–^CD8^+^ T cells from cCMV^+^ and cCMV^–^ infants did not degranulate or produce effector cytokines against HIV antigen-coated cells when coincubated with nonspecific or HIV-specific antibodies (Figure 8, D–G, and Supplemental Figure 9, C and D). In contrast, FcRT cells from cCMV^+^ neonates degranulated, as measured by CD107a expression, and produced IFN-γ in an antigen-specific, antibody-dependent manner (Figure 8, D–G). IL-15 augments ADCC activity in neonatal NK cells (34), so we examined whether FcRT cells were also responsive to IL-15. We found that FcRT cells had increased degranulation and IFN-γ production following IL-15 stimulation, similar to autologous NK cells (Figure 8, E and G, and Supplemental Figure 9, A–D). Taken together, these data demonstrate that cord blood NK and FcRT cells elicited in utero following HCMV infection mediate ADCC functions that are enhanced by effector cytokines.

## Discussion

Congenital infections such as HCMV pose a unique challenge to the developing immune system, which must balance the competing demands of antipathogen defense versus immunotolerance to maternal alloantigens, commensal microbiota, and environmental antigens (22). Thus, fetal and neonatal immune cells favor innate over adaptive responses (23, 35) and antigen-specific responses by developing T and B cells remain limited. While HCMV-specific T cells have been observed in cord blood from cCMV-infected infants, adaptive T cell responses against HCMV are constrained by an immature T cell compartment and functional exhaustion in utero (17–19). Here, we demonstrate that HCMV infection expands a population of cord blood CD8^+^ T cells expressing FcγRIII and capable of Fc effector functions traditionally associated with NK cells. Increased expression of granzyme, PRF1, GNLY, and NKG7 (36, 37) indicates that FcγRIII^+^CD8^+^ T cells (referred to as FcRT cells) are polyfunctional and highly cytotoxic. We show that cord blood FcRT cells and NK cells from cCMV-infected neonates respond to antibody stimulation with degranulation and IFN-γ production, indicating that both populations are poised to mediate ADCC. Our work identifies an alternative pathway by which the developing immune system can overcome the limits to TCR-mediated immunity by engaging CD8^+^ T cells in Fc-mediated immunity. In summary, our study suggests that CD8^+^ T cells and NK cells expressing FcγRIII can bridge cellular and humoral immunity though Fc effector functions in early life.

NK-like CD8^+^ T cells have been described in adults with chronic HIV (32, 38), HCV (31), EBV (30), and HCMV (14, 28, 29) infections. Prior work has shown that FcγRIII-expressing CD8^+^ T cells in this adult context can mediate ADCC (30–32) that can be enhanced by IL-15 (39). These NK-like CD8^+^ T cells do not fit the characteristics of invariant natural killer T cells (iNKT), but rather represent separate heterogenous subpopulations of cytotoxic T cells capable of innate and adaptive responses (40). To our knowledge, our work newly demonstrates that FcγRIII-expressing CD8^+^ T cells with a similar transcriptional profile, cytokine responsiveness, and functionality can be induced in an immature, developing immune system. Given the limited gestational window when these infections occur, our identification of FcyR-expressing CD8^+^ T cells in cord blood challenges the assumption that these cells only develop over months to years following chronic antigenic stimulation (40). That these FcRT cells had upregulated expression of T-bet, EOMES, and HHEX, which regulate the maturation of IL-15–responsive cytotoxic CD8^+^ T cells and NK cells in adults (41–43), suggests that transcriptional reprogramming governed by these TFs may also drive the development of these cells in utero. Furthermore, FcRT cells had downregulation of BCL11B, which regulates the development of CD8^+^ T cells with NK identity in chronic HCMV infection (14, 44–46). Altogether, our findings indicate that this may be a more conserved and fundamental pathway for T cells to take given the ability of developing fetal T cells to acquire these transcriptional changes and functions. That the fetal immune system can rapidly develop NK-like CD8^+^ T cells in response to an infectious stimulus suggests that neither chronic infection nor a fully developed adult immune system is required to generate these cells. Our work reveals that FcRT cells may be a previously unappreciated effector cell population that could contribute to host defense in early life by acquiring Fc antibody effector functions.

Our finding that a subset of FcRT cells expressed γδ TCR expands upon our current understanding of γδ T cells in HCMV and other infections. Prior studies reported that fetal γδ T cells expressing NK receptors expand in cCMV infection and have cytotoxic functions, but did not examine CD8 expression nor FcγR-mediated functions (20). FcγRIII-expressing γδ T cells capable of ADCC have been observed in adults with chronic antigenic exposures (15, 47–49), though the ontogeny of these cells remains unclear. Moreover, FcγRIII-expressing γδ T cells with upregulation of T-bet and robust ADCC responses have been observed in children with malaria (47) and latent tuberculosis (TB) (50). Our study reveals that similar populations can be generated by the fetal immune system. Since γδ T cells are present in early gestation before antigen-specific T cells develop, γδ T cells bridging humoral and cellular immunity are an attractive target to protect the maternal-fetal dyad.

There were minor shifts in the NK cell compartment compared with the CD8^+^ T cell compartment following cCMV infection. NKG2C, a marker for the “memory-like” NK cells associated with chronic HCMV infection (9, 10, 51–53), was more frequently expressed on NK cells from some but not all cCMV-infected infants. “Atypical” or “adaptive-like” CD56^neg^ NK cells that function through FcγRIII-mediated ADCC rather than direct cytotoxicity (24, 54, 55) were more consistently expanded, consistent with prior observations in HCMV, EBV, and malaria infection in early life (21, 24, 54). CD56^neg^ NK cells highly express CD57 and LAG3, mirroring the transcriptional changes we observed in cCMV infection and suggesting this may be a comparable population. Our work suggests that NK cells capable of ADCC expand in utero following HCMV infection and whether they aid in antiviral control should be explored (56).

Our data provide insights into the influence of cCMV infection on host cellular immunity and also highlight an opportunity to protect neonates broadly using antibodies against infected, malignant, or autoimmune cells. Maternal IgG is actively transferred across the placenta via FcRn to protect infants in utero and during the first year of life (57–60). We speculate that FcRT cells may represent an additional effector cell population that can expand the cellular compartment to leverage maternal IgG in early life. Vaaben et al. recently proposed that FcγRIII-activating maternal IgG may synergize with neonatal NK cells to protect against HCMV via antibody-dependent mechanisms (21, 56), a hypothesis that our data further support. We recently reported that higher levels of FcγRIII and ADCC-activating IgG in maternal and cord blood sera were associated with protection against cCMV transmission in this same cohort (61, 62). Thomas et al. also found that higher ADCC-mediating antibody responses and viral susceptibility to ADCC were associated with decreased risk of HIV-1 transmission in utero (63). Together, these findings suggest that Fc-mediated responses linking maternal humoral and fetal cellular immunity may contribute to immune responses against congenital infections.

We propose that neonatal NK and FcRT cells could be targeted with monoclonal or polyclonal antibodies to elicit cellular Fc effector functions against HCMV and other pathogens. Maternal hyperimmune globulin treatment did not prevent fetal HCMV transmission in randomized clinical trials (64, 65), underscoring the unmet need to develop novel strategies to prevent transmission. Antibodies with Fc regions engineered to improve FcγR binding or bivalent antibodies to engage specific FcγR-expressing cells could be given to pregnant people or neonates. Nirsevimab, an anti–respiratory syncytial virus (anti-RSV) monoclonal antibody, with a modified Fc region, has recently been shown to be highly efficacious in protecting neonates from RSV (66), highlighting the promise of such emerging antibody-based therapeutics. More broadly, FcRT and NK cells capable of ADCC may be an underappreciated component of early life immunity that could be harnessed by vaccines against HCMV or pathogens like group B strep and *E*. *coli* that are a leading cause of neonatal sepsis. Stimulating the expansion of FcγR-expressing NK and T cells with vaccine adjuvants or specific antigens may allow better synergy between infant effector cells and maternal IgG. Additional work into how these cells are generated and their role in antipathogen defense is needed, but these future directions highlight their translational potential.

There are several limitations to our study that could be expanded upon in future work. Our retrospective cohort limited us from collecting additional clinical data and biospecimens. We were unable to test for HCMV viral loads in saliva or urine, as these samples were not collected so used DNAemia to define cCMV infection, which has been validated in several studies (67, 68). Due to cord blood bank protocols, samples from cCMV-infected infants born preterm, from a multiple gestation pregnancy, or with symptomatic disease at birth were not available and long-term clinical outcomes were not collected. Thus, we could not investigate how these immune changes relate to symptomatic disease or whether the gestational timing of transmission influenced fetal immune responses. Moreover, we could not determine how long these phenotypic and functional changes persist in the cellular compartment. Finally, banked cord blood sample volumes were extremely limited, so we could only perform functional studies on a subset of infants. Nevertheless, the data presented convincingly demonstrate that cCMV infection expands NK-like CD8^+^ T cells capable of antibody-dependent effector functions. Future studies should investigate how these immunological changes relate to antiviral control and clinical outcomes of cCMV infection and further characterize the origin, persistence, and functions of FcRT cells in early life.

In conclusion, we have demonstrated that cCMV infection expands FcγRIII-expressing CD8^+^ T cells, including canonical αβ and nontraditional γδ T cells, that have high cytotoxic potential and are poised to mediate ADCC. How FcRT and NK cells contribute to fetal defense against congenital infections must be explored further, but these populations represent promising translational targets to overcome the challenges to generating adaptive immune responses in early life. Altogether, our work suggests that CD8^+^ T and NK cells can mediate antibody effector functions through FcγR in early life and should be considered in antibody-based therapeutics and vaccination strategies to protect the infant.

## Methods

### Sex as a biological variable.

Our study included cord blood samples from male and female infants. Cases of cCMV infection and controls without cCMV infection were matched based on infant sex to account for any potential confounding when comparing groups. No differences in cord blood immunophenotypes by sex were observed in our PCA analysis of CCBB graft data, though subsequent experiments were not stratified by sex due to limited sample size.

### Human umbilical cord blood samples.

Cases and controls were identified from over 29,000 CCBB donor records (see Supplemental Figure 1 for an overview of sample selection). Maternal donors underwent infectious diseases screening for HCMV, hepatitis B virus, syphilis, hepatitis C virus, HIV-1/2, human T-lymphotropic virus (HTLV) I and II, Chagas disease, and West Nile virus. Only donors with healthy, uncomplicated pregnancies that gave birth at term were included and infants were screened for signs of (a) neonatal sepsis, (b) congenital infection (petechial rash, thrombocytopenia, hepatosplenomegaly), and (c) congenital abnormalities. Cord blood plasma was screened by the CCBB for HCMV viremia with a Real-Time PCR COBAS AmpliPrep/TaqMan Nucleic Acid Test (Roche Diagnostics). Cases of cCMV infection were defined as donors with cord blood that screened positive for HCMV DNAemia per PCR. Cases with cCMV infection (cCMV^+^, *n* = 59) were matched to at least 2 uninfected controls (cCMV^–^, *n* = 135) that did not have detectable HCMV DNAemia in the cord blood at birth. Matching variables included infant sex, race/ethnicity, maternal age (+/– 3 years), and delivery year (+/– 3 years), as in Supplemental Table 1.

### CCBB graft characterization.

Flow cytometry graft characterization was performed at the time of donation on fresh umbilical cord blood mononuclear cells (CBMCs) by the Duke Stem Cell Transplant Laboratory of Duke University Hospital, which provides contract services to the CCBB. Graft characterization data was then obtained retrospectively from the CCBB donor database. PCA plots of graft characterization data were rendered using ggplot2 (version 3.4.0) in R.

### NK and T cell phenotyping and sorting.

Flow cytometry was performed in the Duke Human Vaccine Institute (DHVI) Research Flow Cytometry Shared Resource Facility. For phenotyping, cryopreserved umbilical cord blood was thawed briefly at 37°C and resuspended in R10 media (RPM1 1640 with glutamine [Gibco, Thermo Fisher Scientific] plus 10% heat-inactivated FBS) with Benzonase (Millipore; 2 μl/mL). Resuspended cord blood was then pelleted at 25*g* for 5 minutes. Following pelleting, fetal RBCs were lysed with approximately 3 mL of RBC lysis buffer for 5 minutes, then washed with 1× PBS and pelleted at 25*g* for 5 minutes. CBMCs were then resuspended and enumerated on a Muse Cell Analyzer before being pelleted at 25*g* for 5 minutes and resuspended at 2.0 × 10^7^ cells/mL in 1% PBS/BSA. For phenotyping, 5–10 million cells (depending on viable cell count after cryopreservation) were stained with an optimized monoclonal antibody cocktail of fluorescently conjugated antibodies against surface markers for 30 minutes at 4°C. Antibodies in the general lineage panel included the following: CD14 pacific blue (M5E2, BioLegend), CD16 BV570 (3G8, BioLegend), CD25 BV605 (BC96, BioLegend), CD56 BV650 (HCD56, BioLegend), NKG2C BV711 (134591), CD45 BV786 (HI30, BD Biosciences), CD34 FITC (561, BioLegend), CD19 BB700 (HIB19, BD Biosciences), NKG2A PE (S19004C, BioLegend), CD235a PE-Cy-5 (HIR2, BioLegend), CD3 PE-Texas Red (7D6, Thermo Fisher), CD127 PE-Cy7 (A019D5, BioLegend), CD8 APC (RPA-T8, BD Biosciences), HLA-DR AF700 (L243, BioLegend), and CD4 APC-H7 (SK3, BD Biosciences). Antibodies in the T and NK cell panel included the following: CD3 BV421 (UCHT1, BD Biosciences), CD8 BV570 (RPA-T8, BioLegend), CCR7 BV605 (G043H7, BioLegend), CD56 BV650 (HCD56, BioLegend), PD-1 BV785 (EH12.2H7, BioLegend), TCRγ/δ FITC (11F2, BD Biosciences), CD45RA PerCP-Cy5.5 (HI100, BioLegend), NKG2C PE (S19005E, BioLegend), CD57 PE-CF594 (NK-1, BD Biosciences), CD235a PE-Cy5 (HIR2, BioLegend), CD16 PE-Cy7 (3G8, BD Biosciences), NKG2A PE-Cy5 (HIR2, BioLegend), and CD4 AF700 (L200, BD Biosciences). Antibodies in the TCR panel included the following: γ/δ TCR FITC (5A6.E9, Invitrogen), α/β TCR APC (IP26, BioLegend) in a panel with antibodies against iNKT BV421 (6B11, BioLegend), CD14 BV500 (M5E2, BD Biosciences), CD19 BV500 (HIB19, BD Biosciences), PD1 BV605 (EH12.2H7, BD Biosciences), CD45RA BV711 (HI100, BD Biosciences), CD4 BV785 (OKT4, BioLegend), CD16 PE (3g8, BioLegend), NKG2D PE-CF596 (1d11, BD Biosciences), CCR7 Pe-Cy5 (G043H7, BioLegend), CD8 PerCP Cy5.5 (SK1, BioLegend), CD56 Pe-Cy7 (NCAM162, BD Biosciences), CD69 AF700 (FN50, BioLegend), and CD3 APC-H7 (SK7, BD Biosciences). Cells were then washed with PBS and pelleted at 25*g* for 5 minutes and resuspended in live/dead Aqua (Thermo Fisher) or near infrared (IR) (Invitrogen) stain and incubated at room temperature for 20 minutes. Fluorescence minus one (FMO) control tubes were included for CD34, CD16, CD56, CD127, HLA-DR, TCR γ/δ, CCR7, CD45RA, NKG2A, NKG2C, CD57, and PD-1 for downstream manual gating. Single-color AbC or ArC beads (Invitrogen) for each antibody and live/dead stain were used as compensation controls. Flow cytometry data were acquired on a FACSAria (BD Biosciences) instrument using FACSDiva (version 8.0) and analyzed in FlowJo (version 10.8.1).

### CITRUS analysis.

tSNE-CUDA dimensionality reduction and CITRUS analyses (26) were completed in Cytobank, a cloud-based bioinformatics platform for analyzing high dimensional cytometry data (Beckman Coulter; www.cytobank.org). All samples were pregated on live, CD235-negative cells before FCS files before downstream analyses. For the tSNE-CUDA analysis, 400,000 live CD235^–^ events were sampled per sample FCS file and perplexity was set to 40. For the general lineage panel CITRUS analysis, 50,000 live CD235^–^ events were sampled per sample FCS file (*n* = 25 CBMCs, total cell events = 1,250,000) and the minimum cluster size was set to 1% of total events. CD3, CD4, CD8, CD127, CD25, CD19, CD56, CD16, NKG2A, NKG2C, HLA-DR, and CD14 marker expression was normalized across all samples, then used as channels for clustering. For the T and NK cell panel CITRUS analysis, 75,000 live CD235^–^ events were sampled per sample FCS file (*n* = 16 CBMCs, total cell events = 1,200,000), and the minimum cluster size was set to 1% of total events. CD3, CD4, CD8, CD56, CD16, γδ TCR, CCR7, CD45RA, PD-1, CD57, NKG2A, and NKG2C marker expression was normalized across all samples, then used as channels for clustering. SAM, a correlative association model, was used to identify cell clusters that differed in abundance (significance cut-off FDR *P* < 0.01) between cCMV^+^ and cCMV^–^ groups.

### RNA-Seq sample preparation and analysis.

T and NK cell subsets were FAC-sorted directly into RLT lysis buffer (Qiagen), and total RNA was extracted using the RNeasy Micro Kit (Qiagen, catalog 74004). Total CD4^+^ T, CD8^+^ T, and NK cells were sorted from 25 unique cord blood samples (*n* = 13 cCMV^+^, *n* = 12 cCMV^–^); however, several samples failed RNA quality control and were excluded from downstream transcriptional analyses. CD16^–^CD8^+^ and CD16^+^CD8^+^ T cell subsets were sorted from a total of 16 unique cord blood samples (*n* = 8 cCMV^+^, *n* = 8 cCMV^–^), and 1 sample from the cCMV^–^ group failed quality control and was excluded from downstream transcriptional analyses. RNA quality was evaluated by RNA integrity number (RIN) (minimum cut-off > 8.5) prior to library preparation by the DHVI Sequencing Core Facility. Briefly, full-length cDNAs were generated using up to 10 ng of total RNA through the SMART-Seq v4 Ultra Low Input Kit for Sequencing (Takara, catalog 634891). Total 200 pg cDNAs were used to generate the dual index Illumina libraries using the Nextera XT DNA Library Prep Kit (Illumina, catalog FC-131-1096). Sequencing was performed on an Illumina NextSeq 500 sequencer to generate 2′ 76 paired-end reads using the TG NextSeq 500/550 High Output Kit, version 2.5 (150 cycles), following the manufacturer’s protocol (Illumina, catalog 20024912). The quality of cDNAs and Illumina libraries was assessed on a TapeStation 2200 with the high-sensitivity D5000 ScreenTape (Agilent, catalog 5067-5592), and their quantity was determined by Qubit 3.0 fluorometer (Thermo Fisher). Gene reads were aligned to the human reference genome GRCh38 using QIAGEN CLC genomics (version 20). DEseq2 (v1.38.3) was used to normalize count data and perform differential gene expression analysis. Genes were considered differentially expressed based on a 1.2 log_2_fold change in gene expression and FDR *P* < 0.1. PCA was performed on rlog-transformed data using the plotPCA function in DESeq2. Volcano plots were generated using the EnhancedVolcano (version 1.16.0) package in R. Heatmaps and hierarchical clustering were performed on rlog-normalized DEseq2data using the ComplexHeatmap (version 2.14.0) package in R. Gene set enrichment analysis of differentially expressed GO pathways (minimum 5, maximum 2000 genes) was performed using iDEP, version 0.96 (69), with a significance cut-off of FDR *P* < 0.2. Genes encoding TFs were identified by matching DE gene IDs to a list of human TFs from AnimalTFDB, version 4.0 (14, 70).

### Functional immunological assays.

Antibody-dependent degranulation of NK and CD8^+^ T cells was performed as follows. Cord blood was thawed at 37°C, diluted 1:4 in RPMI supplemented with 10% FBS, penicillin, streptomycin, l-glutamine, and gentamicin (complete media), and processed using Ficoll separation. Following separation, the cells were counted and rested overnight at a concentration of 2–3 million cells per mL in either complete media or complete media supplemented with 10 ng/mL of IL-15. After resting overnight, the cells were counted and resuspended at 5 million/mL. CBMCs in bulk are referred to as “effector” cells and CEM.NKRs coated with 5 μg/mL BAL gp120 are referred to as “target” cells. CBMCs were either plated alone (effector only), with a 10:1 ratio with targets (effector+target), and with 1 μg/mL of Synagis (effector+targets+synagis) or 1 μg/mL of a mixture of 4 optimized HIV antibodies (effector+target+mAb mix). The HIV antibody cocktail comprised 250 ng/mL each of 7B2_AAA, 2G12_AAA, A32_AAA, and CH44_AAA, which contains the AAA optimization for Fc-mediated activity. All 4 conditions were plated for 6 hours in the presence of 1 μg/mL brefeldin (BD GolgiPlug), 1 μg/mL Monensin A (BD GolgiStop), and anti-CD107a antibody (BioLegend H4A3). After 6 hours, the cells were washed with DPBS and stained with LIVE/DEAD viability stain (Thermo Fisher), then washed and stained for the following surface markers: CD14 V500 (M5E2, BD Biosciences), CD19 V500 (HIB19, BD Biosciences), CD57 BV711 (QA17A04, BioLegend), CD4 BV785 (OKT4, BioLegend), CD45RA FITC (5H9, BD Biosciences), CD16 PE (3g8, BioLegend), CCR7 Pe-Cy5 (G043H7, BioLegend), CD8 PerCP Cy5-5 (SK1, BioLegend), CD56 PeCy7 (NCAM16.2, BD Biosciences), and CD3 APC-Cy7 (SK7, BD Biosciences). Next, cells were fixed with Cytofix/Cytoperm (BD Biosciences), then stained for the following intracellular markers: PRF1 PacBlue (dg9, BioLegend), IFN-γ APC (4S.B3, BioLegend), and granzyme B AF700 (QA16A02, BioLegend) in the presence of Perm/Wash Buffer (BD Biosciences). Samples were acquired on a LSRFortessa II (BD Biosciences) using FACSDiva, version 8.0, software. Frequency of NK and CD8^+^ T cells expressing granzyme B and PRF1 was measured in the effector-only condition. Percentage change in IFN-γ and CD107a was calculated by subtracting the frequencies in the effector+target conditions from the anti-RSV and anti-HIV antibody conditions, respectively, with the dot plots showing the background subtracted data points. Data were analyzed using FlowJo, version 10.8.

### Statistics.

All statistical analyses were completed in R (version 4) or GraphPad Prism (version 9 and version 10). Frequencies of immune cell populations or normalized gene expression data were compared using Mann-Whitney *U*/Wilcoxon rank-sum tests for pair-wise comparisons and ANOVA with a post-hoc Tukey’s test for comparisons across multiple groups. Statistical significance was defined as *P* < 0.05 with a 2-tailed test and FDR correction for multiple comparisons. Specific details on each statistical analysis performed and the exact *n* are available in the respective figure legends. Additional details on the statistical significance thresholds for the CITRUS and RNA-Seq analyses are described in the methods section above. Inclusion and exclusion criteria for the study are described above and outlined in Supplemental Figure 1, and randomization for case-control matching was achieved using a random number generator.

### Study approval.

Our study included cases of cCMV and controls without cCMV infection that were recruited from 2008–2017 as donors to the CCBB. Approval was obtained from Duke’s Institutional Review Board (Pro00089256) to use deidentified clinical data and biospecimens provided by the CCBB. No patients were prospectively recruited for this study, and all cord blood was acquired retrospectively from the CCBB biorepository from donors who provided written consent for biospecimens to be used for research.

### Data availability.

Requests for data, resources and reagents should be directed to the authors. RNA-Seq read count data and code have been deposited at Zenodo (https://doi.org/10.5281/zenodo.8323011) and are publicly available; however, the CCBB consent documents under which these samples were originally collected do not contain any language that would authorize depositing identifiable genomic data, such as FASTQ files from RNA-Seq, into a repository for data sharing. Importantly, the CCBB consent forms were submitted and approved years before the NIH data management and sharing policy went into effect. Individual data points presented in the manuscript are available in the Supporting Data Values file. Additional information required to reanalyze the data reported in this paper is available from upon request.

## Author contributions

ECS, DRN, JP, SRP, and KMW conceived the project. ECS, TDB, CBC, RKR, JP, SRP, and KMW devised the methodology. ECS, DRN, and ANN performed the experiments. ECS and DRN curated data and completed the formal analysis and visualization. ECS, CBC, and KMW assisted with the software analysis. ECS, CBC, JHH, SRP, and KMW acquired funding. ECS and JHH assisted with project administration. DWC, RKR, JK, and CBC provided resources. JP, SRP, and KMW supervised the project. ECS and DRN wrote the original draft of the manuscript. ECS, DRN, ANN, JHH, DWC, TDB, JK, RKR, CBC, GGF, JP, SRP, and KMW reviewed and edited the manuscript.

## Supplementary Material

Supplemental data

Supporting data values

## Version 1 11/12/2024

In-Press Preview

## Version 2 01/02/2025

Electronic publication

## References

[1] Cadwell K (2015). The virome in host health and disease. Immunity.

[2] Davis MM, Brodin P (2018). Rebooting human immunology. Annu Rev Immunol.

[3] Zuhair M (2019). Estimation of the worldwide seroprevalence of cytomegalovirus: a systematic review and meta-analysis. Rev Med Virol.

[4] Brodin P (2015). Variation in the human immune system is largely driven by non-heritable influences. Cell.

[5] Semmes EC (2020). Cytomegalovirus as an immunomodulator across the lifespan. Curr Opin Virol.

[6] Boppana SB (2013). Congenital cytomegalovirus infection: clinical outcome. Clin Infect Dis.

[7] Furman D (2015). Cytomegalovirus infection enhances the immune response to influenza. Sci Transl Med.

[8] Rolle A, Brodin P (2016). Immune adaptation to environmental influence: the case of NK cells and HCMV. Trends Immunol.

[9] Guma M (2004). Imprint of human cytomegalovirus infection on the NK cell receptor repertoire. Blood.

[10] Schlums H (2015). Cytomegalovirus infection drives adaptive epigenetic diversification of NK cells with altered signaling and effector function. Immunity.

[11] Almanzar G (2005). Long-term cytomegalovirus infection leads to significant changes in the composition of the CD8+ T-cell repertoire, which may be the basis for an imbalance in the cytokine production profile in elderly persons. J Virol.

[12] Sylwester AW (2005). Broadly targeted human cytomegalovirus-specific CD4+ and CD8+ T cells dominate the memory compartments of exposed subjects. J Exp Med.

[13] Remmerswaal EBM (2019). Expression of IL-7Rα and KLRG1 defines functionally distinct CD8^+^ T-cell populations in humans. Eur J Immunol.

[14] Sottile R (2021). Human cytomegalovirus expands a CD8^+^ T cell population with loss of *BCL11B* expression and gain of NK cell identity. Sci Immunol.

[15] Couzi L (2012). Antibody-dependent anti-cytomegalovirus activity of human γδ T cells expressing CD16 (FcγRIIIa). Blood.

[16] Marchant A (2003). Mature CD8(+) T lymphocyte response to viral infection during fetal life. J Clin Invest.

[17] Huygens A (2015). Functional exhaustion limits CD4+ and CD8+ T-cell responses to congenital cytomegalovirus infection. J Infect Dis.

[18] Huygens A (2014). Immunity to cytomegalovirus in early life. Front Immunol.

[19] Antoine P (2012). Functional exhaustion of CD4+ T lymphocytes during primary cytomegalovirus infection. J Immunol.

[20] Vermijlen D (2010). Human cytomegalovirus elicits fetal gammadelta T cell responses in utero. J Exp Med.

[21] Vaaben AV (2022). In utero activation of natural killer cells in congenital cytomegalovirus infection. J Infect Dis.

[22] Kollmann TR (2017). Protecting the newborn and young infant from infectious diseases: lessons from immune ontogeny. Immunity.

[23] Semmes EC (2020). Understanding early-life adaptive immunity to guide interventions for pediatric health. Front Immunol.

[24] Ty M (2023). Malaria-driven expansion of adaptive-like functional CD56-negative NK cells correlates with clinical immunity to malaria. Sci Transl Med.

[25] Rückert T (2022). Clonal expansion and epigenetic inheritance of long-lasting NK cell memory. Nat Immunol.

[26] Bruggner RV (2014). Automated identification of stratifying signatures in cellular subpopulations. Proc Natl Acad Sci U S A.

[27] Chan DM (2019). GPU accelerated t-distributed stochastic neighbor embedding. J Parallel Distrib Comput.

[28] Pietra G (2003). HLA-E-restricted recognition of cytomegalovirus-derived peptides by human CD8+ cytolytic T lymphocytes. Proc Natl Acad Sci U S A.

[29] Mazzarino P (2005). Identification of effector-memory CMV-specific T lymphocytes that kill CMV-infected target cells in an HLA-E-restricted fashion. Eur J Immunol.

[30] Clémenceau B (2008). Effector memory alphabeta T lymphocytes can express FcgammaRIIIa and mediate antibody-dependent cellular cytotoxicity. J Immunol.

[31] Björkström NK (2008). Elevated numbers of Fc gamma RIIIA+ (CD16+) effector CD8 T cells with NK cell-like function in chronic hepatitis C virus infection. J Immunol.

[32] Naluyima P (2019). Terminal effector CD8 T cells defined by an IKZF2^+^IL-7R^–^ transcriptional signature express FcγRIIIA, expand in HIV infection, and mediate potent HIV-specific antibody-dependent cellular cytotoxicity. J Immunol.

[33] Chung AW (2009). Rapid degranulation of NK cells following activation by HIV-specific antibodies. J Immunol.

[34] Jacquemont L (2020). Terminally differentiated effector memory CD8^+^ T cells identify kidney transplant recipients at high risk of graft failure. J Am Soc Nephrol.

[35] Galindo-Albarrán AO (2016). CD8^+^ T cells from human neonates are biased toward an innate immune response. Cell Rep.

[36] Ng SS (2020). The NK cell granule protein NKG7 regulates cytotoxic granule exocytosis and inflammation. Nat Immunol.

[37] Malarkannan S (2020). NKG7 makes a better killer. Nat Immunol.

[38] Phaahla NG (2019). Chronic HIV-1 infection alters the cellular distribution of FcγRIIIa and the functional consequence of the FcγRIIIa-F158V variant. Front Immunol.

[39] Choi SJ (2023). KIR^+^CD8^+^ and NKG2A^+^CD8^+^ T cells are distinct innate-like populations in humans. Cell Rep.

[40] Koh JY (2023). Human CD8^+^ T-cell populations that express natural killer receptors. Immune Netw.

[41] Intlekofer AM (2005). Effector and memory CD8+ T cell fate coupled by T-bet and eomesodermin. Nat Immunol.

[42] Wong P (2023). T-BET and EOMES sustain mature human NK cell identity and antitumor function. J Clin Invest.

[43] Goh W (2020). Hhex directly represses BIM-dependent apoptosis to promote NK cell development and maintenance. Cell Rep.

[44] Wu Z (2021). Human cytomegalovirus infection promotes expansion of a functionally superior cytoplasmic CD3+ NK cell subset with a Bcl11b-regulated T cell signature. J Immunol.

[45] Holmes TD (2021). The transcription factor Bcl11b promotes both canonical and adaptive NK cell differentiation. Sci Immunol.

[46] Li P (2010). Reprogramming of T cells to natural killer-like cells upon Bcl11b deletion. Science.

[47] Farrington LA (2020). Opsonized antigen activates Vδ2+ T cells via CD16/FCγRIIIa in individuals with chronic malaria exposure. PLoS Pathog.

[48] Farrington LA (2015). Frequent malaria drives progressive Vδ2 T-cell loss, dysfunction, and CD16 up-regulation during early childhood. J Infect Dis.

[49] Angelini DF (2004). FcgammaRIII discriminates between 2 subsets of Vgamma9Vdelta2 effector cells with different responses and activation pathways. Blood.

[50] Roy Chowdhury R (2023). NK-like CD8^+^ γδ T cells are expanded in persistent Mycobacterium tuberculosis infection. Sci Immunol.

[51] Guma M (2006). Expansion of CD94/NKG2C+ NK cells in response to human cytomegalovirus-infected fibroblasts. Blood.

[52] Monsivais-Urenda A (2010). Influence of human cytomegalovirus infection on the NK cell receptor repertoire in children. Eur J Immunol.

[53] Noyola DE (2012). Influence of congenital human cytomegalovirus infection and the NKG2C genotype on NK-cell subset distribution in children. Eur J Immunol.

[54] Forconi CS (2018). Poorly cytotoxic terminally differentiated CD56^neg^CD16^pos^ NK cells accumulate in Kenyan children with Burkitt lymphomas. Blood Adv.

[55] Forconi CS (2020). A new hope for CD56^neg^CD16^pos^ NK cells as unconventional cytotoxic mediators: an adaptation to chronic diseases. Front Cell Infect Microbiol.

[56] Semmes EC, Permar SR (2022). Human cytomegalovirus infection primes fetal natural killer cells for Fc-mediated antiviral defense. J Infect Dis.

[57] Jennewein MF (2019). Fc glycan-mediated regulation of placental antibody transfer. Cell.

[58] Martinez DR (2019). Fc characteristics mediate selective placental transfer of IgG in HIV-infected women. Cell.

[59] Jennewein MF (2017). Transfer of maternal immunity and programming of the newborn immune system. Semin Immunopathol.

[60] Fouda GG (2018). The impact of IgG transplacental transfer on early life immunity. Immunohorizons.

[61] Semmes EC (2023). ADCC-activating antibodies correlate with decreased risk of congenital human cytomegalovirus transmission. JCI Insight.

[62] Semmes EC (2022). Maternal Fc-mediated non-neutralizing antibody responses correlate with protection against congenital human cytomegalovirus infection. J Clin Invest.

[63] Thomas AS (2022). Antibody-dependent cellular cytotoxicity responses and susceptibility influence HIV-1 mother-to-child transmission. JCI Insight.

[64] Hughes BL (2021). A trial of hyperimmune globulin to prevent congenital cytomegalovirus infection. N Engl J Med.

[65] Revello MG (2014). A randomized trial of hyperimmune globulin to prevent congenital cytomegalovirus. N Engl J Med.

[66] Slomski A (2022). Long-acting RSV antibody injection protects healthy infants. JAMA.

[67] Kaye T, Lynfield R (2024). Notes from the field: universal newborn screening and surveillance for congenital cytomegalovirus - Minnesota, 2023-2024. MMWR Morb Mortal Wkly Rep.

[68] Roback JD (2005). Comparison of cytomegalovirus polymerase chain reaction and serology for screening umbilical cord blood components. Transfusion.

[69] Ge SX (2018). iDEP: an integrated web application for differential expression and pathway analysis of RNA-Seq data. BMC Bioinformatics.

[70] Shen WK (2023). AnimalTFDB 4.0: a comprehensive animal transcription factor database updated with variation and expression annotations. Nucleic Acids Res.

