# Supplementary material for: In utero human cytomegalovirus infection expands NK-like FcγRIII+CD8+ T cells that mediate Fc antibody functions
Source: J Clin Invest. 2024 Nov 12;135(1):e181342. doi: 10.1172/JCI181342 (PMC11684805; doi:10.1172/JCI181342)
Supplement: Supplemental data [file jci-135-181342-s207.pdf]

Supplementary Figure 1. Identification of cases and controls from the Carolinas Cord Blood Bank (CCBB).

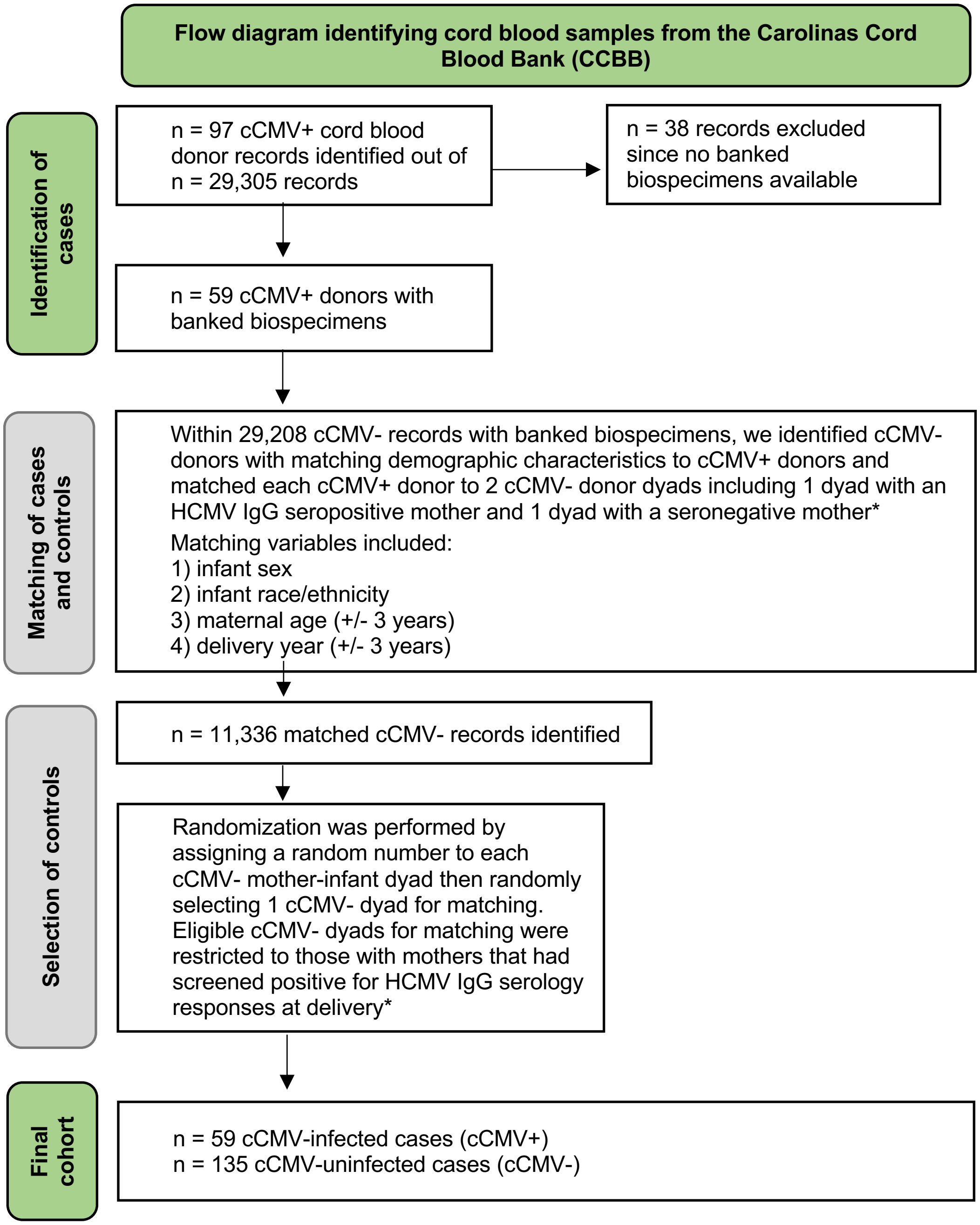

cCMV = congenital cytomegalovirus (CMV) infection
cCMV+ = positive CMV PCR cord blood screening at birth
cCMV- = negative CMV PCR cord blood screening at birth

*Initial HCMV serology screening performed at time of donation by the American Red Cross in Charlotte, N.C.

Supplementary Table 1. Maternal and neonatal cord blood bank donor characteristics [a]

	cCMV+ (n = 59)	cCMV- (n = 135)
**Infant sex, n (%)**		
**Female**	24 (40.7)	52 (38.1)
**Male**	35 (59.3)	83 (61.9)
**Infant race/ethnicity, n (%)**		
**White**	32 (54.2)	75 (55.6)
**Black**	14 (23.7)	28 (20.7)
**Hispanic**	7 (11.9)	16 (11.9)
**Multiple**	1 (1.7)	0 (0.0)
**Other**	5 (8.5)	16 (11.9)
**Maternal age (years), median [IQR]**	27 [22-31]	30 [27-34]
**Gestational age (weeks), median [IQR]**	39 [38-40]	39 [39-40]
**Delivery year, median [IQR]**	2011 [2009-2015]	2014 [2010-2017]
**Delivery type, n (%)**		
**Vaginal**	23 (39.0])	75 (55.6)
**Cesarean section**	36 (61.0)	60 (44.4)
**Cord blood HCMV viral load, median [range] [b]**	727 [137-18,100]	ND

cCMV = cCMV infection; ND = not detected

cCMV-infected (cCMV+) indicates mother-infant pair with cCMV infection

cCMV-uninfected (cCMV-) indicates mother-infant pair without cCMV infection

[a] cCMV+ and cCMV- mother-infant pairs were matched on maternal age (+/- 3 years), infant race, sex, and delivery year (+/- 3 years)

[b] Cord blood HCMV viral copies listed in IU/mL, lower limit of detection = 137 copies/mL

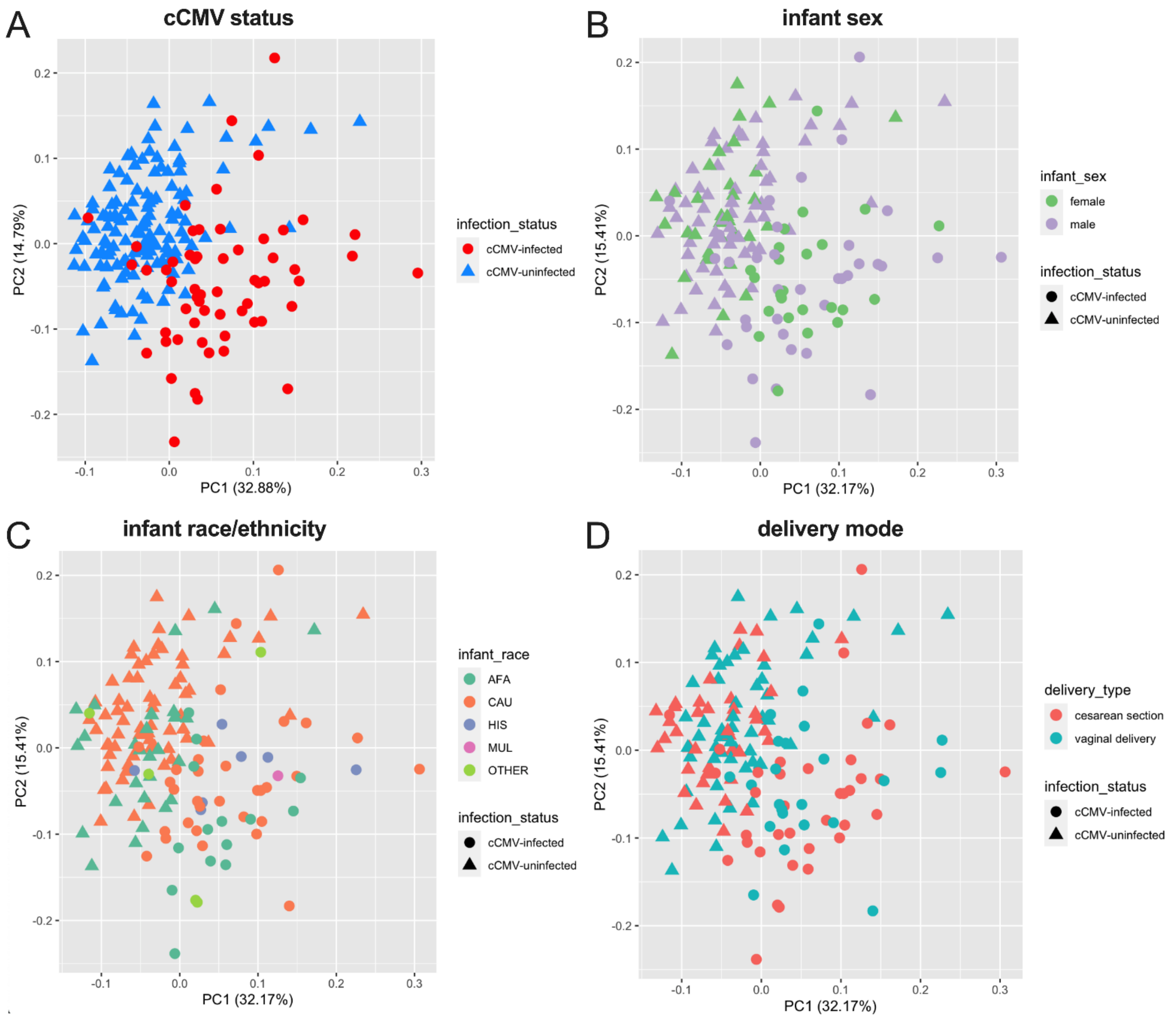

Supplementary Figure 2. Principal components analysis (PCA) of umbilical cord blood from cCMV-infected compared to cCMV-uninfected neonates stratified by clinical characteristics. PCA of 18 umbilical cord blood immune variables from CCBB cord blood graft characterization. (A) PCA colored by cCMV infection status. (B) PCA colored by infant sex. (C) PCA colored by infant race/ethnicity. (D) PCA colored by delivery mode. n = 59 cCMV-infected (cCMV+, circles), n = 135 cCMV-uninfected (cCMV-, triangles) neonates.

Supplementary Figure 3. Flow and Venn diagrams of study design with sample numbers.

Flow cytometry analyses

RNA seq analyses

Panel 1. cord blood samples

n = 13 cCMV+
n = 12 cCMV-

(1 excluded due to low cell count/ viability)

CITRUS MAP 1

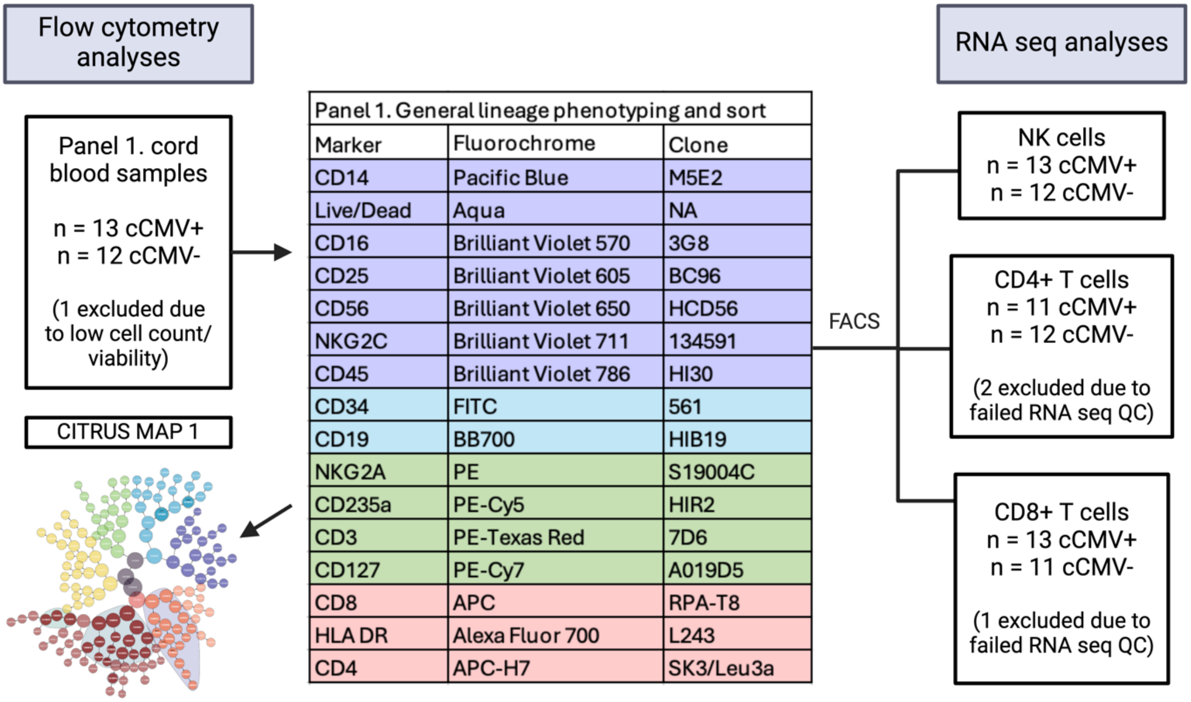

Panel 1. General lineage phenotyping and sort		
Marker	Fluorochrome	Clone
CD14	Pacific Blue	M5E2
Live/Dead	Aqua	NA
CD16	Brilliant Violet 570	3G8
CD25	Brilliant Violet 605	BC96
CD56	Brilliant Violet 650	HCD56
NKG2C	Brilliant Violet 711	134591
CD45	Brilliant Violet 786	HI30
CD34	FITC	561
CD19	BB700	HIB19
NKG2A	PE	S19004C
CD235a	PE-Cy5	HIR2
CD3	PE-Texas Red	7D6
CD127	PE-Cy7	A019D5
CD8	APC	RPA-T8
HLA DR	Alexa Fluor 700	L243
CD4	APC-H7	SK3/Leu3a

FACS

NK cells
n = 13 cCMV+
n = 12 cCMV-

CD4+ T cells
n = 11 cCMV+
n = 12 cCMV-

(2 excluded due to failed RNA seq QC)

CD8+ T cells
n = 13 cCMV+
n = 11 cCMV-

(1 excluded due to failed RNA seq QC)

Panel 2. cord blood samples

n = 8 cCMV+
n = 8 cCMV-

CITRUS MAP 2

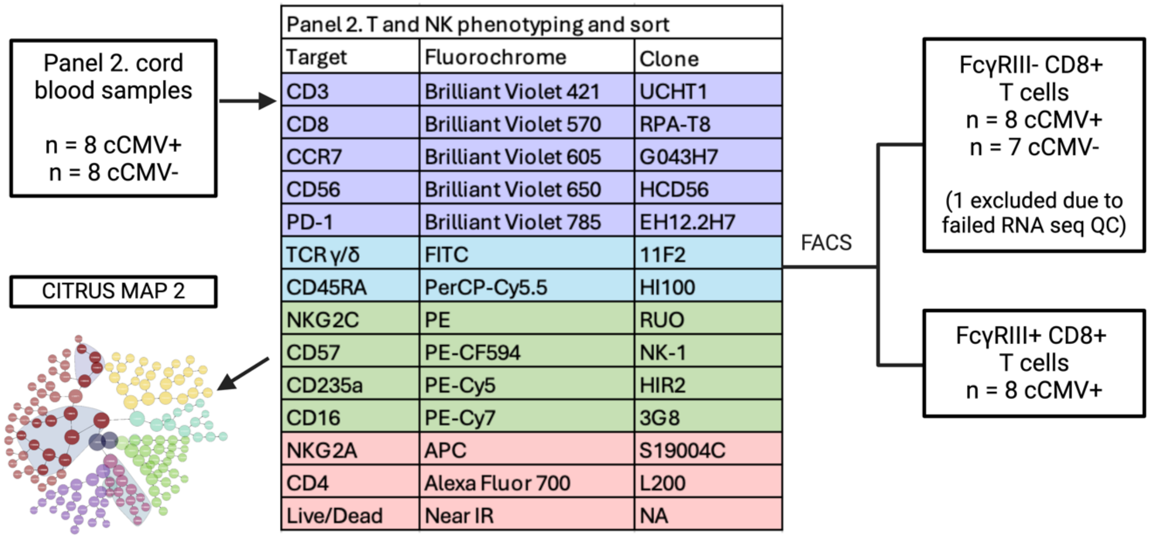

Panel 2. T and NK phenotyping and sort		
Target	Fluorochrome	Clone
CD3	Brilliant Violet 421	UCHT1
CD8	Brilliant Violet 570	RPA-T8
CCR7	Brilliant Violet 605	G043H7
CD56	Brilliant Violet 650	HCD56
PD-1	Brilliant Violet 785	EH12.2H7
TCR γ/δ	FITC	11F2
CD45RA	PerCP-Cy5.5	HI100
NKG2C	PE	RUO
CD57	PE-CF594	NK-1
CD235a	PE-Cy5	HIR2
CD16	PE-Cy7	3G8
NKG2A	APC	S19004C
CD4	Alexa Fluor 700	L200
Live/Dead	Near IR	NA

FACS

FcγRIII- CD8+ T cells
n = 8 cCMV+
n = 7 cCMV-

(1 excluded due to failed RNA seq QC)

FcγRIII+ CD8+ T cells
n = 8 cCMV+

Panel 1 + Panel 2. cord blood samples

n = 21 cCMV+
n = 20 cCMV-

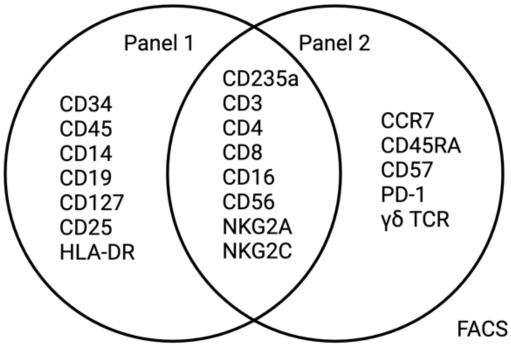

cCMV+ = CMV positive cord blood
cCMV- = CMV negative cord blood

CITRUS = cluster identification, characterization, and regression

FACS = Fluorescence-activated Cell Sorting

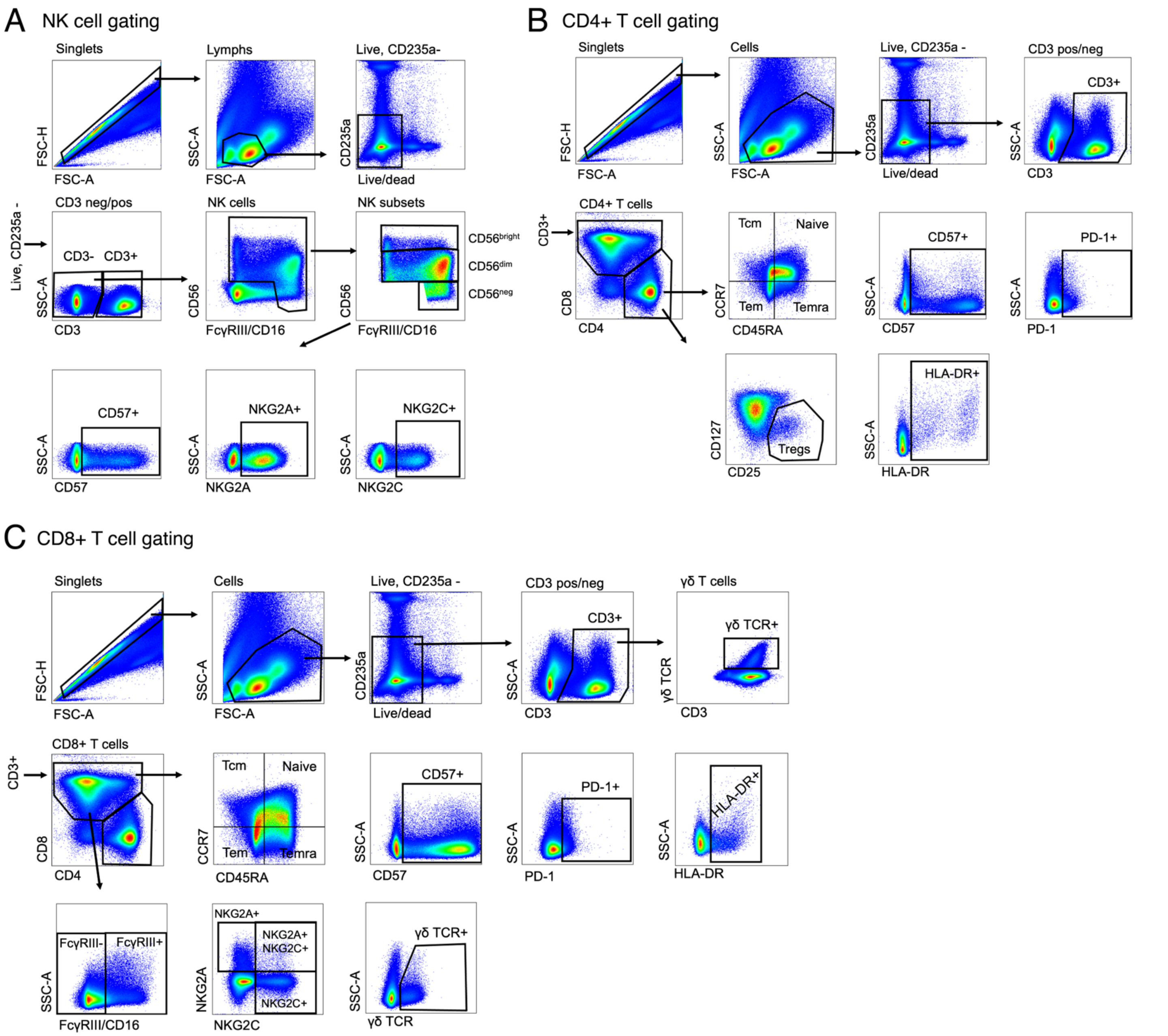

Supplementary Figure 4. Flow cytometry gating strategies. Boolean gating strategies to identify (A) NK cell, (B) CD4+ T cell, (C) CD8+ T and γδ T cells. Gating based on florescence minus one (FMO) controls.

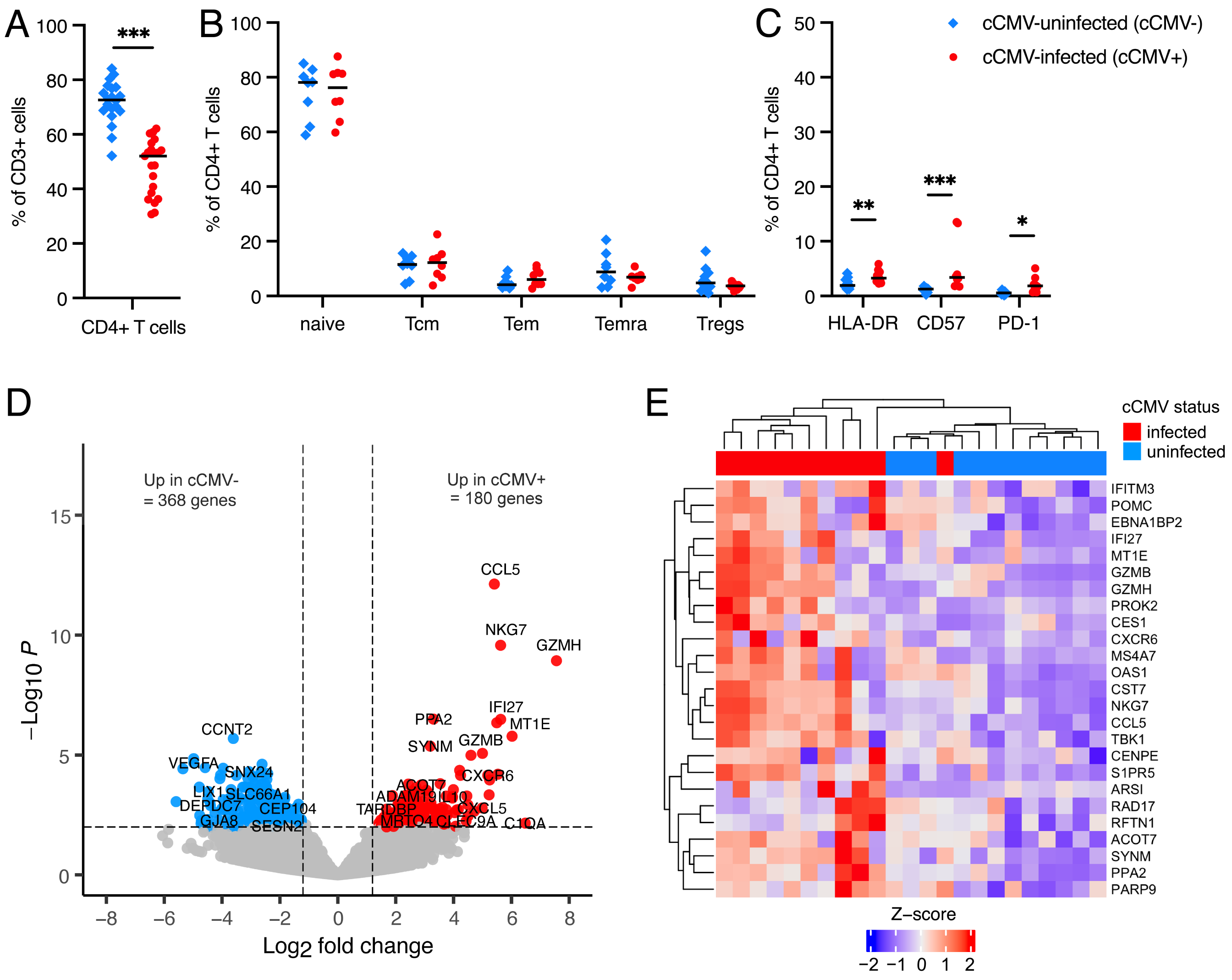

Supplementary Figure 5. Minor subset of CD4+ T cells with cytotoxic potential elicited in cCMV infection (A-C) CD4+ T cells were compared in cord blood from cCMV-infected (cCMV+, red circles) versus cCMV-uninfected (cCMV-, blue diamonds) neonates. (A-C) Frequency of total CD4+ T cells and CD4+ T cell subsets in cord blood from cCMV+ (n=21 total) versus cCMV- (n=20 total) neonates. (B) Frequency of total, naïve, central memory (Tcm), effector memory (Tem), terminally differentiated effector memory cells re-expressing CD45RA (Temra), and regulatory T cells (Tregs). (C) Frequency of total CD4+ T cells expressing HLA-DR, CD57, and PD-1 in cCMV+ (n=8) versus cCMV- (n=8) neonates. (D-E) RNA-seq analysis of FAC-sorted total CD4+ T cells from cCMV+ (n=11) and cCMV- (n=12) neonates. (D) Volcano plot of differentially expressed genes ($P < 0.01$, log2foldchange +/- 1.2). Red circles indicate genes enriched in cCMV+ CD4+ T cells, blue circles indicate genes enriched in cCMV- CD4+ T cells, and grey circles indicate genes whose expression did not differ significantly. (E) Heatmap of top 25 enriched genes (FDR $P < 0.1$, log2foldchange > 1.2). Z-score shows gene expression based on rlog-transformed data. FDR-corrected P values reported for Mann-Whitney U test. *$P < 0.05$, **$P < 0.01$, ***$P < 0.001$.

Supplementary Table 2. Gene set enrichment analysis (GSEA) comparing transcriptome of cord blood CD8+ T cells from cCMV-infected versus cCMV-uninfected neonates[a]

Direction	adjusted *P* value	Genes	Gene set enrichment analysis (GSEA) pathways
Down	4.90E-03	22	Regulation of axon extension involved in axon guidance
Up	1.30E-03	49	Chemokine-mediated signaling pathway
	1.30E-03	57	Response to chemokine
	1.30E-03	57	Cellular response to chemokine
	1.30E-03	131	Cellular response to interferon-gamma
	1.30E-03	57	Chromosome condensation
	1.30E-03	33	Monocyte chemotaxis
	1.30E-03	32	Lymphocyte chemotaxis
	1.30E-03	115	Nucleosome assembly
	1.30E-03	175	DNA packaging
	1.30E-03	31	DNA replication-dependent nucleosome assembly
	1.30E-03	31	DNA replication-dependent nucleosome organization
	1.30E-03	53	Natural killer cell mediated immunity[b]
	1.30E-03	75	Regulation of megakaryocyte differentiation
	1.30E-03	34	Regulation of natural killer cell mediated immunity
	1.30E-03	50	Natural killer cell mediated cytotoxicity
	2.10E-03	37	Nucleolar chromatin organization
	1.30E-03	31	Regulation of natural killer cell mediated cytotoxicity
	2.10E-03	36	DNA heterochromatin assembly
	1.30E-03	42	Nucleolus organization
	1.30E-03	88	Leukocyte mediated cytotoxicity
	1.30E-03	149	Response to interferon-gamma
	2.90E-03	25	Positive regulation of natural killer cell mediated immunity
	1.30E-03	126	Cell killing
	1.30E-03	154	Nucleosome organization
	1.30E-03	135	Chromatin assembly
	3.00E-03	13	Positive regulation of lymphocyte chemotaxis
	3.60E-03	16	Pyroptosis
	5.80E-03	21	Positive regulation of natural killer cell mediated cytotoxicity
	2.30E-03	10	Eosinophil migration

cCMV = congenital CMV infection

[a] includes n=13 cCMV+ and n=12 cCMV- neonates

[b] pathways involved in NK cell responses highlighted in yellow

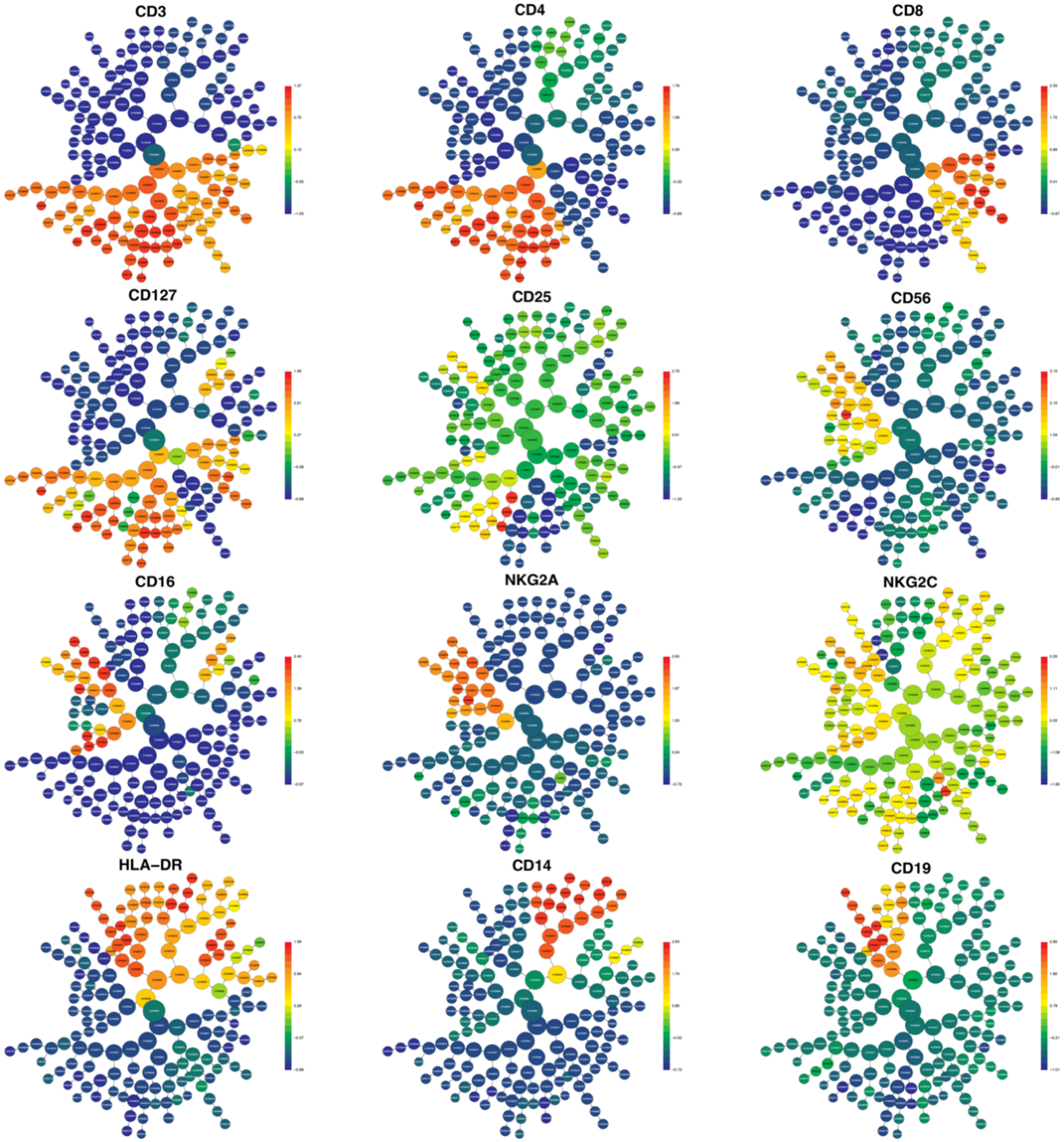

Supplementary Figure 6. CITRUS cluster map for general lineage phenotyping panel. Unsupervised cluster identification, characterization, and regression (CITRUS) was used to cluster immune cell populations based on CD3, CD4, CD8, CD127, CD25, CD19, CD56, CD16, NKG2A, NKG2C, HLA-DR and CD14 marker expression. CITRUS cluster map generated from 1,250,000 live, CD235a- singlet events including 50,000 events per sample (n=25 samples) with a minimum cluster size of 1%. See additional supplementary data for high resolution pdf of marker expression by cluster.

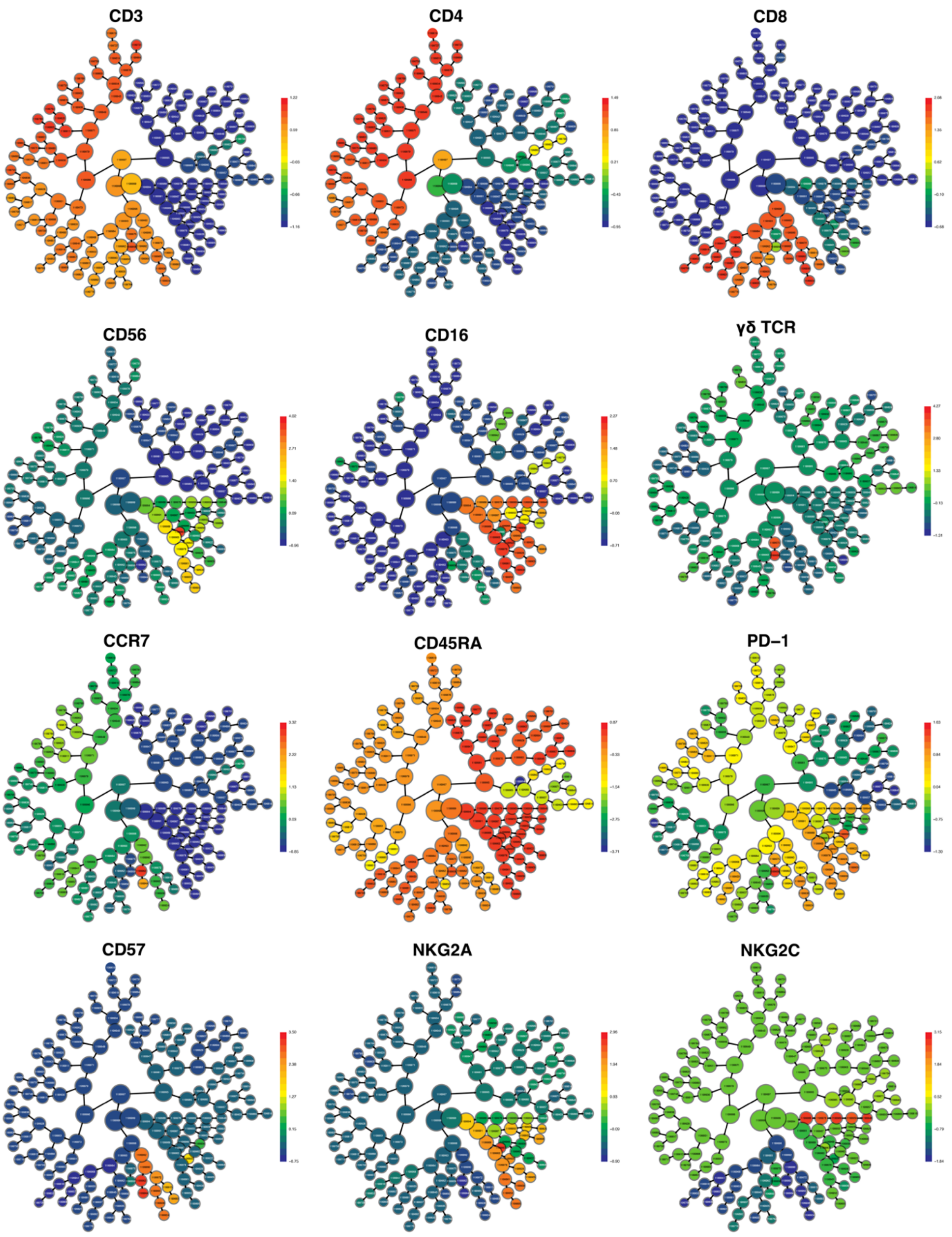

Supplementary Figure 7. CITRUS cluster map for T and NK cell phenotyping panel. Unsupervised cluster identification, characterization, and regression (CITRUS) was used to cluster immune cell populations based on CD3, CD4, CD8, CD56, CD16, γδ TCR, CCR7, CD45RA, PD-1, CD57, NKG2A, and NKG2C marker expression. CITRUS cluster map generated from 1,200,000 live, CD235a- singlet events including 75,000 events per sample (n=16 samples) with a minimum cluster size of 1%. See additional supplementary data for high resolution pdf of marker expression by cluster.

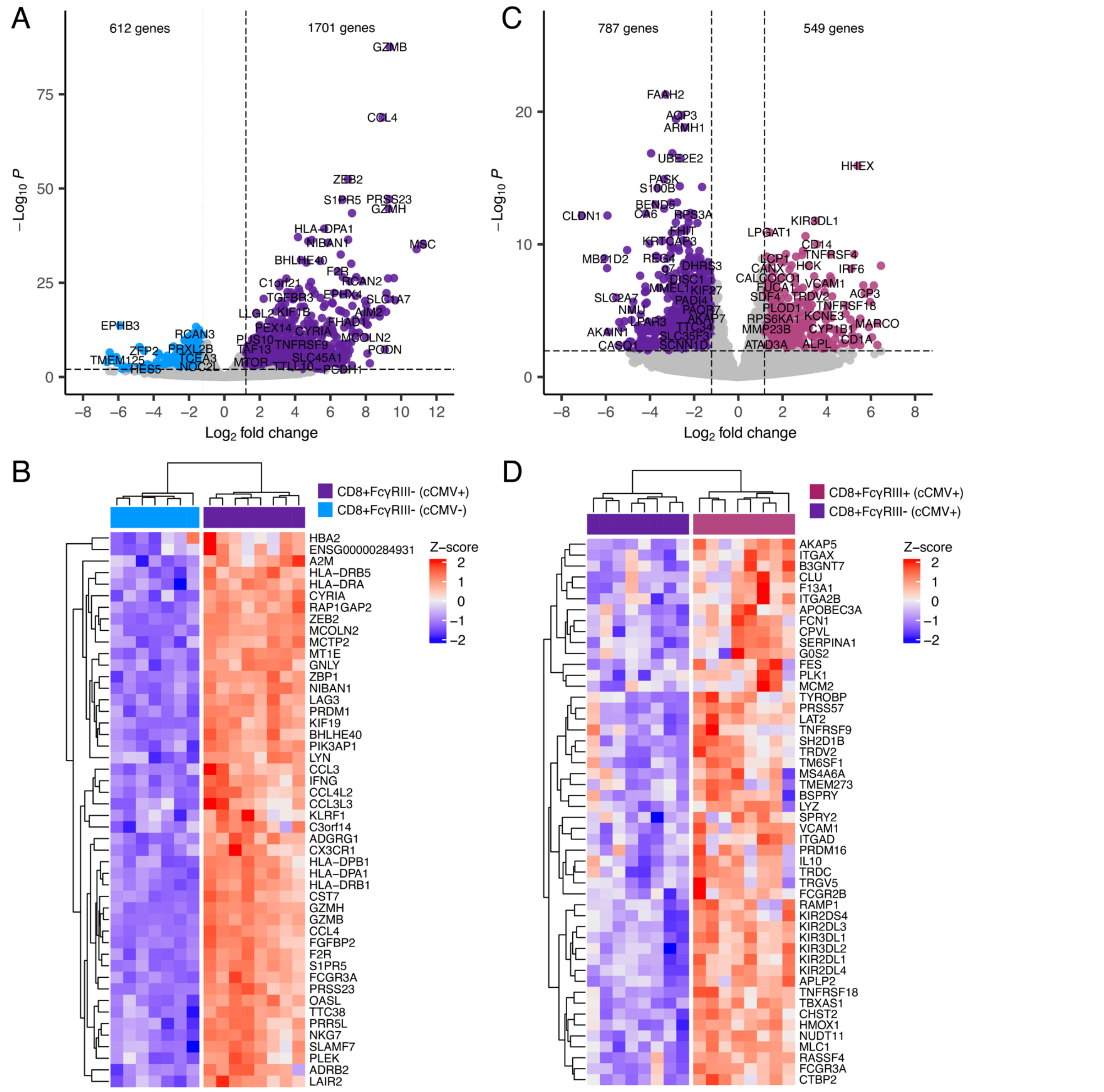

Supplementary Figure 8. Differential gene expression analysis of FcγRIII+ and FcγRIII- CD8+ T cells in cord blood from cCMV-infected and uninfected neonates. (A-E) Transcriptome analysis of FAC-sorted FcγRIII+ and FcγRIII- CD8+ T cells from cCMV-infected (n=8) and uninfected (n=7) neonates. (A) Volcano plot demonstrating differentially expressed genes ($P < 0.01$, log2foldchange +/- 1.2) in FcγRIII- CD8+ T cells from cCMV+ (dark purple) versus cCMV- (blue) neonates. (B) Heatmap showing top 48 differentially expressed genes (FDR P <0.1, log2foldchange >4.0). (C) Volcano plot of differentially expressed genes in FcγRIII+ CD8+ T cells (plum) versus FcγRIII- CD8+ T cells (dark purple) in cCMV+ neonates. (D) Heatmap showing top 50 differentially expressed genes (FDR P <0.1, log2foldchange >2.0). Z-score shows gene expression based on rlog-transformed data.

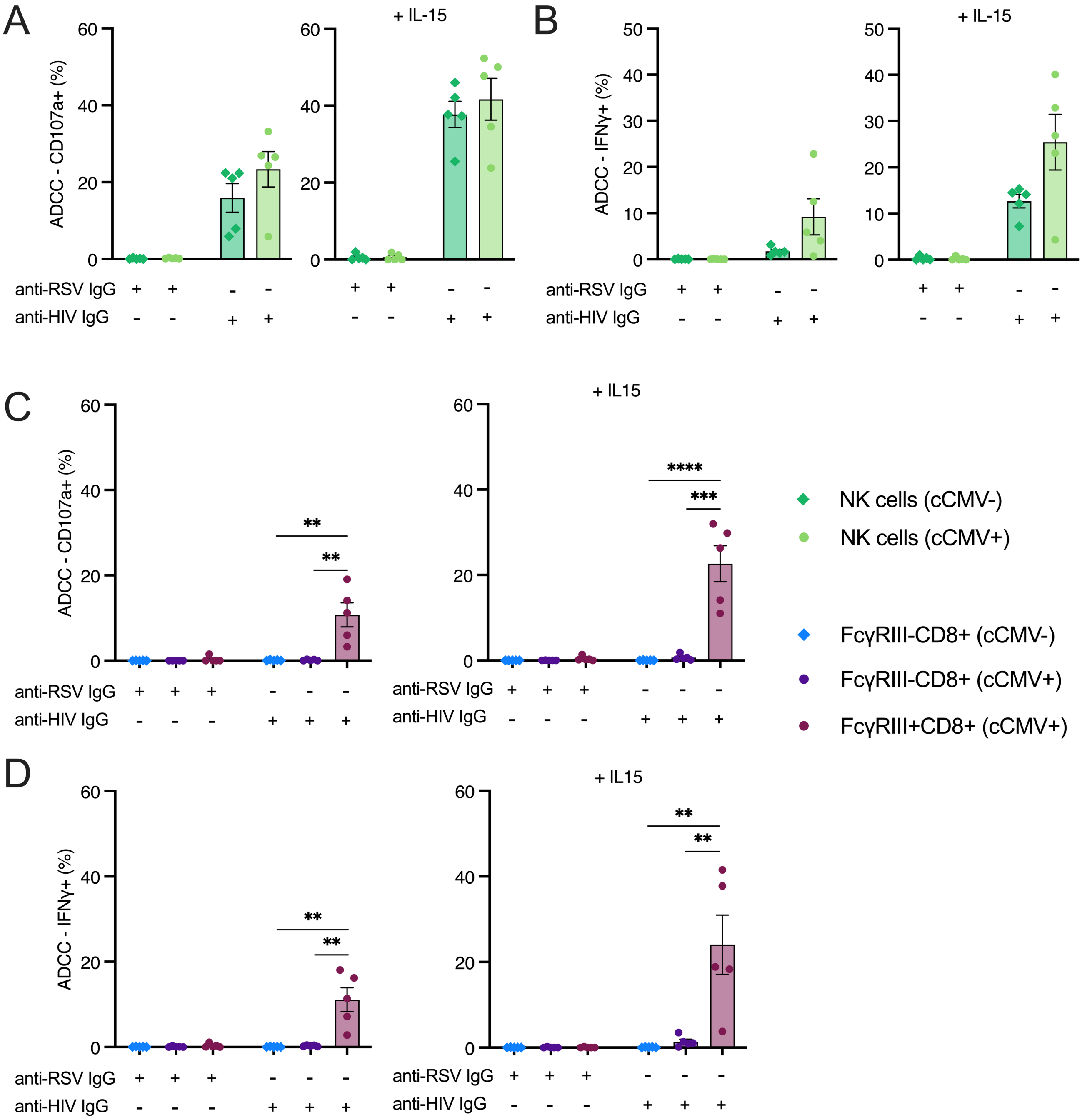

Supplementary Figure 9. NK and CD8+ T cell ADCC activity in cCMV-infected versus uninfected infants. (A-D) Degranulation (CD107a positivity) and IFNγ production following antibody stimulation with anti-RSV IgG (non-specific antibody) or anti-HIV IgG (target cell specific antibody) were measured as markers of ADCC activity in cord blood NK and CD8+ T cells from cCMV-infected (n=5 circles) and uninfected (n=5 diamonds) infants. (A-B) NK cell degranulation (CD107a positivity) and IFNγ production following antibody stimulation with and without IL-15 pretreatment in cCMV+ (light green circles) and cCMV- (dark green diamonds) infants. (C-D) CD8+ T cell degranulation (CD107a positivity) and IFNγ production following antibody stimulation with and without IL-15 pretreatment. FDR-corrected *P* values for ANOVA followed by Tukey's post hoc test. ***P* < 0.01 ****P* < 0.001 *****P* < 0.0001.

General_Lineage_CITRUS_1map

abundance

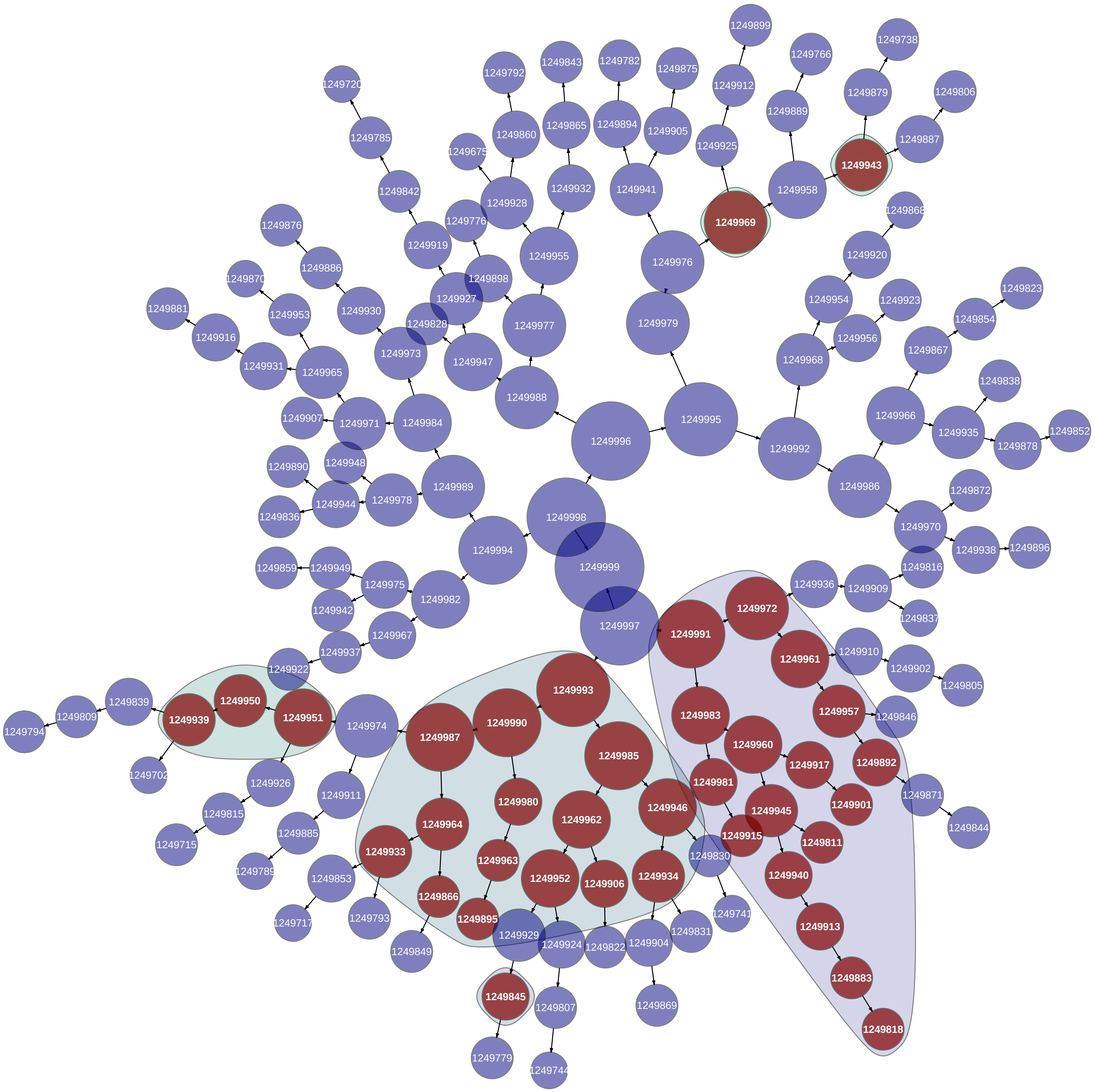
1249899
1249738
1249766
1249720
1249792
1249843
1249782
1249875
1249912
1249806
1249879
1249889
1249785
1249860
1249865
1249894
1249905
1249887
1249675
1249925
1249943
1249958
1249928
1249932
1249941
1249842
1249969
1249868
1249776
1249876
1249919
1249955
1249976
1249920
1249886
1249898
1249870
1249927
1249954
1249823
1249881
1249953
1249930
1249977
1249979
1249923
1249828
1249916
1249956
1249854
1249973
1249947
1249968
1249867
1249931
1249965
1249838
1249988
1249907
1249971
1249984
1249995
1249966
1249996
1249935
1249852
1249992
1249878
1249948
1249890
1249986
1249989
1249872
1249978
1249944
1249836
1249998
1249970
1249994
1249938
1249896
1249999
1249859
1249949
1249816
1249975
1249936
1249982
1249909
1249942
1249972
1249837
1249997
1249991
1249967
1249937
1249910
1249922
1249961
1249902
1249993
1249805
1249950
1249839
1249951
1249983
1249957
1249846
1249809
1249939
1249974
1249990
1249987
1249794
1249960
1249985
1249892
1249917
1249702
1249926
1249871
1249981
1249911
1249980
1249946
1249945
1249901
1249815
1249962
1249844
1249885
1249964
1249915
1249811
1249715
1249933
1249963
1249830
1249789
1249952
1249906
1249934
1249940
1249853
1249866
1249741
1249717
1249793
1249895
1249913
1249929
1249831
1249924
1249904
1249822
1249849
1249883
1249845
1249807
1249869
1249818
1249779
1249744

CD8

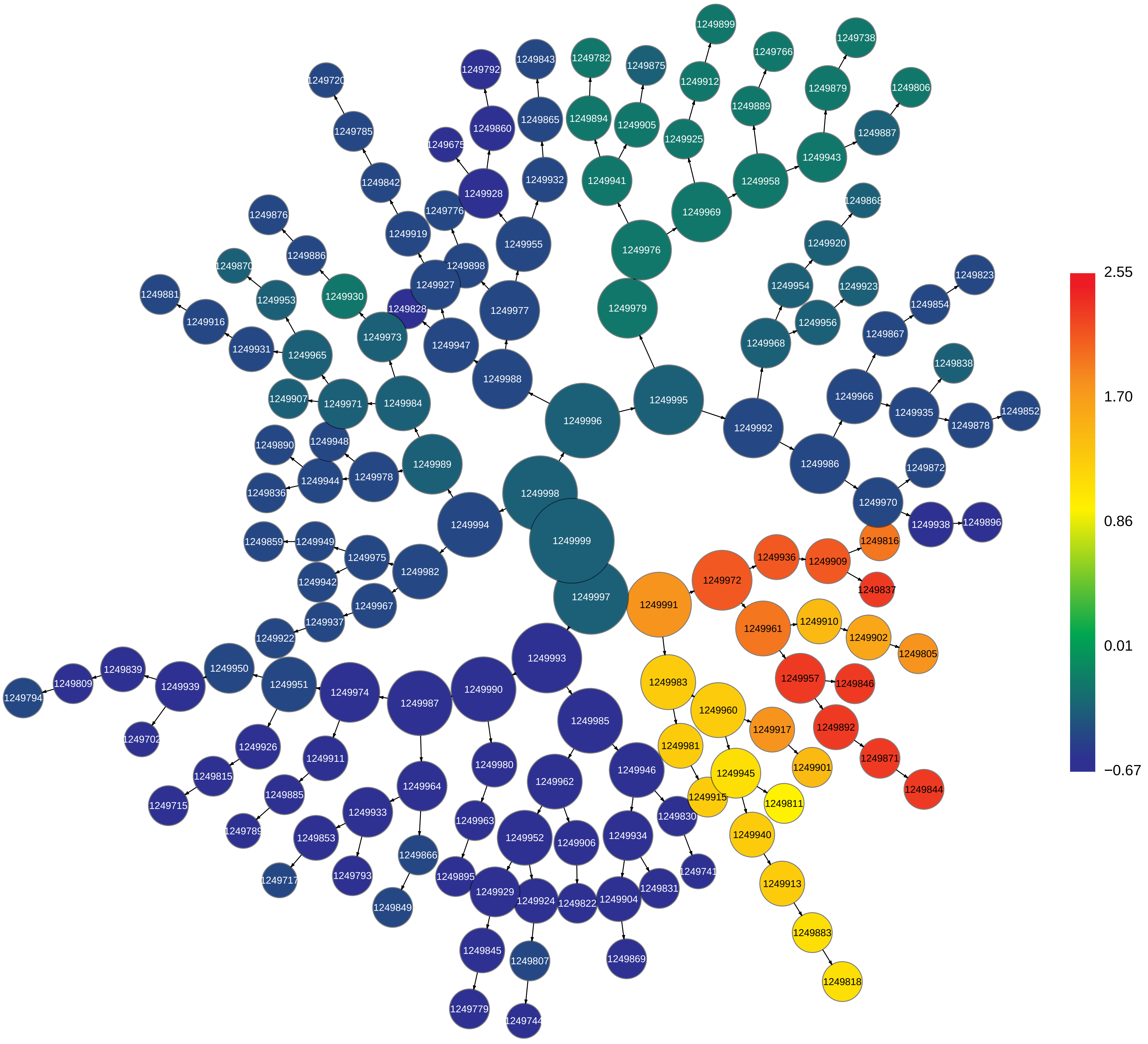
1249899
1249738
1249766
1249792
1249843
1249782
1249875
1249912
1249720
1249879
1249806
1249889
1249865
1249894
1249905
1249860
1249785
1249675
1249925
1249887
1249943
1249932
1249941
1249958
1249842
1249928
1249868
1249776
1249969
1249876
1249919
1249955
1249976
1249920
1249886
1249898
1249870
1249823
1249927
1249954
1249923
1249881
1249953
1249930
1249828
1249977
1249979
1249854
1249916
1249956
1249973
1249947
1249968
1249867
1249965
1249931
1249838
1249988
1249907
1249971
1249984
1249996
1249995
1249966
1249935
1249852
1249992
1249878
1249948
1249890
1249986
1249872
1249989
1249978
1249944
1249836
1249998
1249970
1249994
1249938
1249896
1249859
1249949
1249999
1249816
1249936
1249909
1249975
1249982
1249942
1249972
1249837
1249997
1249991
1249967
1249961
1249910
1249937
1249922
1249902
1249805
1249993
1249839
1249950
1249957
1249846
1249809
1249939
1249951
1249974
1249987
1249990
1249983
1249794
1249985
1249960
1249917
1249892
1249702
1249926
1249911
1249981
1249871
1249980
1249962
1249946
1249945
1249901
1249815
1249964
1249844
1249885
1249715
1249933
1249915
1249811
1249963
1249952
1249934
1249830
1249789
1249853
1249906
1249940
1249866
1249717
1249793
1249895
1249929
1249741
1249913
1249924
1249822
1249904
1249831
1249849
1249883
1249845
1249807
1249869
1249818
1249779
1249744
2.55
1.70
0.86
0.01
−0.67

HLA−DR

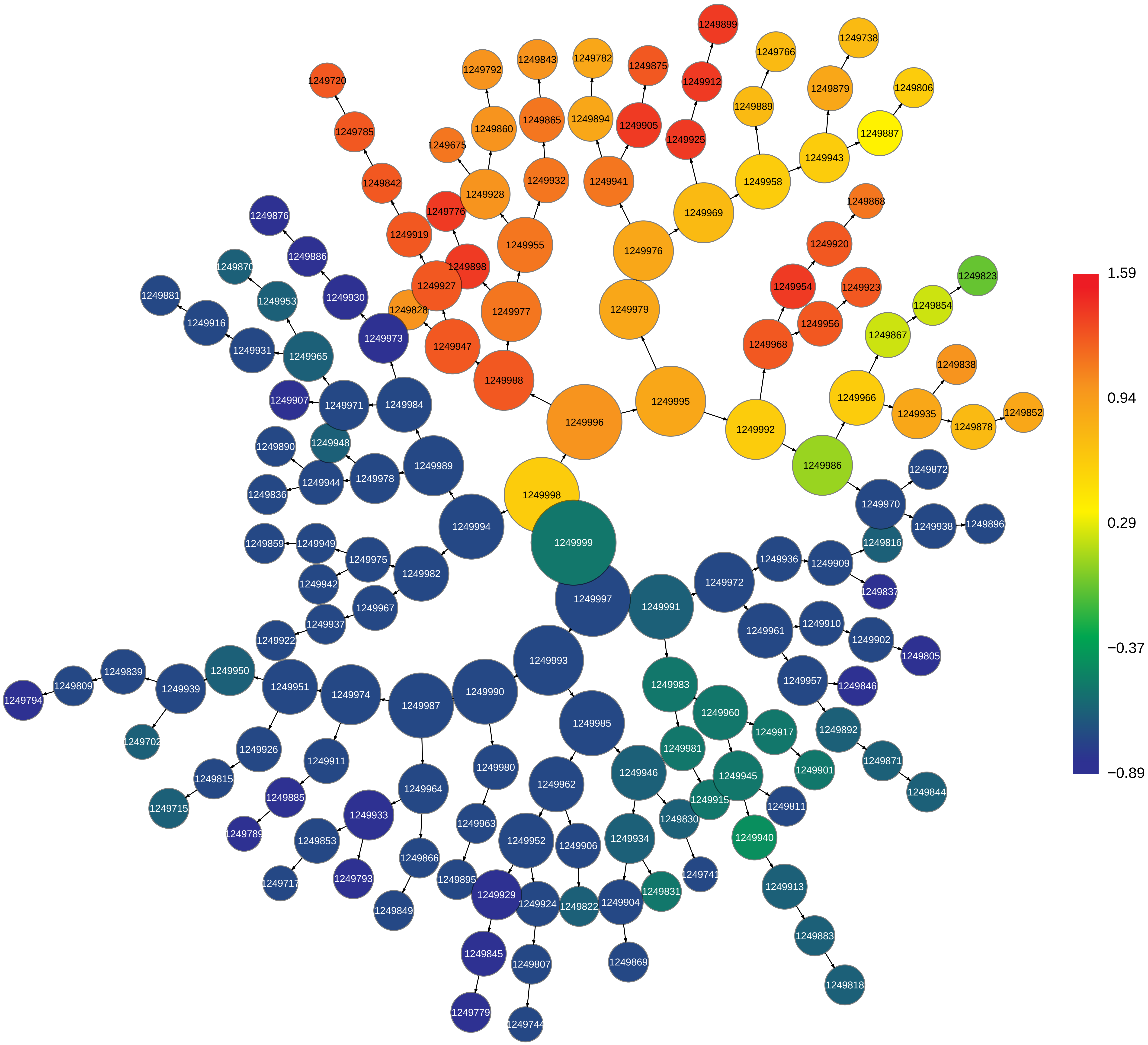

1249899
1249738
1249766
1249843
1249782
1249875
1249792
1249720
1249912
1249879
1249806
1249889
1249865
1249894
1249905
1249860
1249675
1249785
1249925
1249887
1249943
1249932
1249941
1249842
1249958
1249928
1249776
1249868
1249969
1249876
1249919
1249955
1249976
1249920
1249823
1249886
1249898
1249870
1249927
1249954
1249923
1249854
1249881
1249953
1249930
1249828
1249977
1249979
1249956
1249916
1249867
1249973
1249968
1249838
1249947
1249931
1249965
1249988
1249966
1249907
1249971
1249984
1249996
1249995
1249935
1249852
1249878
1249992
1249948
1249890
1249989
1249986
1249872
1249978
1249944
1249836
1249998
1249970
1249994
1249938
1249896
1249999
1249859
1249949
1249816
1249936
1249975
1249909
1249982
1249942
1249972
1249837
1249997
1249991
1249967
1249937
1249910
1249961
1249902
1249922
1249805
1249993
1249957
1249846
1249839
1249950
1249983
1249809
1249951
1249974
1249939
1249987
1249990
1249794
1249960
1249892
1249985
1249917
1249702
1249926
1249981
1249871
1249911
1249980
1249901
1249815
1249946
1249962
1249945
1249844
1249885
1249964
1249915
1249715
1249933
1249811
1249830
1249789
1249963
1249853
1249952
1249934
1249940
1249906
1249866
1249741
1249717
1249793
1249895
1249831
1249913
1249929
1249924
1249822
1249904
1249849
1249883
1249845
1249807
1249869
1249818
1249779
1249744
1.59
0.94
0.29
−0.37
−0.89

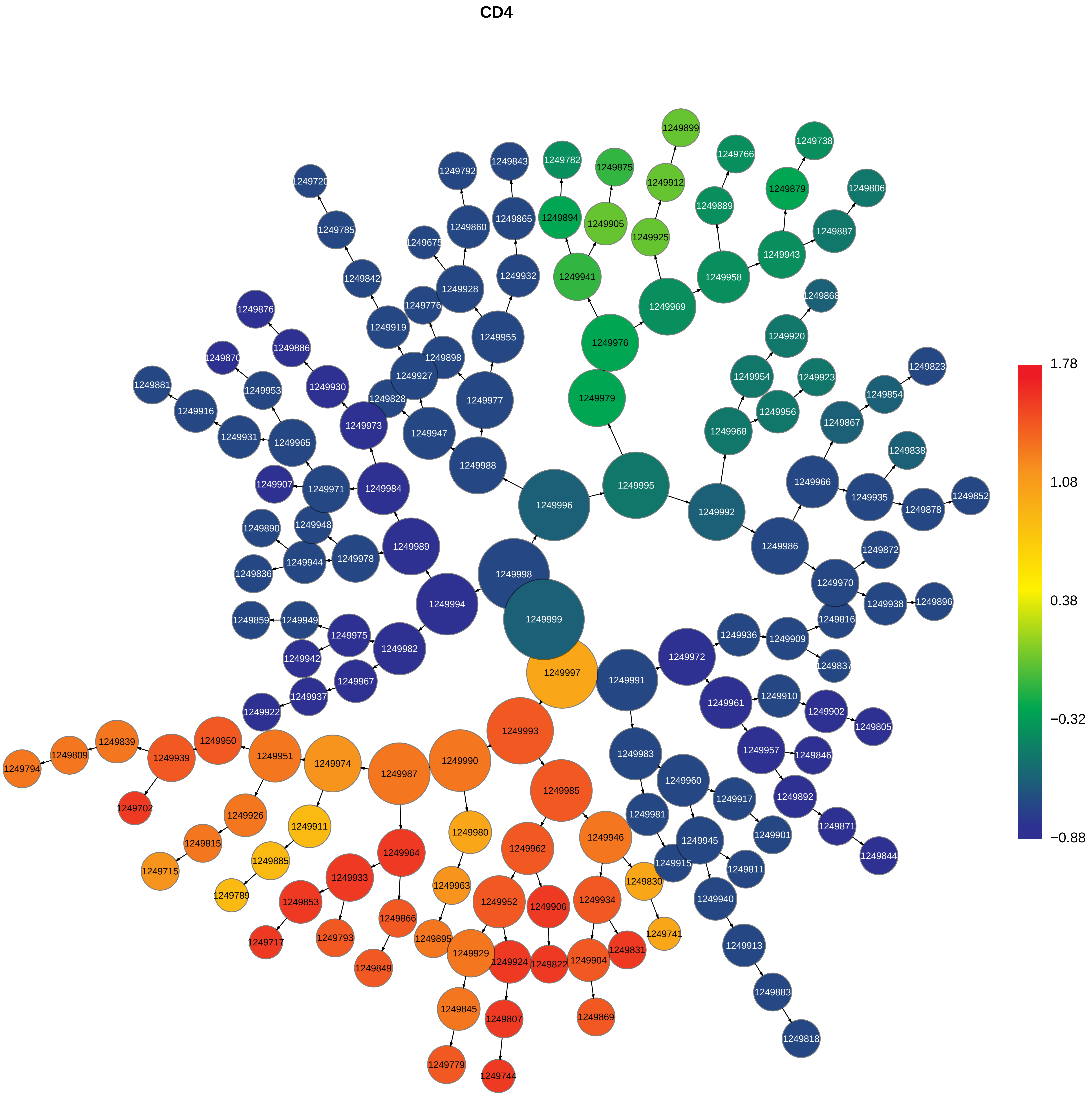
CD4
1249899
1249738
1249766
1249843
1249782
1249875
1249792
1249912
1249720
1249879
1249806
1249889
1249865
1249894
1249905
1249860
1249887
1249785
1249925
1249675
1249943
1249958
1249932
1249941
1249842
1249928
1249868
1249776
1249969
1249876
1249919
1249955
1249920
1249976
1249886
1249898
1249823
1249870
1249927
1249954
1249923
1249881
1249953
1249930
1249828
1249977
1249979
1249854
1249916
1249956
1249973
1249947
1249968
1249867
1249931
1249965
1249838
1249988
1249907
1249971
1249984
1249995
1249966
1249996
1249935
1249852
1249992
1249878
1249948
1249890
1249989
1249986
1249872
1249978
1249944
1249836
1249998
1249970
1249994
1249938
1249896
1249859
1249949
1249999
1249816
1249975
1249936
1249909
1249982
1249942
1249972
1249837
1249997
1249967
1249991
1249937
1249910
1249961
1249902
1249922
1249805
1249993
1249839
1249950
1249957
1249846
1249809
1249939
1249951
1249974
1249983
1249794
1249987
1249990
1249960
1249985
1249892
1249917
1249702
1249926
1249981
1249911
1249871
1249980
1249946
1249901
1249815
1249945
1249962
1249964
1249844
1249885
1249915
1249811
1249715
1249933
1249963
1249830
1249789
1249952
1249934
1249853
1249906
1249940
1249866
1249741
1249717
1249793
1249895
1249913
1249929
1249924
1249822
1249904
1249831
1249849
1249883
1249845
1249807
1249869
1249818
1249779
1249744
1.78
1.08
0.38
−0.32
−0.88

NKG2A

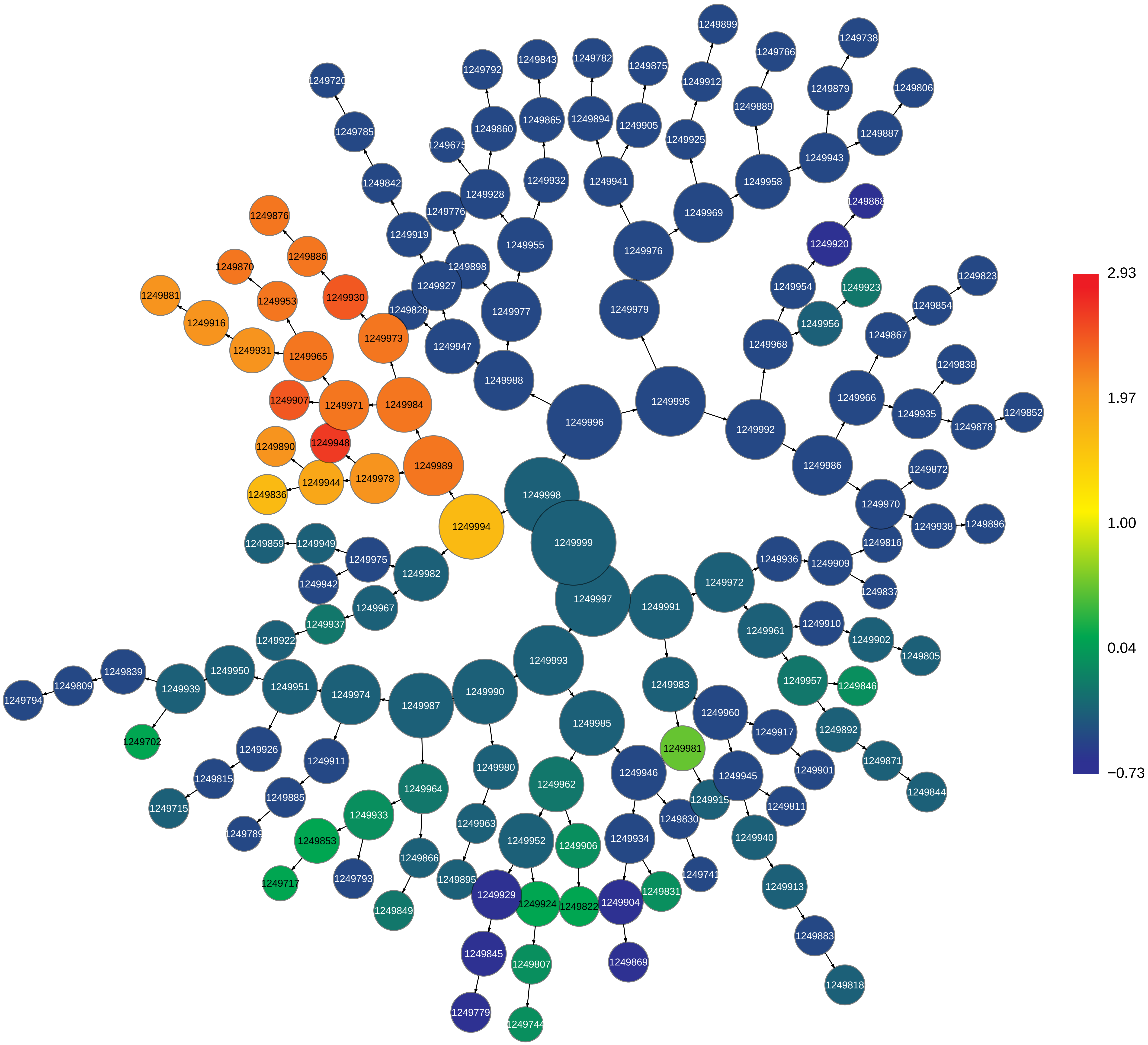
1249899
1249738
1249766
1249720
1249792
1249843
1249782
1249875
1249912
1249879
1249806
1249889
1249785
1249860
1249865
1249894
1249905
1249925
1249887
1249675
1249943
1249842
1249928
1249932
1249941
1249958
1249868
1249876
1249776
1249969
1249919
1249955
1249976
1249920
1249886
1249870
1249898
1249823
1249927
1249953
1249930
1249954
1249923
1249881
1249828
1249977
1249979
1249854
1249916
1249956
1249973
1249947
1249968
1249867
1249931
1249965
1249838
1249988
1249966
1249907
1249971
1249984
1249995
1249935
1249852
1249996
1249992
1249878
1249890
1249948
1249989
1249986
1249872
1249978
1249944
1249998
1249970
1249836
1249994
1249938
1249896
1249999
1249859
1249949
1249816
1249975
1249936
1249909
1249982
1249942
1249972
1249837
1249997
1249991
1249967
1249937
1249910
1249961
1249922
1249902
1249805
1249993
1249839
1249950
1249957
1249846
1249809
1249951
1249983
1249939
1249974
1249987
1249990
1249960
1249794
1249985
1249917
1249892
1249702
1249926
1249981
1249871
1249911
1249980
1249901
1249962
1249946
1249945
1249815
1249964
1249915
1249844
1249811
1249885
1249715
1249933
1249963
1249830
1249940
1249789
1249853
1249952
1249906
1249934
1249866
1249741
1249913
1249717
1249793
1249895
1249929
1249924
1249822
1249904
1249831
1249849
1249883
1249845
1249807
1249869
1249818
1249779
1249744
2.93
1.97
1.00
0.04
−0.73

CD127

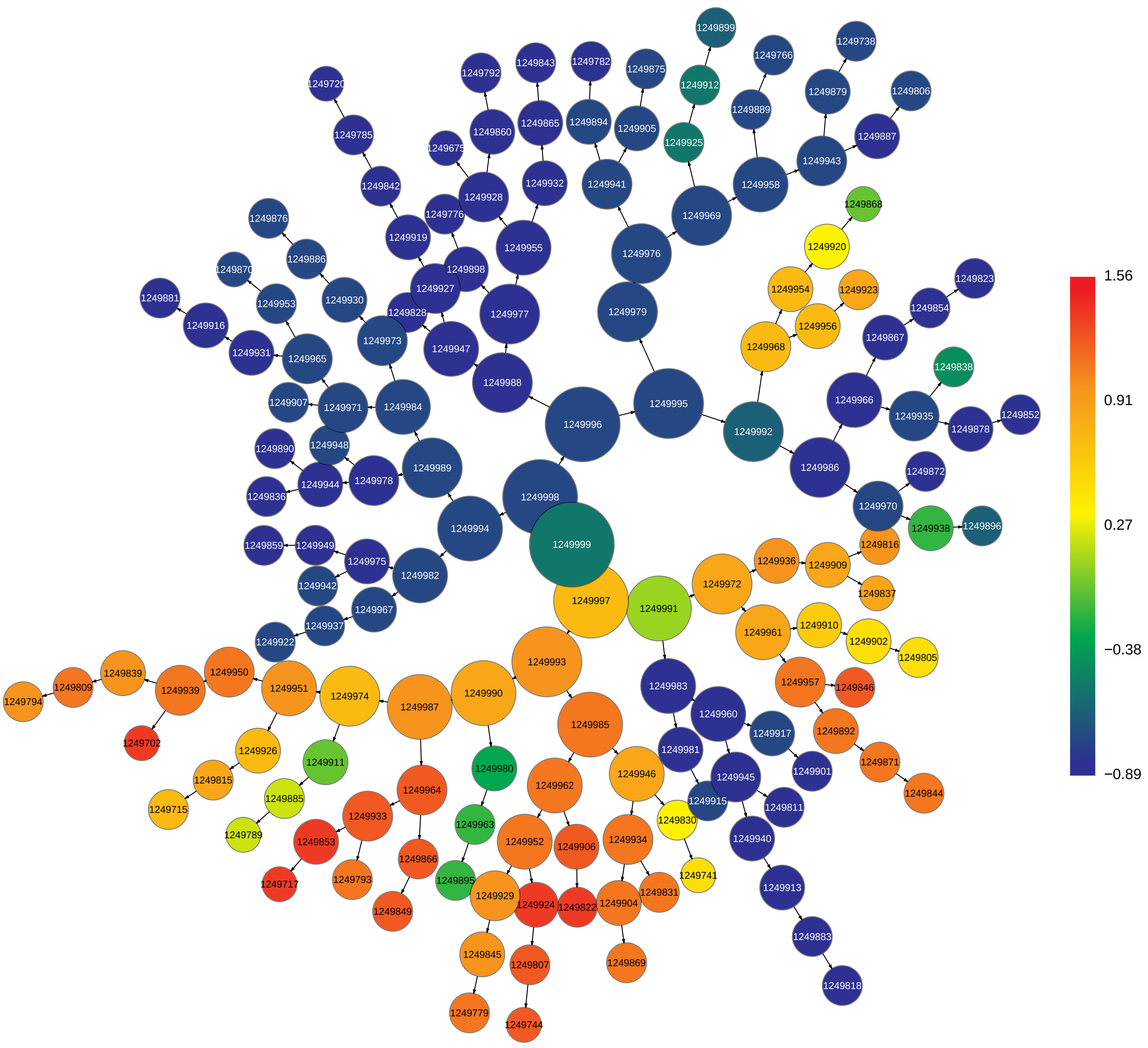
1249899
1249738
1249766
1249843
1249782
1249792
1249875
1249912
1249806
1249720
1249879
1249889
1249865
1249894
1249905
1249860
1249785
1249887
1249675
1249925
1249943
1249932
1249941
1249842
1249928
1249958
1249969
1249776
1249868
1249876
1249919
1249955
1249976
1249920
1249886
1249898
1249870
1249927
1249823
1249881
1249953
1249930
1249954
1249923
1249828
1249977
1249979
1249854
1249916
1249956
1249973
1249947
1249968
1249867
1249931
1249965
1249838
1249988
1249907
1249971
1249984
1249995
1249966
1249996
1249935
1249852
1249878
1249992
1249948
1249890
1249989
1249986
1249978
1249944
1249872
1249836
1249998
1249970
1249994
1249938
1249896
1249999
1249859
1249949
1249816
1249936
1249975
1249909
1249982
1249942
1249972
1249837
1249997
1249991
1249967
1249910
1249961
1249937
1249902
1249922
1249805
1249993
1249839
1249950
1249957
1249983
1249846
1249809
1249951
1249974
1249990
1249939
1249987
1249794
1249960
1249985
1249917
1249892
1249702
1249926
1249981
1249911
1249980
1249871
1249946
1249945
1249901
1249962
1249815
1249964
1249844
1249885
1249915
1249811
1249715
1249933
1249963
1249830
1249789
1249952
1249934
1249906
1249853
1249940
1249866
1249741
1249793
1249717
1249913
1249895
1249831
1249929
1249924
1249822
1249904
1249849
1249883
1249845
1249807
1249869
1249818
1249779
1249744
1.56
0.91
0.27
−0.38
−0.89

CD14

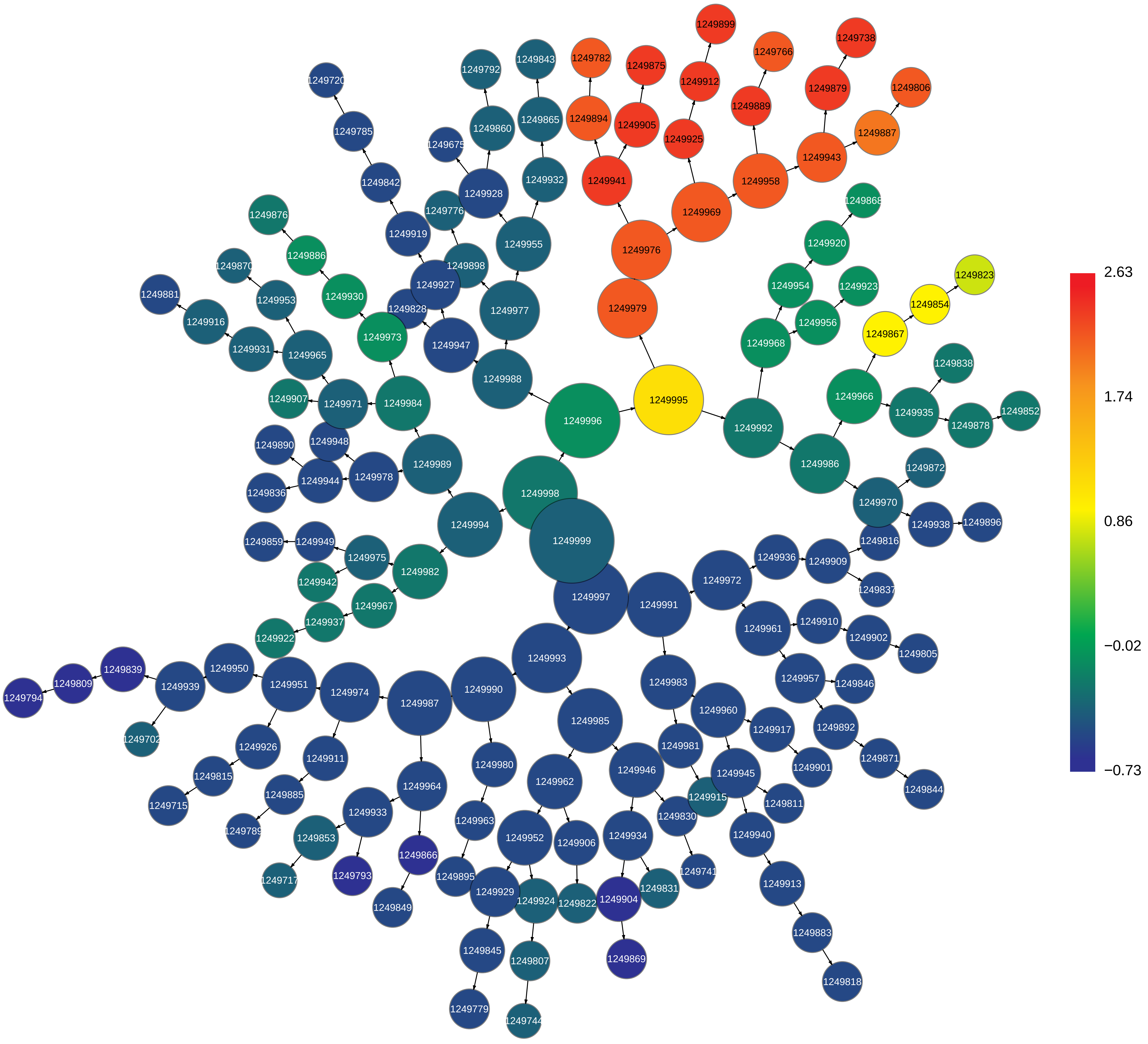
1249899
1249738
1249782
1249843
1249875
1249766
1249720
1249792
1249912
1249879
1249806
1249894
1249865
1249905
1249889
1249785
1249860
1249925
1249887
1249675
1249943
1249941
1249932
1249958
1249842
1249928
1249969
1249868
1249876
1249776
1249919
1249955
1249976
1249920
1249886
1249898
1249823
1249870
1249927
1249954
1249923
1249881
1249953
1249930
1249828
1249977
1249979
1249854
1249916
1249973
1249956
1249947
1249968
1249867
1249965
1249931
1249838
1249988
1249966
1249907
1249971
1249984
1249995
1249996
1249935
1249852
1249992
1249878
1249948
1249890
1249989
1249986
1249978
1249944
1249872
1249836
1249998
1249970
1249994
1249999
1249938
1249896
1249859
1249949
1249816
1249975
1249936
1249909
1249982
1249942
1249972
1249837
1249997
1249991
1249967
1249937
1249910
1249961
1249902
1249922
1249805
1249993
1249839
1249950
1249957
1249846
1249983
1249951
1249974
1249939
1249809
1249987
1249990
1249794
1249960
1249892
1249985
1249917
1249702
1249926
1249981
1249871
1249911
1249980
1249945
1249901
1249815
1249946
1249962
1249964
1249885
1249844
1249915
1249811
1249715
1249933
1249963
1249830
1249789
1249853
1249952
1249906
1249934
1249940
1249866
1249717
1249793
1249741
1249895
1249913
1249929
1249924
1249822
1249904
1249831
1249849
1249883
1249845
1249807
1249869
1249818
1249779
1249744
2.63
1.74
0.86
−0.02
−0.73

CD3

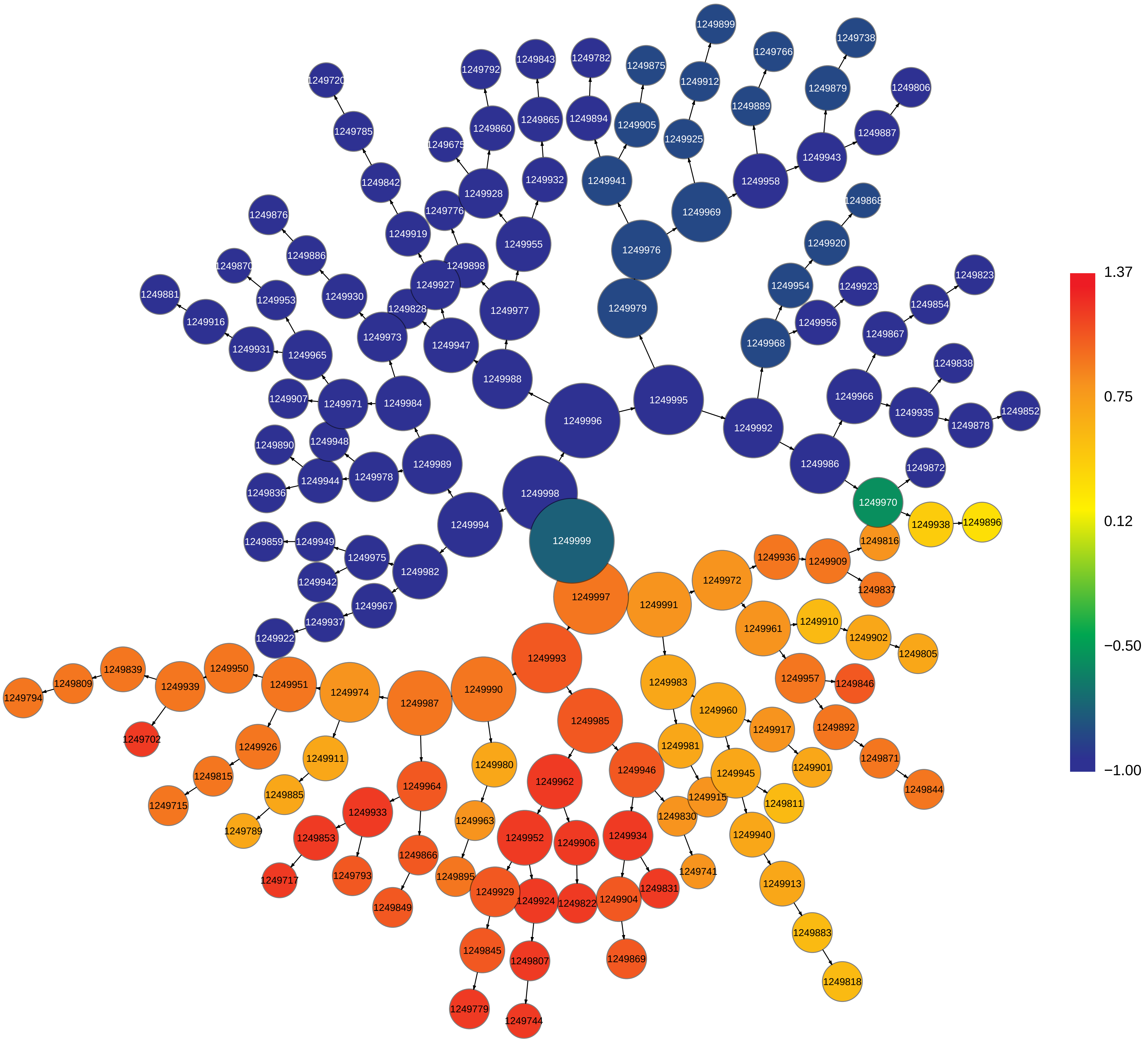
1.37
0.75
0.12
−0.50
−1.00
1249899
1249738
1249766
1249806
1249720
1249792
1249843
1249782
1249875
1249912
1249879
1249889
1249887
1249785
1249860
1249865
1249894
1249905
1249925
1249675
1249943
1249958
1249932
1249941
1249842
1249928
1249969
1249868
1249876
1249776
1249919
1249955
1249976
1249920
1249886
1249898
1249823
1249870
1249927
1249954
1249923
1249881
1249953
1249930
1249828
1249977
1249979
1249956
1249854
1249916
1249973
1249947
1249968
1249867
1249931
1249965
1249838
1249988
1249995
1249966
1249907
1249971
1249984
1249996
1249992
1249935
1249852
1249878
1249890
1249948
1249989
1249986
1249872
1249944
1249978
1249998
1249836
1249970
1249994
1249938
1249896
1249859
1249949
1249999
1249816
1249975
1249936
1249909
1249982
1249942
1249972
1249837
1249997
1249991
1249967
1249910
1249937
1249961
1249902
1249922
1249805
1249993
1249839
1249950
1249957
1249846
1249809
1249939
1249951
1249974
1249983
1249794
1249987
1249990
1249960
1249985
1249917
1249892
1249702
1249926
1249981
1249911
1249980
1249871
1249815
1249945
1249901
1249962
1249946
1249964
1249844
1249885
1249715
1249915
1249811
1249933
1249830
1249963
1249952
1249934
1249853
1249906
1249940
1249789
1249741
1249866
1249913
1249717
1249793
1249895
1249929
1249924
1249822
1249904
1249831
1249849
1249883
1249845
1249807
1249869
1249818
1249779
1249744

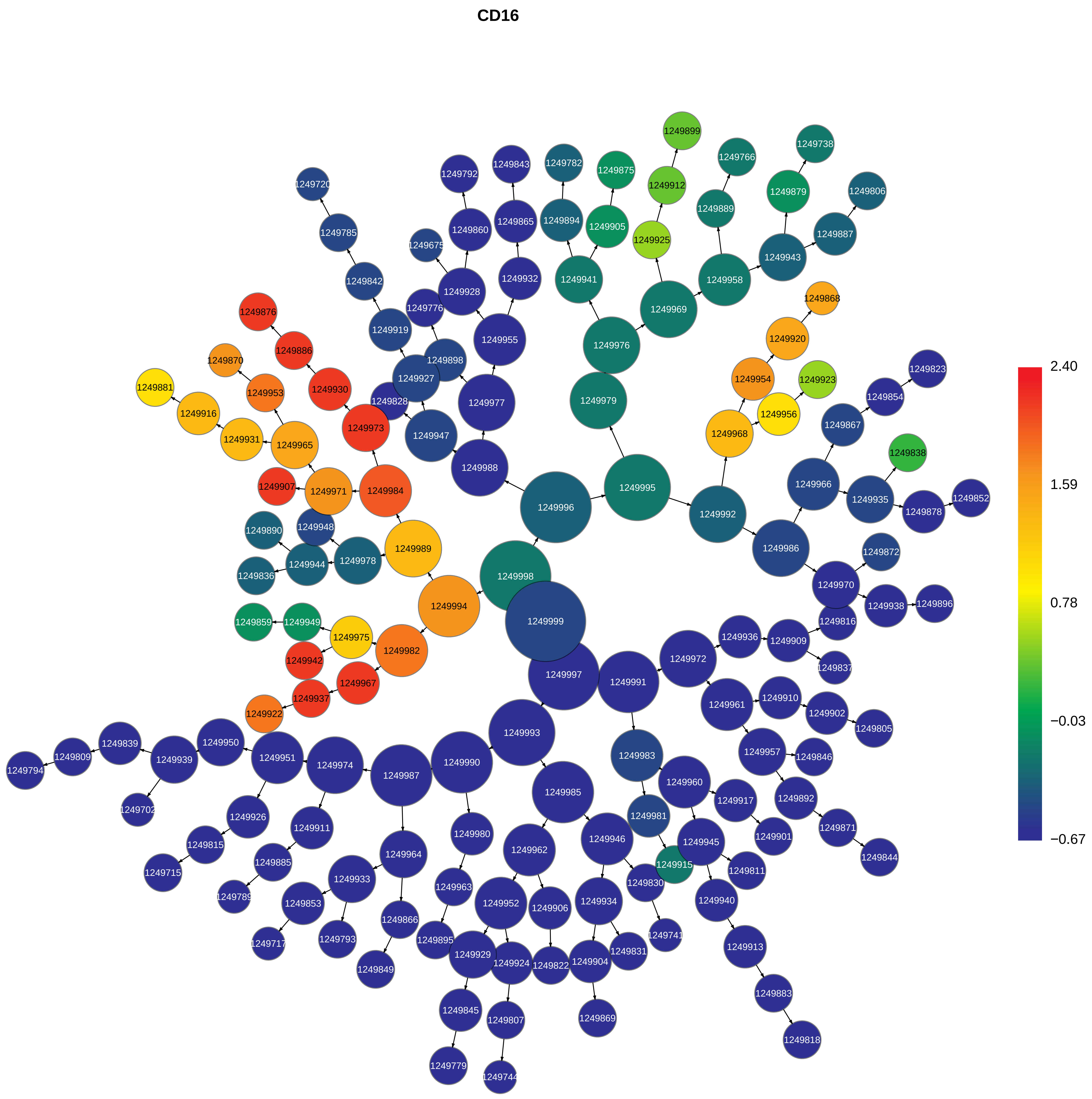
CD16
2.40
1.59
0.78
−0.03
−0.67
1249899
1249738
1249843
1249782
1249875
1249766
1249792
1249720
1249912
1249879
1249806
1249865
1249894
1249905
1249889
1249860
1249785
1249675
1249925
1249887
1249943
1249958
1249932
1249941
1249842
1249928
1249876
1249868
1249776
1249969
1249919
1249955
1249976
1249886
1249920
1249898
1249870
1249823
1249927
1249954
1249923
1249953
1249881
1249930
1249828
1249977
1249979
1249854
1249916
1249956
1249973
1249968
1249867
1249947
1249931
1249965
1249838
1249988
1249907
1249971
1249984
1249995
1249966
1249996
1249935
1249852
1249992
1249878
1249948
1249890
1249989
1249986
1249978
1249944
1249872
1249836
1249998
1249970
1249994
1249938
1249896
1249859
1249949
1249999
1249816
1249975
1249936
1249909
1249982
1249942
1249972
1249837
1249997
1249991
1249967
1249937
1249910
1249961
1249902
1249922
1249805
1249993
1249839
1249950
1249957
1249846
1249809
1249939
1249951
1249974
1249983
1249987
1249990
1249794
1249960
1249985
1249917
1249892
1249702
1249926
1249981
1249911
1249980
1249871
1249962
1249946
1249945
1249901
1249815
1249964
1249915
1249811
1249844
1249885
1249715
1249933
1249963
1249830
1249789
1249952
1249934
1249940
1249853
1249906
1249866
1249717
1249793
1249895
1249741
1249913
1249929
1249831
1249924
1249904
1249822
1249849
1249883
1249845
1249807
1249869
1249818
1249779
1249744

CD25

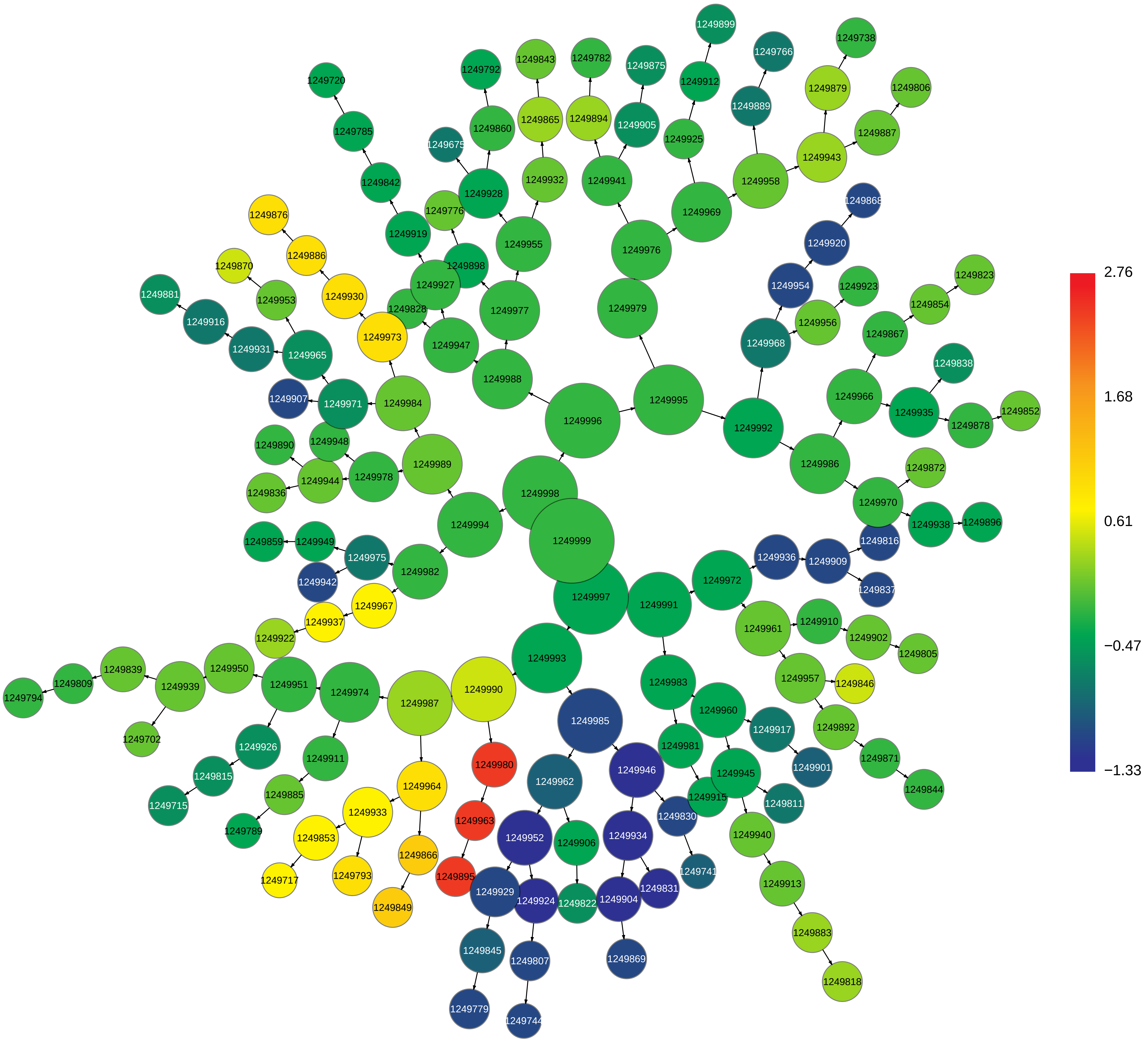

2.76
1.68
0.61
−0.47
−1.33
1249899
1249738
1249766
1249720
1249792
1249843
1249782
1249875
1249912
1249879
1249806
1249785
1249860
1249865
1249894
1249905
1249889
1249887
1249675
1249925
1249943
1249932
1249941
1249958
1249842
1249928
1249868
1249876
1249776
1249969
1249919
1249955
1249976
1249920
1249886
1249898
1249870
1249927
1249954
1249923
1249823
1249881
1249953
1249930
1249828
1249977
1249979
1249854
1249916
1249956
1249973
1249947
1249968
1249867
1249931
1249965
1249838
1249988
1249907
1249971
1249984
1249966
1249995
1249935
1249852
1249996
1249992
1249878
1249948
1249890
1249989
1249978
1249944
1249986
1249872
1249836
1249998
1249970
1249994
1249938
1249896
1249999
1249859
1249949
1249816
1249975
1249936
1249909
1249982
1249942
1249972
1249837
1249997
1249991
1249967
1249937
1249910
1249961
1249902
1249922
1249805
1249993
1249839
1249950
1249983
1249957
1249846
1249809
1249939
1249951
1249974
1249990
1249794
1249987
1249985
1249960
1249892
1249917
1249702
1249926
1249981
1249871
1249911
1249980
1249901
1249946
1249844
1249815
1249962
1249945
1249964
1249915
1249885
1249811
1249715
1249933
1249963
1249830
1249789
1249934
1249952
1249906
1249940
1249853
1249866
1249741
1249913
1249717
1249793
1249895
1249929
1249924
1249822
1249904
1249831
1249849
1249883
1249845
1249807
1249869
1249818
1249779
1249744

CD56

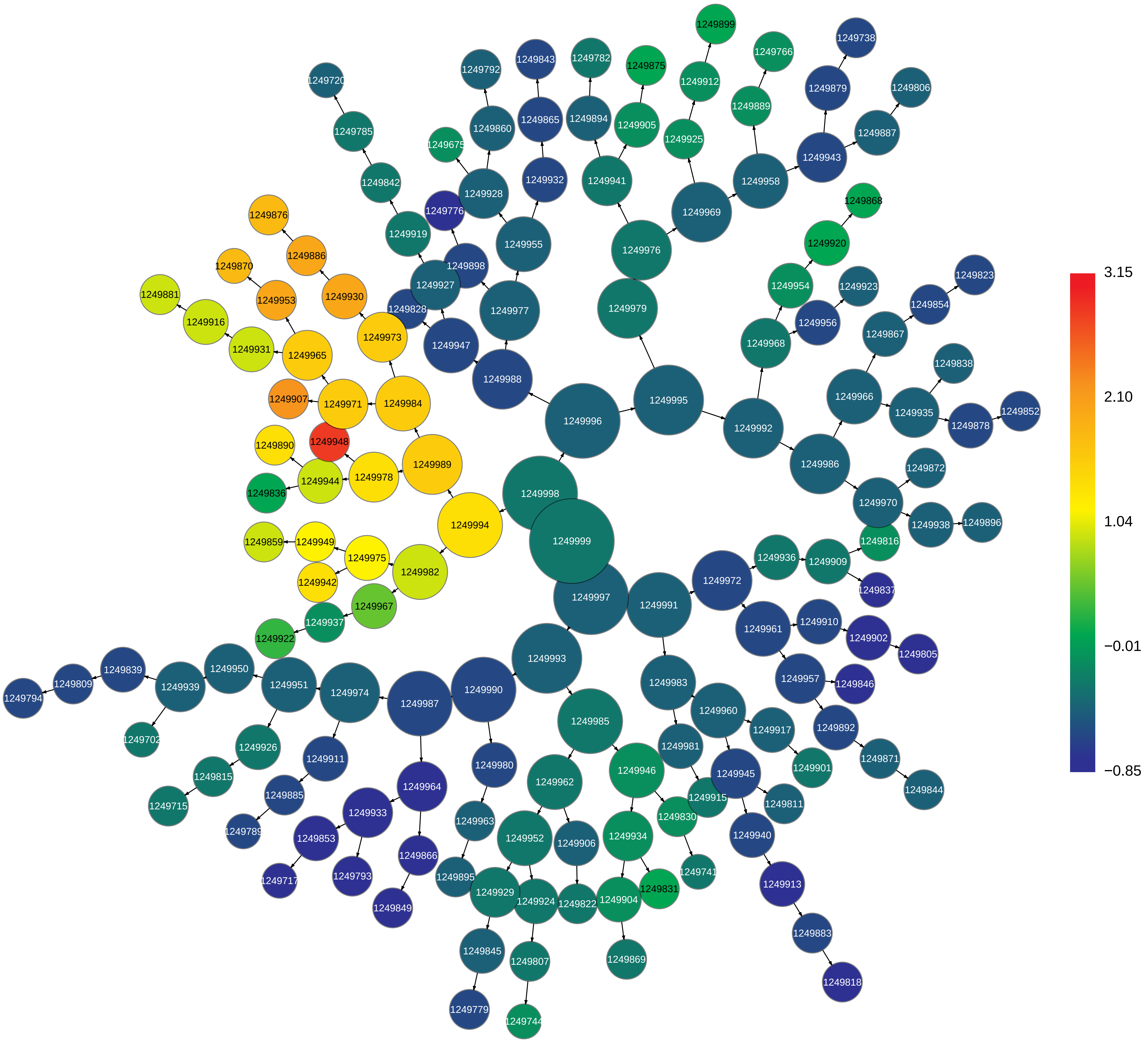

3.15
2.10
1.04
−0.01
−0.85
1249899
1249738
1249766
1249843
1249782
1249875
1249792
1249720
1249912
1249879
1249806
1249889
1249865
1249894
1249905
1249860
1249785
1249675
1249925
1249887
1249943
1249941
1249932
1249958
1249842
1249928
1249776
1249868
1249969
1249876
1249919
1249955
1249976
1249920
1249886
1249898
1249870
1249823
1249954
1249923
1249927
1249930
1249953
1249881
1249828
1249977
1249979
1249854
1249916
1249956
1249973
1249968
1249867
1249947
1249931
1249965
1249838
1249988
1249966
1249995
1249852
1249907
1249971
1249984
1249935
1249996
1249992
1249878
1249948
1249890
1249986
1249872
1249989
1249978
1249944
1249998
1249836
1249970
1249896
1249938
1249994
1249999
1249816
1249859
1249949
1249975
1249936
1249909
1249982
1249942
1249972
1249837
1249997
1249967
1249991
1249937
1249910
1249922
1249961
1249902
1249805
1249993
1249839
1249950
1249957
1249846
1249809
1249951
1249939
1249974
1249987
1249990
1249983
1249794
1249985
1249960
1249892
1249917
1249702
1249926
1249981
1249871
1249911
1249980
1249946
1249945
1249901
1249815
1249964
1249962
1249844
1249885
1249915
1249811
1249715
1249933
1249830
1249963
1249934
1249789
1249952
1249906
1249940
1249853
1249866
1249741
1249913
1249717
1249793
1249895
1249929
1249924
1249822
1249904
1249831
1249849
1249883
1249845
1249807
1249869
1249818
1249779
1249744

NKG2C

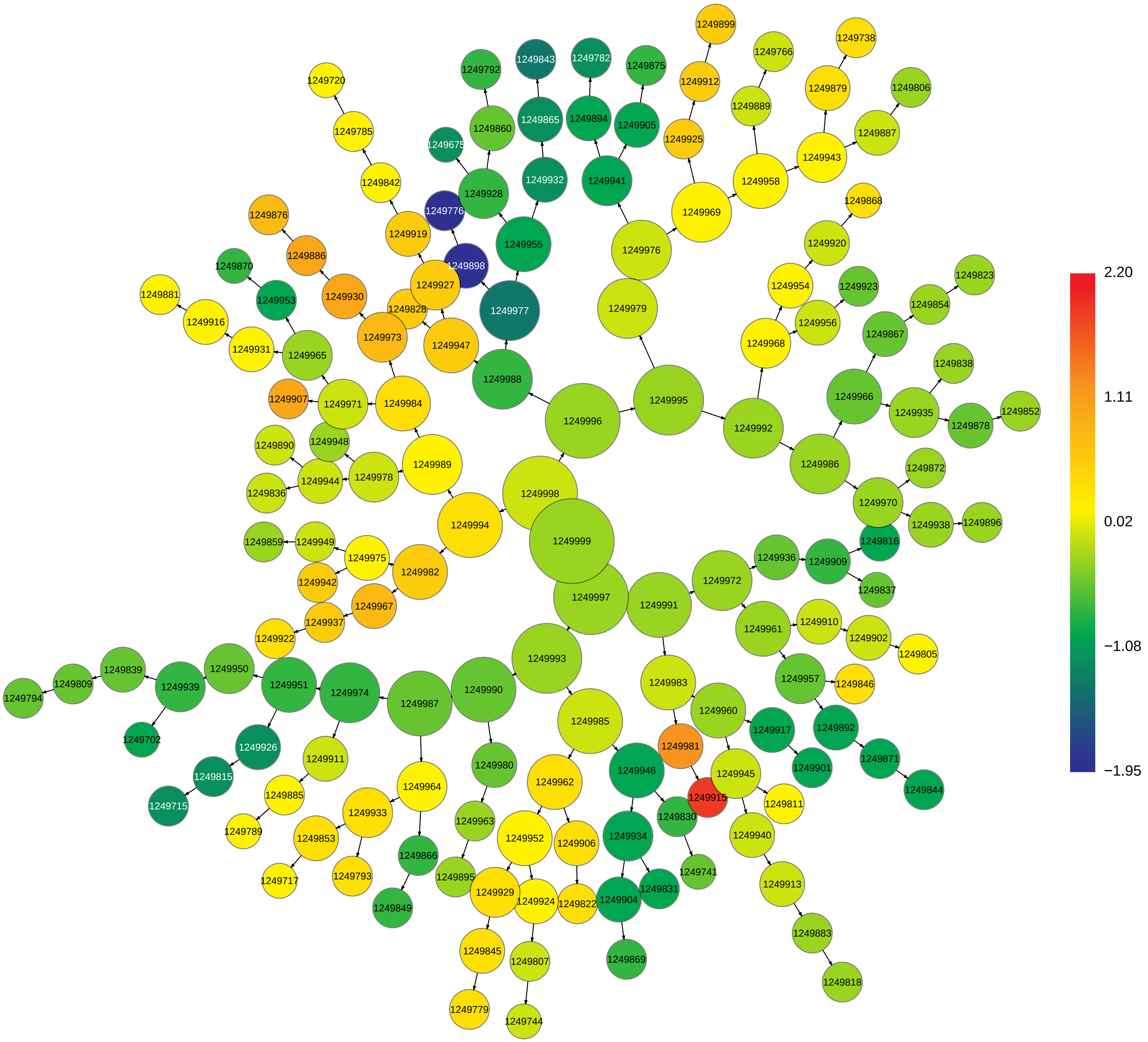
1249899
1249738
1249766
1249843
1249782
1249875
1249792
1249912
1249720
1249879
1249806
1249889
1249865
1249894
1249905
1249860
1249785
1249887
1249925
1249675
1249943
1249932
1249941
1249958
1249842
1249928
1249868
1249776
1249969
1249876
1249919
1249955
1249976
1249920
1249886
1249898
1249870
1249823
1249927
1249954
1249923
1249930
1249953
1249881
1249828
1249977
1249979
1249854
1249916
1249956
1249973
1249947
1249968
1249867
1249931
1249965
1249838
1249988
1249907
1249971
1249984
1249966
1249995
1249852
1249935
1249996
1249878
1249992
1249948
1249890
1249989
1249986
1249872
1249978
1249944
1249836
1249998
1249970
1249994
1249938
1249896
1249859
1249949
1249999
1249816
1249975
1249936
1249909
1249982
1249942
1249972
1249837
1249997
1249967
1249991
1249937
1249910
1249961
1249902
1249922
1249805
1249993
1249839
1249950
1249983
1249957
1249846
1249809
1249951
1249939
1249974
1249987
1249990
1249985
1249960
1249794
1249892
1249917
1249702
1249926
1249981
1249871
1249911
1249980
1249946
1249901
1249815
1249962
1249945
1249964
1249844
1249885
1249915
1249811
1249715
1249933
1249963
1249830
1249789
1249853
1249952
1249934
1249906
1249940
1249866
1249717
1249793
1249895
1249741
1249913
1249929
1249924
1249831
1249822
1249904
1249849
1249883
1249845
1249807
1249869
1249818
1249779
1249744
2.20
1.11
0.02
−1.08
−1.95

CD19

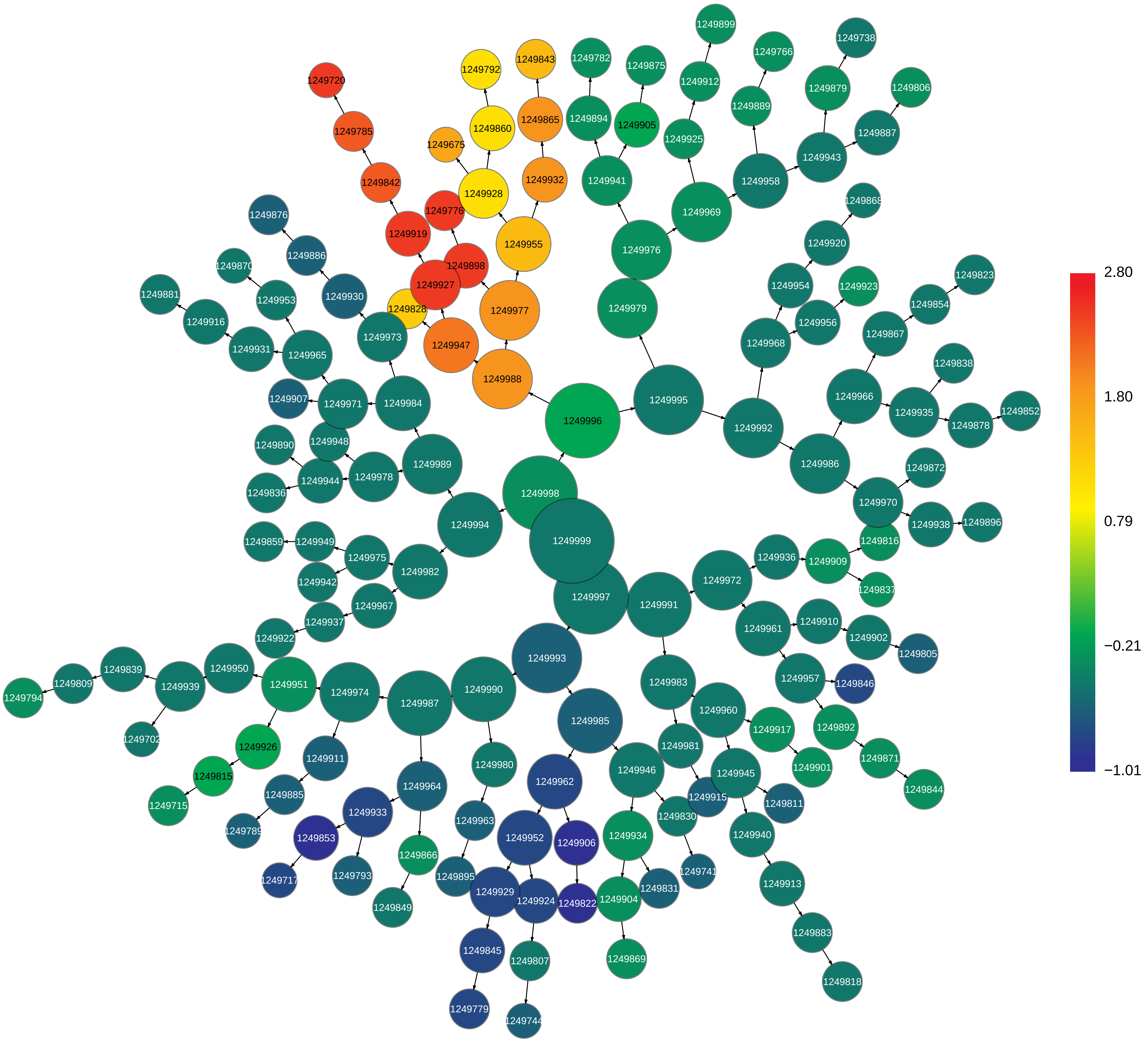
1249899
1249738
1249843
1249792
1249782
1249875
1249766
1249720
1249912
1249879
1249806
1249889
1249865
1249894
1249905
1249785
1249860
1249675
1249925
1249887
1249943
1249932
1249941
1249958
1249842
1249928
1249776
1249868
1249876
1249969
1249919
1249955
1249976
1249920
1249886
1249898
1249870
1249927
1249823
1249881
1249953
1249930
1249828
1249954
1249923
1249977
1249979
1249854
1249916
1249956
1249973
1249947
1249968
1249867
1249931
1249965
1249838
1249988
1249907
1249971
1249984
1249995
1249966
1249935
1249852
1249996
1249878
1249992
1249948
1249890
1249989
1249986
1249872
1249978
1249944
1249836
1249998
1249970
1249896
1249994
1249938
1249999
1249859
1249949
1249816
1249975
1249936
1249909
1249982
1249942
1249972
1249837
1249997
1249967
1249991
1249910
1249937
1249961
1249902
1249922
1249805
1249993
1249957
1249839
1249950
1249846
1249983
1249809
1249951
1249939
1249974
1249794
1249990
1249987
1249960
1249985
1249892
1249917
1249702
1249926
1249981
1249871
1249911
1249980
1249946
1249945
1249901
1249815
1249962
1249844
1249964
1249915
1249811
1249885
1249933
1249715
1249830
1249963
1249934
1249940
1249789
1249853
1249952
1249906
1249866
1249741
1249717
1249793
1249895
1249913
1249929
1249831
1249924
1249822
1249904
1249849
1249883
1249845
1249807
1249869
1249818
1249779
1249744
2.80
1.80
0.79
−0.21
−1.01

General_Lineage_CITRUS_2clusters

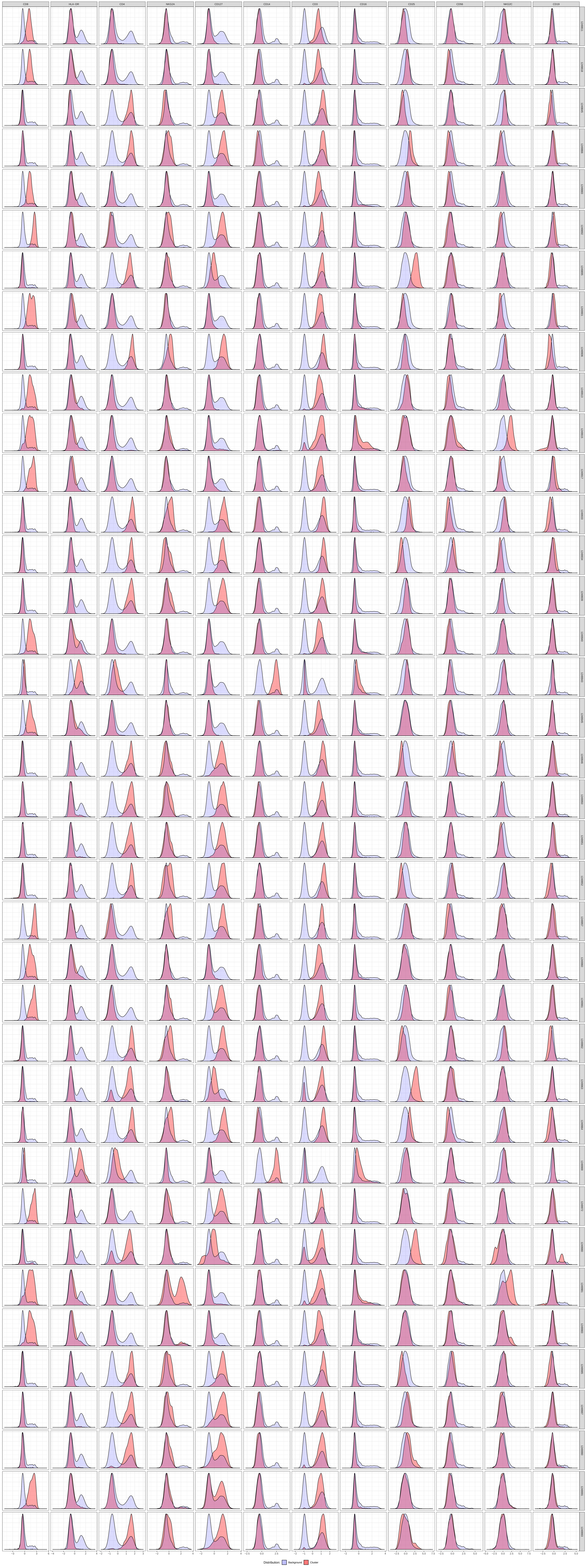

CD8
HLA−DR
CD4
NKG2A
CD127
CD14
CD3
CD16
CD25
CD56
NKG2C
CD19
Distribution:
Background
Cluster

General_Lineage_CITRUS_3plots

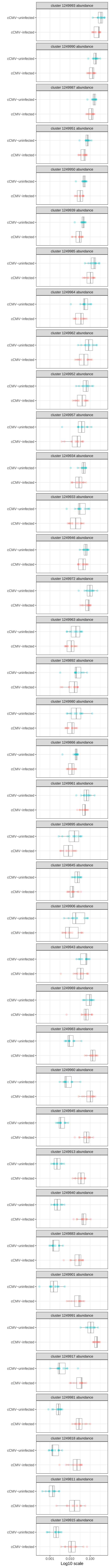
cluster 1249993 abundance
cluster 1249990 abundance
cluster 1249987 abundance
cluster 1249951 abundance
cluster 1249950 abundance
cluster 1249939 abundance
cluster 1249985 abundance
cluster 1249964 abundance
cluster 1249962 abundance
cluster 1249952 abundance
cluster 1249957 abundance
cluster 1249934 abundance
cluster 1249933 abundance
cluster 1249946 abundance
cluster 1249972 abundance
cluster 1249963 abundance
cluster 1249892 abundance
cluster 1249980 abundance
cluster 1249866 abundance
cluster 1249961 abundance
cluster 1249895 abundance
cluster 1249845 abundance
cluster 1249906 abundance
cluster 1249943 abundance
cluster 1249969 abundance
cluster 1249983 abundance
cluster 1249960 abundance
cluster 1249945 abundance
cluster 1249913 abundance
cluster 1249940 abundance
cluster 1249883 abundance
cluster 1249901 abundance
cluster 1249991 abundance
cluster 1249917 abundance
cluster 1249981 abundance
cluster 1249818 abundance
cluster 1249811 abundance
cluster 1249915 abundance
cCMV−uninfected
cCMV−infected
0.001
0.010
0.100
Log10 scale

NK_T_CITRUS_1map

abundance

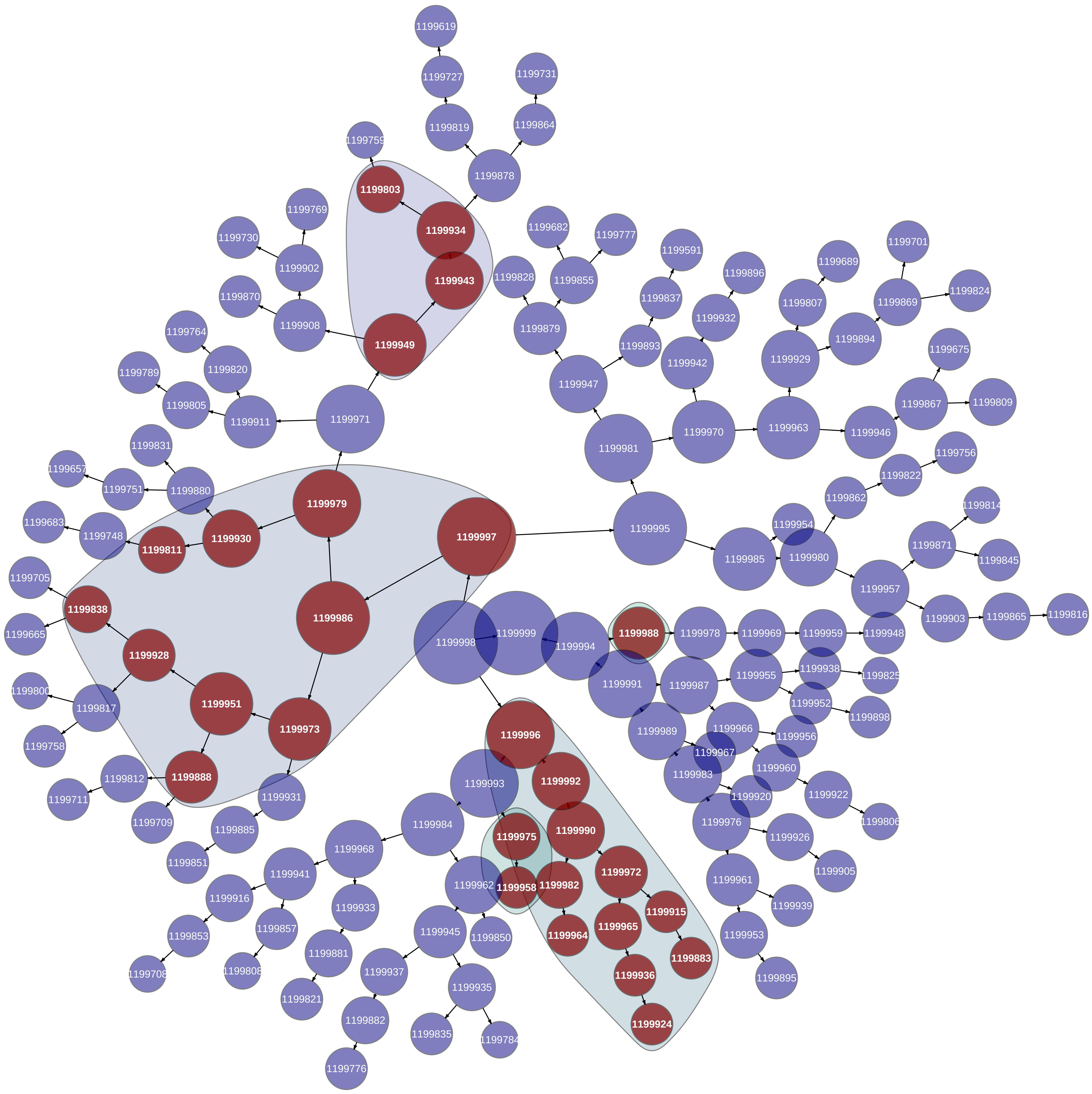
1199619
1199727
1199731
1199864
1199819
1199759
1199878
1199803
1199934
1199769
1199730
1199902
1199682
1199777
1199591
1199896
1199689
1199701
1199943
1199828
1199855
1199837
1199932
1199807
1199869
1199824
1199870
1199908
1199949
1199879
1199893
1199942
1199929
1199894
1199764
1199820
1199789
1199947
1199675
1199805
1199911
1199971
1199970
1199963
1199867
1199809
1199946
1199981
1199831
1199756
1199657
1199880
1199751
1199822
1199979
1199862
1199814
1199683
1199748
1199930
1199811
1199997
1199995
1199954
1199871
1199985
1199980
1199845
1199705
1199957
1199838
1199986
1199903
1199865
1199816
1199998
1199999
1199994
1199988
1199978
1199969
1199959
1199948
1199665
1199928
1199991
1199987
1199955
1199938
1199825
1199800
1199951
1199817
1199952
1199898
1199973
1199996
1199989
1199966
1199956
1199758
1199967
1199812
1199888
1199992
1199983
1199960
1199711
1199931
1199993
1199920
1199922
1199709
1199885
1199984
1199976
1199806
1199851
1199968
1199975
1199990
1199926
1199941
1199905
1199916
1199962
1199958
1199982
1199972
1199961
1199933
1199939
1199857
1199945
1199915
1199853
1199850
1199964
1199965
1199953
1199881
1199883
1199808
1199708
1199937
1199936
1199895
1199935
1199821
1199882
1199924
1199835
1199784
1199776

TCR gd

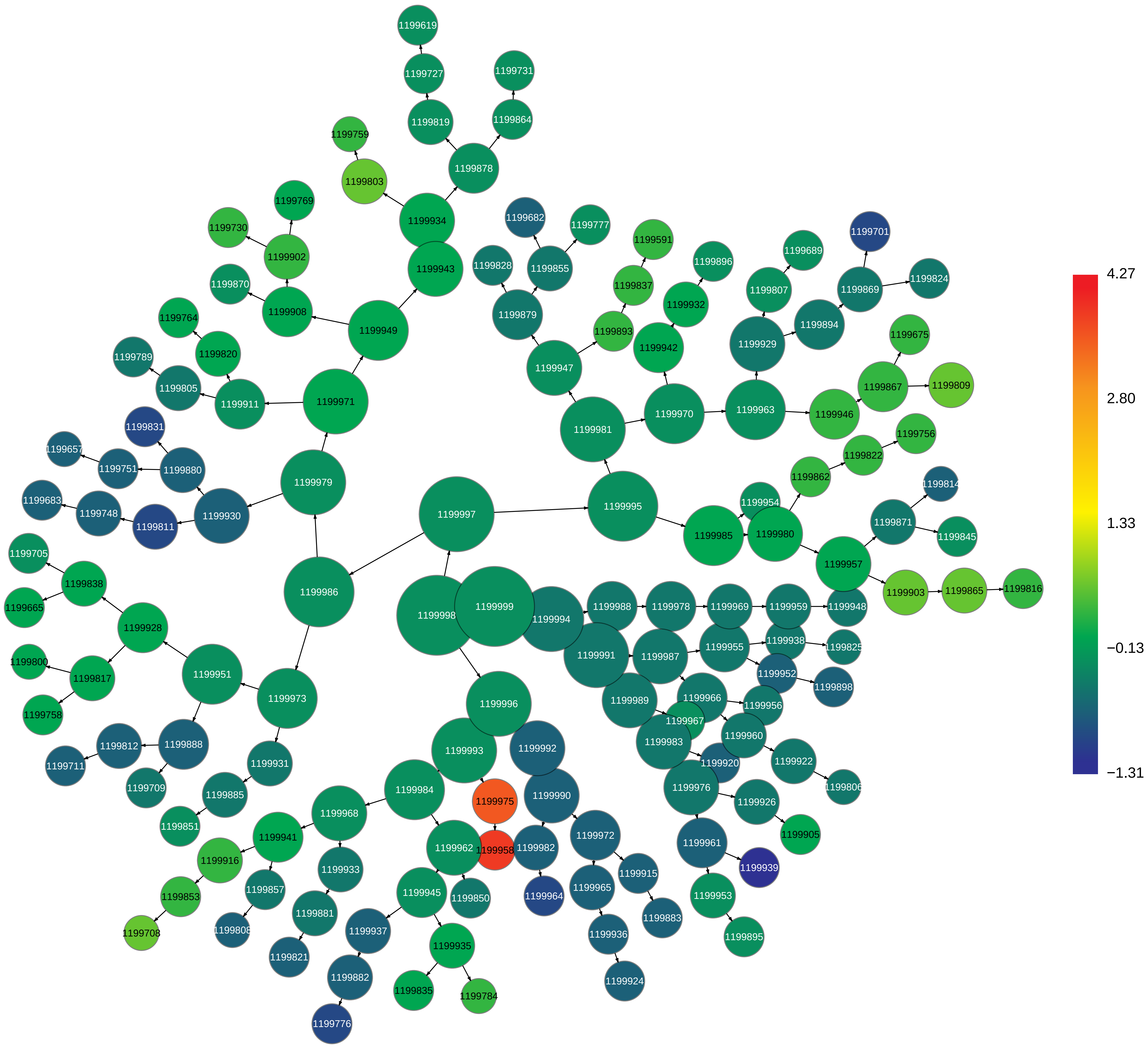

4.27
2.80
1.33
−0.13
−1.31
1199619
1199727
1199731
1199819
1199864
1199759
1199878
1199803
1199769
1199934
1199682
1199777
1199730
1199902
1199591
1199943
1199828
1199855
1199896
1199689
1199701
1199870
1199908
1199837
1199807
1199869
1199824
1199764
1199932
1199879
1199949
1199893
1199894
1199942
1199929
1199675
1199789
1199820
1199947
1199805
1199911
1199971
1199867
1199809
1199970
1199963
1199946
1199981
1199831
1199756
1199822
1199657
1199880
1199751
1199979
1199862
1199814
1199954
1199683
1199748
1199930
1199997
1199995
1199871
1199811
1199985
1199980
1199845
1199705
1199957
1199838
1199986
1199903
1199865
1199816
1199665
1199998
1199999
1199994
1199988
1199978
1199969
1199959
1199948
1199928
1199938
1199991
1199987
1199955
1199825
1199800
1199951
1199952
1199817
1199898
1199973
1199966
1199989
1199956
1199758
1199996
1199967
1199812
1199888
1199983
1199960
1199992
1199711
1199931
1199993
1199920
1199922
1199709
1199885
1199976
1199806
1199984
1199975
1199990
1199926
1199851
1199968
1199941
1199905
1199972
1199962
1199958
1199982
1199961
1199916
1199939
1199933
1199915
1199857
1199945
1199850
1199964
1199965
1199853
1199953
1199881
1199883
1199808
1199937
1199936
1199895
1199708
1199821
1199935
1199882
1199924
1199835
1199784
1199776

CD45RA

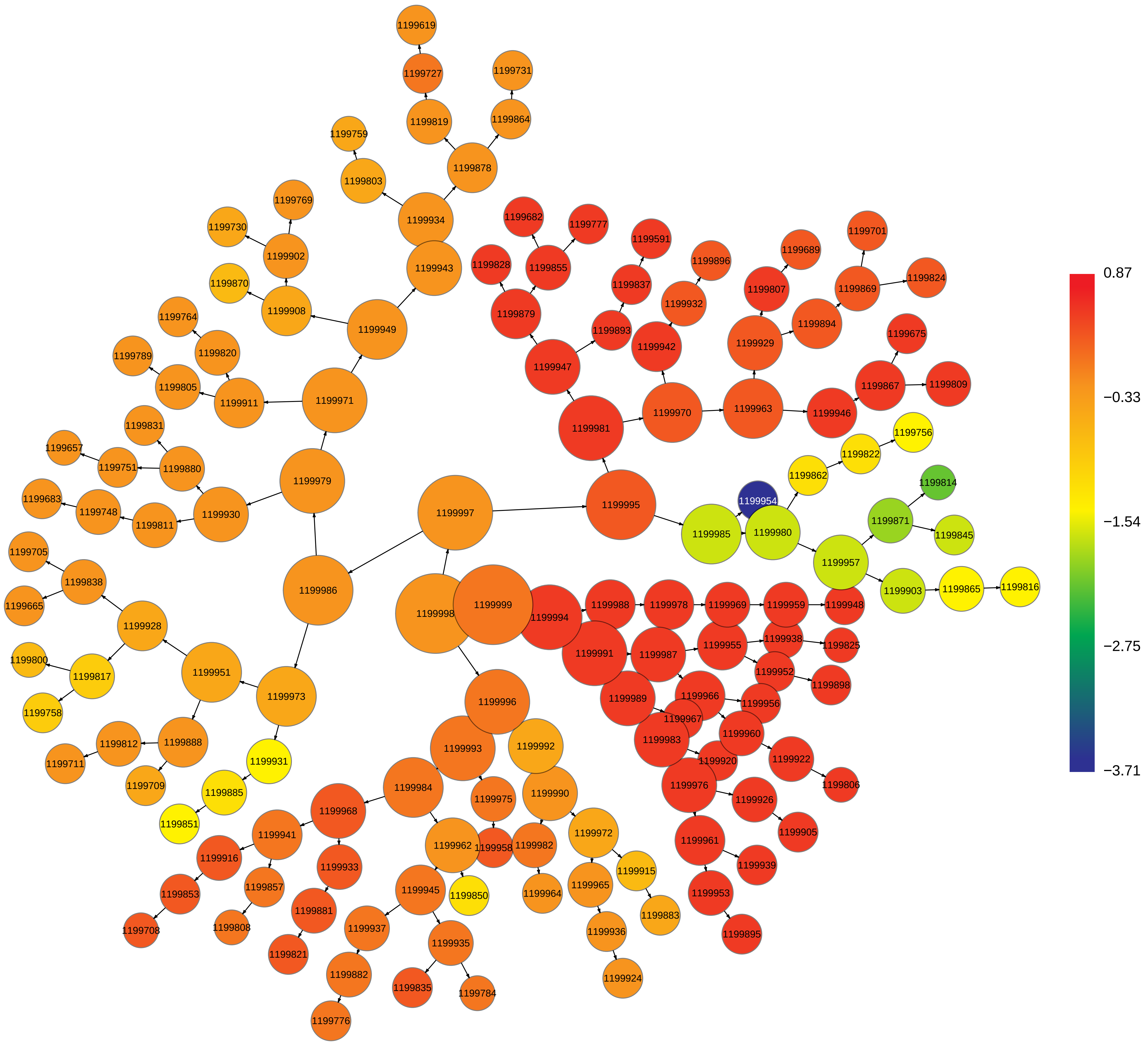
1199619
1199727
1199731
1199819
1199864
1199759
1199878
1199803
1199769
1199730
1199934
1199682
1199777
1199591
1199701
1199902
1199689
1199896
1199870
1199828
1199855
1199943
1199837
1199807
1199869
1199824
1199908
1199932
1199764
1199949
1199879
1199894
1199893
1199820
1199929
1199675
1199942
1199789
1199947
1199805
1199911
1199971
1199867
1199809
1199970
1199963
1199946
1199831
1199981
1199756
1199657
1199751
1199880
1199822
1199979
1199862
1199814
1199683
1199748
1199930
1199811
1199997
1199995
1199954
1199985
1199980
1199871
1199845
1199705
1199957
1199838
1199986
1199903
1199865
1199816
1199665
1199998
1199999
1199994
1199988
1199978
1199969
1199959
1199948
1199928
1199991
1199987
1199955
1199938
1199825
1199800
1199817
1199951
1199952
1199898
1199973
1199996
1199989
1199966
1199956
1199967
1199758
1199812
1199888
1199993
1199992
1199983
1199960
1199711
1199931
1199920
1199922
1199709
1199885
1199984
1199975
1199990
1199976
1199806
1199926
1199968
1199851
1199941
1199962
1199958
1199982
1199972
1199905
1199961
1199916
1199933
1199939
1199915
1199945
1199850
1199964
1199965
1199857
1199853
1199953
1199881
1199883
1199808
1199708
1199937
1199935
1199936
1199895
1199821
1199882
1199835
1199784
1199924
1199776
0.87
−0.33
−1.54
−2.75
−3.71

NKG2A

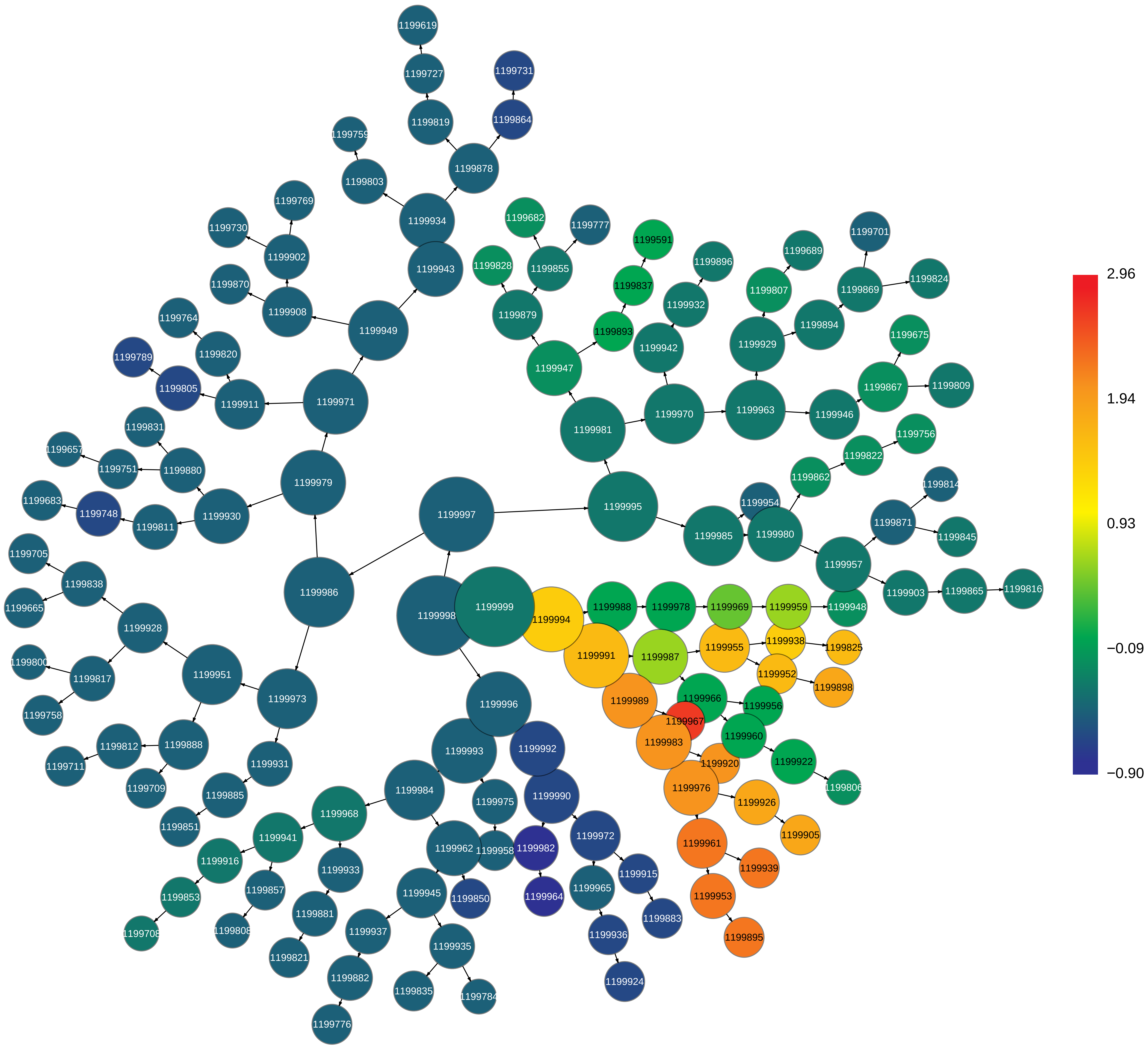

1199619
1199727
1199731
1199819
1199864
1199759
1199878
1199803
1199769
1199934
1199730
1199682
1199777
1199902
1199591
1199943
1199828
1199855
1199896
1199689
1199701
1199870
1199837
1199807
1199824
1199908
1199932
1199869
1199764
1199949
1199879
1199893
1199894
1199942
1199929
1199820
1199675
1199789
1199947
1199867
1199805
1199809
1199911
1199971
1199970
1199963
1199946
1199981
1199831
1199756
1199822
1199657
1199880
1199751
1199862
1199979
1199814
1199683
1199954
1199748
1199930
1199997
1199995
1199811
1199871
1199985
1199980
1199845
1199705
1199957
1199838
1199986
1199816
1199903
1199865
1199665
1199998
1199999
1199994
1199988
1199978
1199969
1199959
1199948
1199928
1199991
1199987
1199955
1199938
1199825
1199800
1199951
1199817
1199952
1199898
1199973
1199989
1199966
1199956
1199758
1199996
1199967
1199960
1199888
1199983
1199812
1199993
1199992
1199920
1199922
1199711
1199931
1199709
1199976
1199806
1199885
1199984
1199975
1199990
1199926
1199968
1199851
1199941
1199972
1199905
1199962
1199958
1199982
1199961
1199916
1199933
1199915
1199939
1199857
1199945
1199850
1199964
1199965
1199853
1199953
1199881
1199883
1199708
1199808
1199937
1199936
1199895
1199935
1199821
1199882
1199835
1199784
1199924
1199776
2.96
1.94
0.93
−0.09
−0.90

CD4

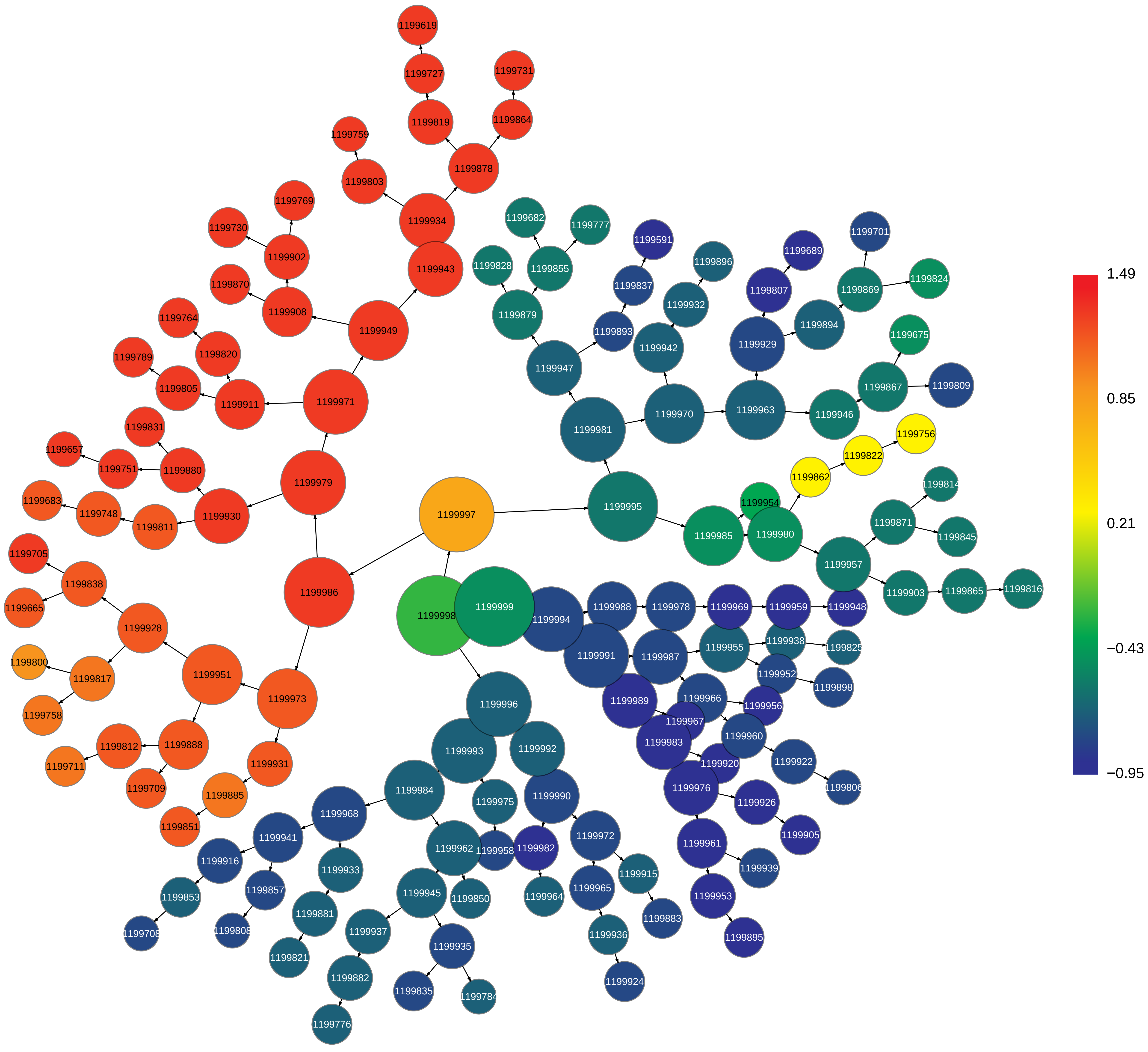

1199619
1199727
1199731
1199819
1199864
1199759
1199878
1199803
1199769
1199934
1199730
1199682
1199777
1199902
1199591
1199896
1199689
1199701
1199943
1199828
1199855
1199870
1199837
1199807
1199824
1199869
1199932
1199908
1199764
1199879
1199894
1199949
1199893
1199942
1199929
1199675
1199820
1199789
1199947
1199805
1199867
1199809
1199911
1199971
1199970
1199963
1199946
1199831
1199981
1199756
1199657
1199751
1199880
1199822
1199979
1199862
1199814
1199683
1199748
1199930
1199997
1199995
1199954
1199811
1199985
1199980
1199871
1199845
1199705
1199957
1199838
1199986
1199903
1199865
1199816
1199665
1199998
1199999
1199994
1199988
1199978
1199969
1199959
1199948
1199928
1199938
1199825
1199991
1199987
1199955
1199800
1199952
1199951
1199817
1199973
1199898
1199989
1199966
1199956
1199996
1199967
1199758
1199960
1199812
1199888
1199983
1199993
1199992
1199931
1199920
1199922
1199711
1199709
1199976
1199806
1199885
1199984
1199990
1199975
1199926
1199968
1199851
1199941
1199972
1199905
1199962
1199958
1199982
1199961
1199916
1199933
1199915
1199939
1199945
1199850
1199964
1199965
1199953
1199853
1199857
1199881
1199883
1199937
1199936
1199895
1199708
1199808
1199935
1199821
1199882
1199924
1199835
1199784
1199776
1.49
0.85
0.21
−0.43
−0.95

NKG2C

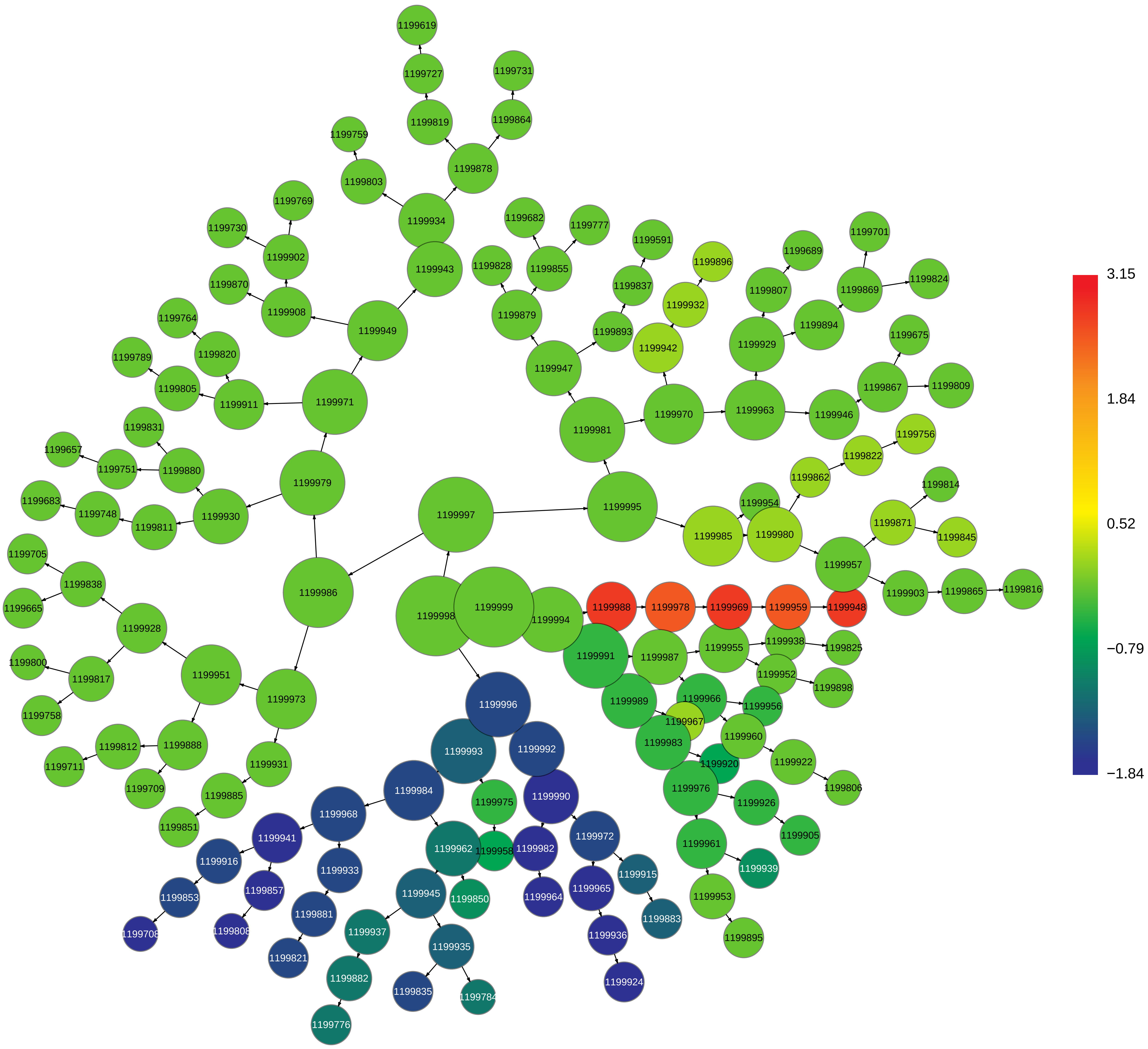
1199619
1199727
1199731
1199864
1199819
1199759
1199878
1199803
1199769
1199934
1199730
1199682
1199777
1199591
1199902
1199896
1199701
1199689
1199943
1199828
1199855
1199870
1199837
1199807
1199869
1199824
1199932
1199908
1199949
1199764
1199879
1199894
1199893
1199929
1199675
1199820
1199942
1199789
1199947
1199805
1199911
1199971
1199867
1199809
1199970
1199963
1199946
1199831
1199981
1199756
1199657
1199880
1199751
1199822
1199862
1199979
1199814
1199683
1199748
1199930
1199811
1199997
1199995
1199954
1199871
1199985
1199980
1199845
1199705
1199957
1199838
1199986
1199903
1199865
1199816
1199665
1199998
1199999
1199994
1199988
1199978
1199969
1199959
1199948
1199928
1199991
1199955
1199938
1199825
1199987
1199800
1199951
1199817
1199952
1199898
1199973
1199996
1199989
1199966
1199956
1199758
1199967
1199960
1199983
1199812
1199888
1199993
1199992
1199920
1199922
1199711
1199931
1199709
1199885
1199984
1199975
1199990
1199976
1199806
1199926
1199968
1199851
1199941
1199972
1199905
1199962
1199958
1199982
1199961
1199916
1199933
1199915
1199939
1199857
1199945
1199850
1199964
1199965
1199853
1199881
1199953
1199883
1199937
1199936
1199708
1199808
1199895
1199935
1199821
1199882
1199924
1199835
1199784
1199776
3.15
1.84
0.52
−0.79
−1.84

CD16

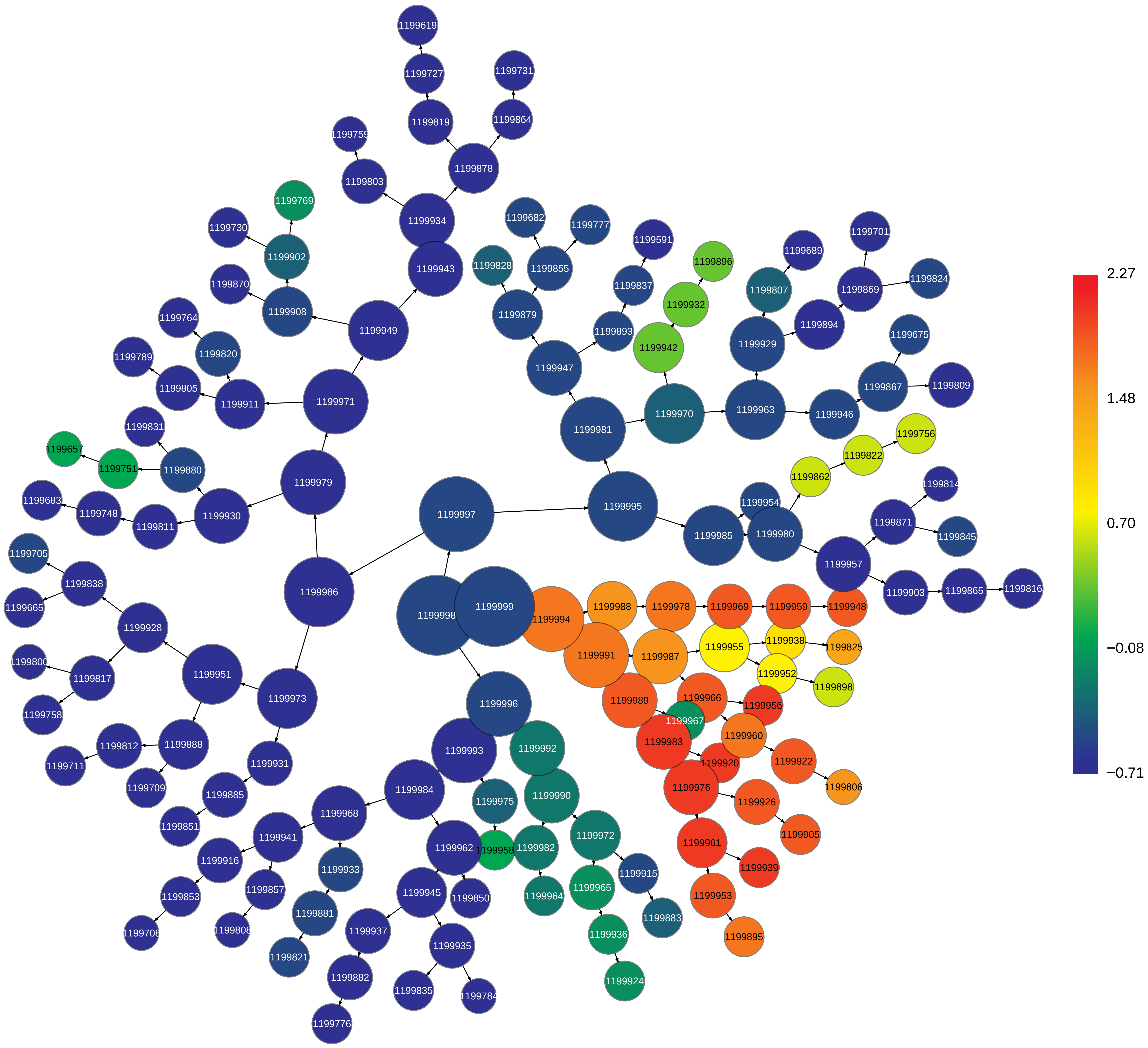
1199619
1199727
1199731
1199819
1199864
1199759
1199878
1199803
1199769
1199934
1199730
1199902
1199682
1199777
1199591
1199943
1199828
1199855
1199896
1199870
1199908
1199837
1199689
1199701
1199807
1199824
1199932
1199869
1199764
1199949
1199879
1199893
1199942
1199929
1199894
1199789
1199820
1199947
1199675
1199805
1199911
1199971
1199981
1199970
1199963
1199946
1199867
1199809
1199831
1199756
1199657
1199751
1199880
1199822
1199979
1199862
1199814
1199683
1199748
1199811
1199930
1199997
1199995
1199954
1199871
1199985
1199980
1199845
1199705
1199957
1199838
1199986
1199998
1199999
1199994
1199988
1199978
1199969
1199959
1199948
1199903
1199865
1199816
1199665
1199928
1199991
1199987
1199955
1199938
1199825
1199952
1199800
1199817
1199951
1199973
1199989
1199966
1199956
1199898
1199758
1199996
1199967
1199960
1199812
1199888
1199993
1199992
1199983
1199920
1199922
1199711
1199931
1199709
1199885
1199984
1199976
1199806
1199968
1199975
1199990
1199926
1199851
1199941
1199972
1199905
1199916
1199962
1199958
1199982
1199961
1199933
1199915
1199939
1199853
1199857
1199945
1199850
1199964
1199965
1199953
1199881
1199883
1199708
1199808
1199937
1199935
1199936
1199895
1199821
1199882
1199835
1199784
1199924
1199776
2.27
1.48
0.70
−0.08
−0.71

CD57

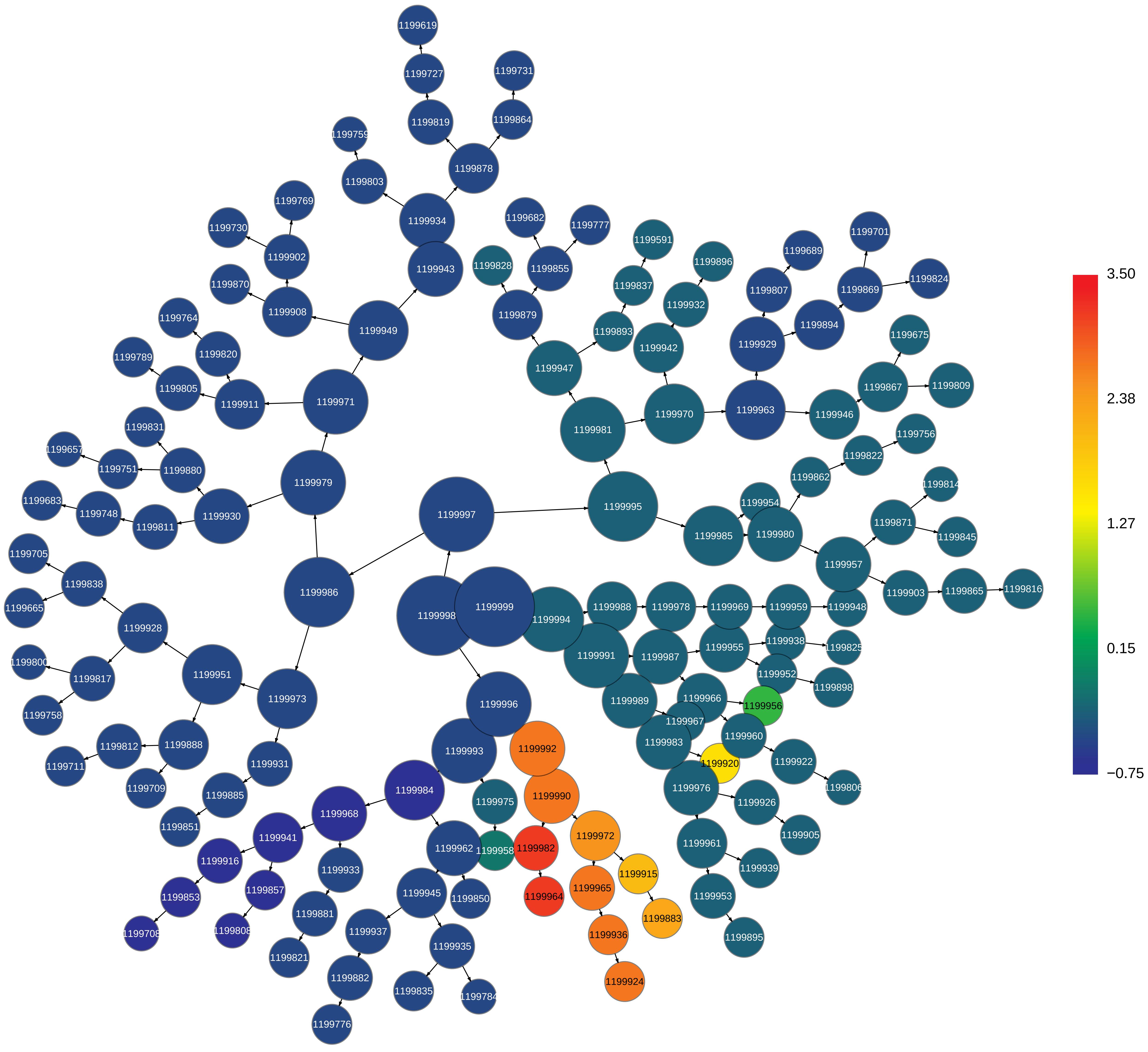

1199619
1199727
1199731
1199819
1199864
1199759
1199878
1199803
1199934
1199769
1199730
1199902
1199682
1199777
1199591
1199943
1199828
1199855
1199896
1199701
1199689
1199870
1199908
1199837
1199932
1199807
1199869
1199824
1199764
1199949
1199879
1199893
1199942
1199929
1199894
1199675
1199820
1199789
1199805
1199911
1199971
1199947
1199867
1199809
1199970
1199963
1199946
1199831
1199981
1199756
1199657
1199880
1199751
1199822
1199979
1199862
1199814
1199683
1199748
1199811
1199930
1199997
1199995
1199954
1199985
1199980
1199871
1199845
1199705
1199957
1199838
1199986
1199665
1199998
1199999
1199994
1199988
1199978
1199969
1199959
1199948
1199903
1199865
1199816
1199928
1199991
1199987
1199955
1199938
1199825
1199800
1199817
1199951
1199952
1199898
1199758
1199973
1199996
1199989
1199966
1199956
1199967
1199812
1199888
1199711
1199960
1199983
1199709
1199931
1199993
1199992
1199920
1199922
1199885
1199984
1199806
1199851
1199968
1199975
1199990
1199976
1199926
1199941
1199972
1199905
1199916
1199962
1199958
1199982
1199961
1199933
1199939
1199915
1199853
1199857
1199945
1199850
1199964
1199965
1199953
1199881
1199883
1199708
1199808
1199937
1199895
1199936
1199935
1199821
1199882
1199924
1199835
1199784
1199776
3.50
2.38
1.27
0.15
−0.75

CD3

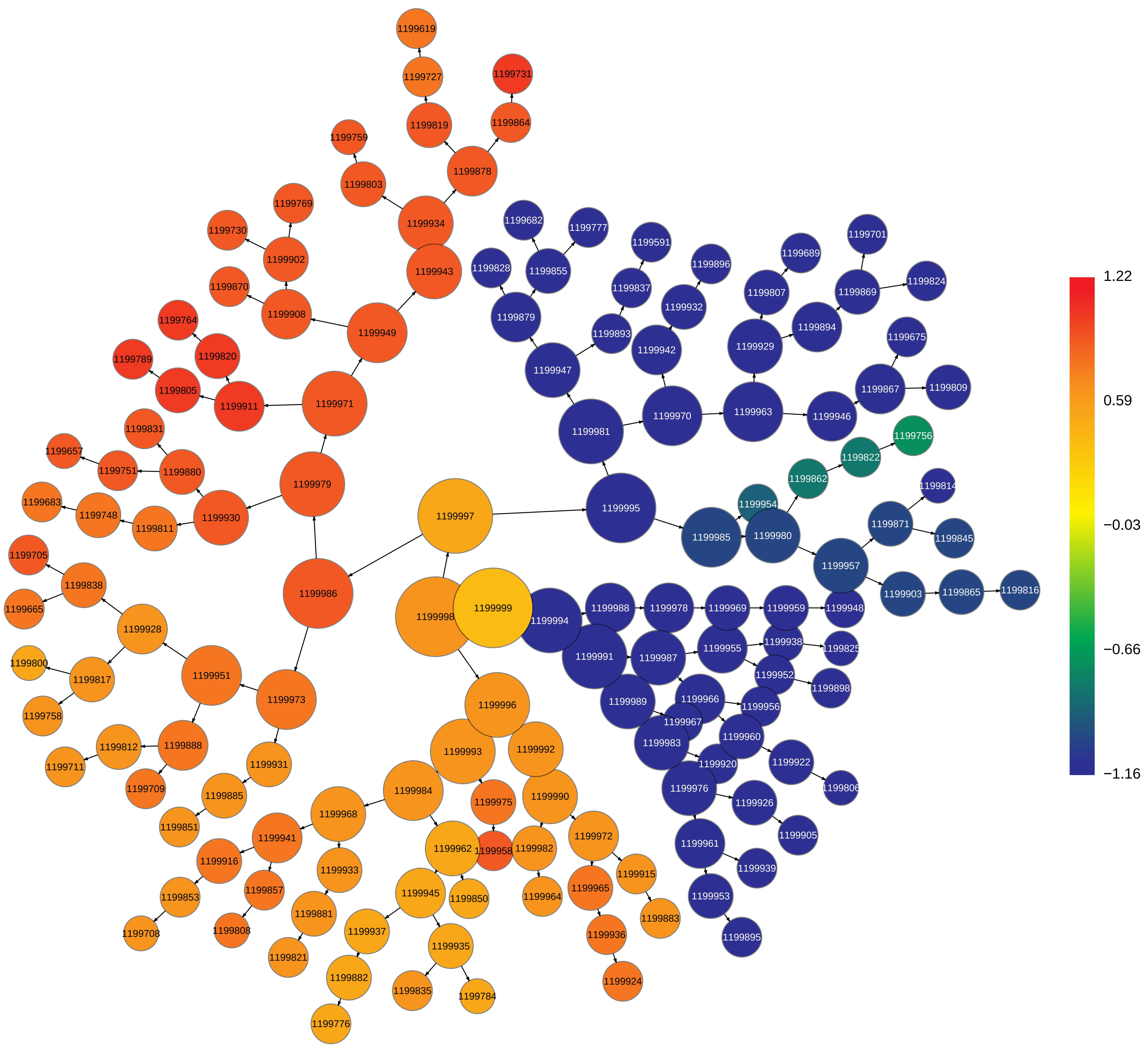

1199619
1199727
1199731
1199819
1199864
1199759
1199878
1199803
1199934
1199769
1199730
1199902
1199943
1199870
1199908
1199949
1199764
1199820
1199789
1199805
1199911
1199971
1199831
1199657
1199751
1199880
1199979
1199683
1199748
1199811
1199930
1199997
1199705
1199838
1199986
1199665
1199928
1199998
1199999
1199800
1199817
1199951
1199973
1199758
1199812
1199888
1199711
1199709
1199931
1199885
1199851
1199996
1199993
1199992
1199984
1199968
1199975
1199990
1199941
1199916
1199962
1199958
1199982
1199972
1199933
1199853
1199857
1199945
1199850
1199964
1199965
1199915
1199881
1199937
1199708
1199808
1199883
1199935
1199936
1199821
1199882
1199835
1199784
1199924
1199776
1199682
1199777
1199591
1199828
1199855
1199837
1199896
1199689
1199701
1199879
1199932
1199807
1199869
1199824
1199893
1199942
1199929
1199894
1199675
1199947
1199867
1199809
1199981
1199970
1199963
1199946
1199756
1199822
1199862
1199814
1199995
1199954
1199871
1199985
1199980
1199845
1199957
1199994
1199988
1199978
1199969
1199959
1199948
1199903
1199865
1199816
1199991
1199987
1199955
1199938
1199825
1199952
1199898
1199989
1199966
1199956
1199967
1199983
1199960
1199920
1199922
1199806
1199976
1199926
1199905
1199961
1199939
1199953
1199895
1.22
0.59
−0.03
−0.66
−1.16

CD8

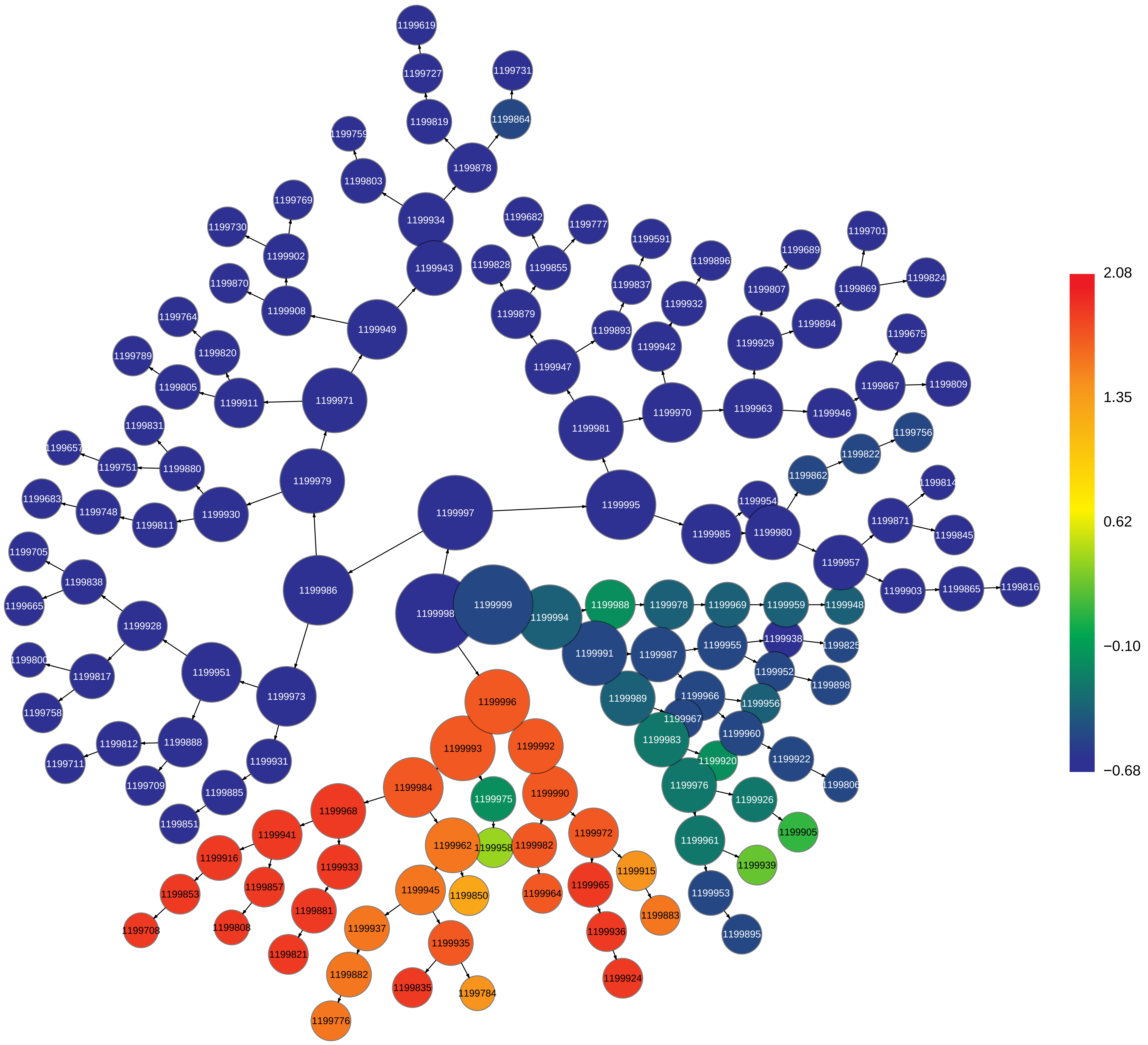
1199619
1199727
1199731
1199864
1199819
1199759
1199878
1199803
1199934
1199769
1199730
1199902
1199943
1199682
1199777
1199591
1199828
1199855
1199896
1199689
1199701
1199870
1199908
1199837
1199932
1199807
1199869
1199824
1199764
1199949
1199879
1199893
1199942
1199929
1199894
1199789
1199820
1199675
1199805
1199911
1199971
1199947
1199867
1199809
1199831
1199981
1199970
1199963
1199946
1199657
1199751
1199880
1199756
1199822
1199979
1199862
1199683
1199748
1199811
1199930
1199997
1199995
1199954
1199814
1199871
1199985
1199980
1199845
1199705
1199957
1199838
1199986
1199998
1199999
1199994
1199988
1199978
1199969
1199959
1199948
1199903
1199865
1199816
1199665
1199928
1199991
1199987
1199955
1199938
1199825
1199800
1199951
1199952
1199898
1199817
1199973
1199996
1199989
1199966
1199956
1199758
1199967
1199983
1199960
1199812
1199888
1199993
1199992
1199920
1199922
1199711
1199931
1199709
1199885
1199984
1199976
1199806
1199975
1199990
1199926
1199851
1199968
1199972
1199905
1199941
1199962
1199958
1199982
1199961
1199916
1199933
1199939
1199915
1199857
1199945
1199965
1199853
1199850
1199964
1199953
1199881
1199883
1199808
1199708
1199937
1199936
1199895
1199821
1199935
1199882
1199835
1199784
1199924
1199776
2.08
1.35
0.62
−0.10
−0.68

CCR7

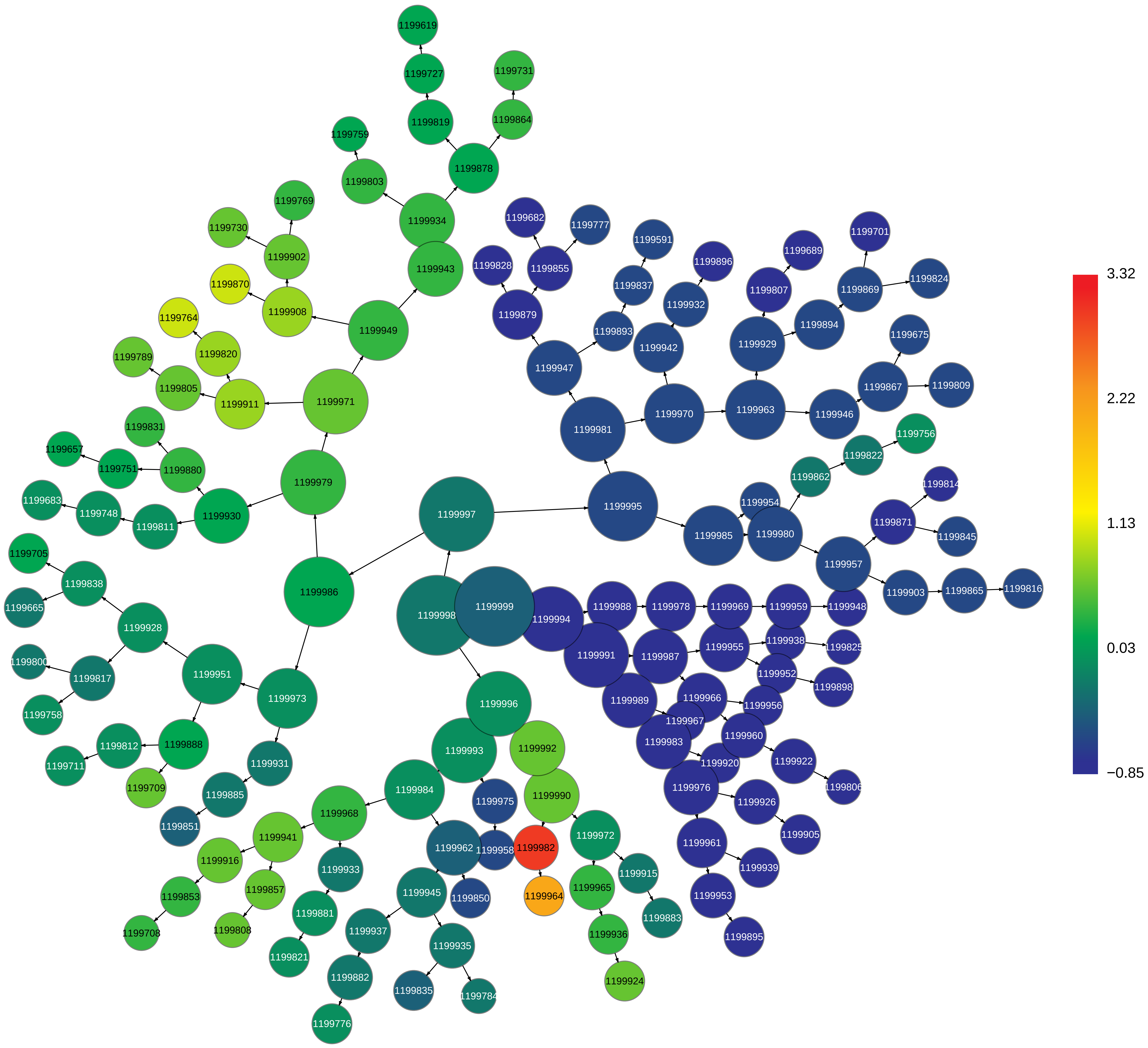
1199619
1199727
1199731
1199819
1199864
1199759
1199878
1199803
1199769
1199934
1199682
1199777
1199730
1199591
1199902
1199943
1199896
1199701
1199828
1199855
1199689
1199870
1199837
1199908
1199807
1199869
1199824
1199932
1199764
1199879
1199949
1199894
1199893
1199820
1199929
1199942
1199675
1199789
1199947
1199805
1199911
1199971
1199867
1199809
1199970
1199963
1199946
1199831
1199981
1199756
1199657
1199751
1199880
1199822
1199979
1199862
1199814
1199683
1199748
1199930
1199997
1199995
1199954
1199811
1199985
1199980
1199871
1199845
1199705
1199957
1199838
1199986
1199816
1199865
1199903
1199948
1199665
1199999
1199998
1199994
1199988
1199978
1199969
1199959
1199928
1199938
1199825
1199991
1199987
1199955
1199800
1199952
1199817
1199951
1199898
1199973
1199989
1199966
1199956
1199996
1199758
1199967
1199960
1199983
1199812
1199888
1199992
1199993
1199711
1199931
1199920
1199922
1199709
1199885
1199984
1199976
1199806
1199990
1199975
1199926
1199968
1199851
1199941
1199972
1199962
1199982
1199958
1199961
1199905
1199916
1199933
1199915
1199939
1199945
1199857
1199850
1199964
1199965
1199853
1199953
1199881
1199883
1199937
1199895
1199708
1199808
1199936
1199935
1199821
1199882
1199924
1199835
1199784
1199776
3.32
2.22
1.13
0.03
−0.85

CD56

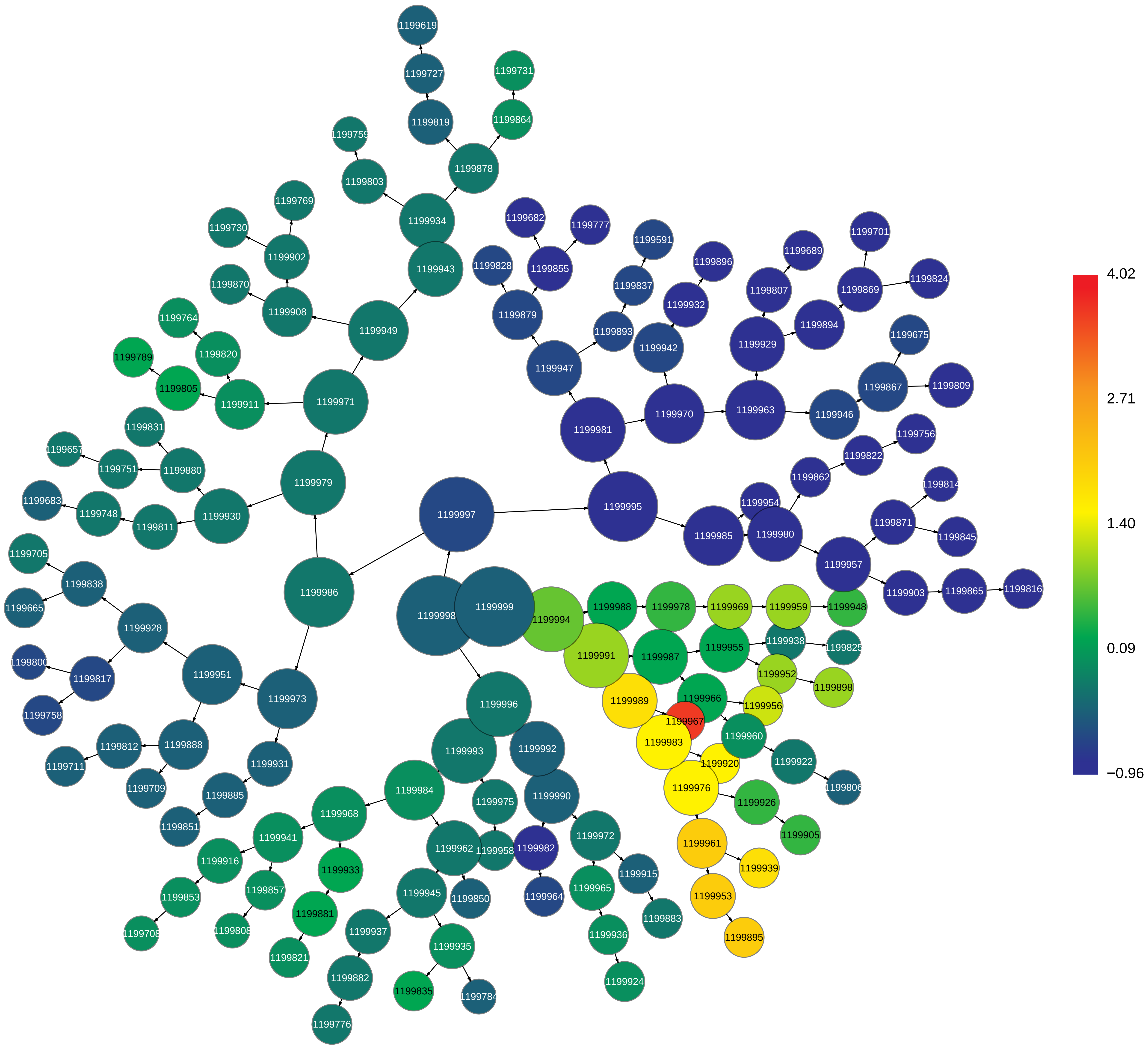

1199619
1199727
1199731
1199819
1199864
1199759
1199878
1199803
1199769
1199934
1199730
1199902
1199682
1199777
1199591
1199943
1199828
1199855
1199896
1199689
1199701
1199870
1199837
1199908
1199807
1199869
1199824
1199932
1199764
1199879
1199949
1199893
1199894
1199942
1199929
1199675
1199820
1199789
1199947
1199805
1199911
1199971
1199867
1199809
1199970
1199963
1199946
1199981
1199831
1199756
1199657
1199822
1199751
1199880
1199979
1199862
1199814
1199683
1199748
1199954
1199930
1199811
1199997
1199995
1199871
1199985
1199980
1199845
1199705
1199957
1199838
1199986
1199816
1199665
1199998
1199999
1199994
1199988
1199978
1199969
1199959
1199948
1199903
1199865
1199928
1199938
1199825
1199991
1199987
1199955
1199800
1199817
1199951
1199952
1199898
1199973
1199989
1199966
1199956
1199996
1199967
1199758
1199983
1199960
1199812
1199888
1199993
1199992
1199920
1199922
1199711
1199931
1199709
1199885
1199984
1199975
1199990
1199976
1199806
1199926
1199851
1199968
1199941
1199962
1199958
1199982
1199972
1199905
1199961
1199916
1199933
1199939
1199915
1199853
1199857
1199945
1199850
1199964
1199965
1199953
1199881
1199883
1199708
1199808
1199937
1199936
1199895
1199821
1199935
1199882
1199835
1199784
1199924
1199776
4.02
2.71
1.40
0.09
−0.96

PD−1

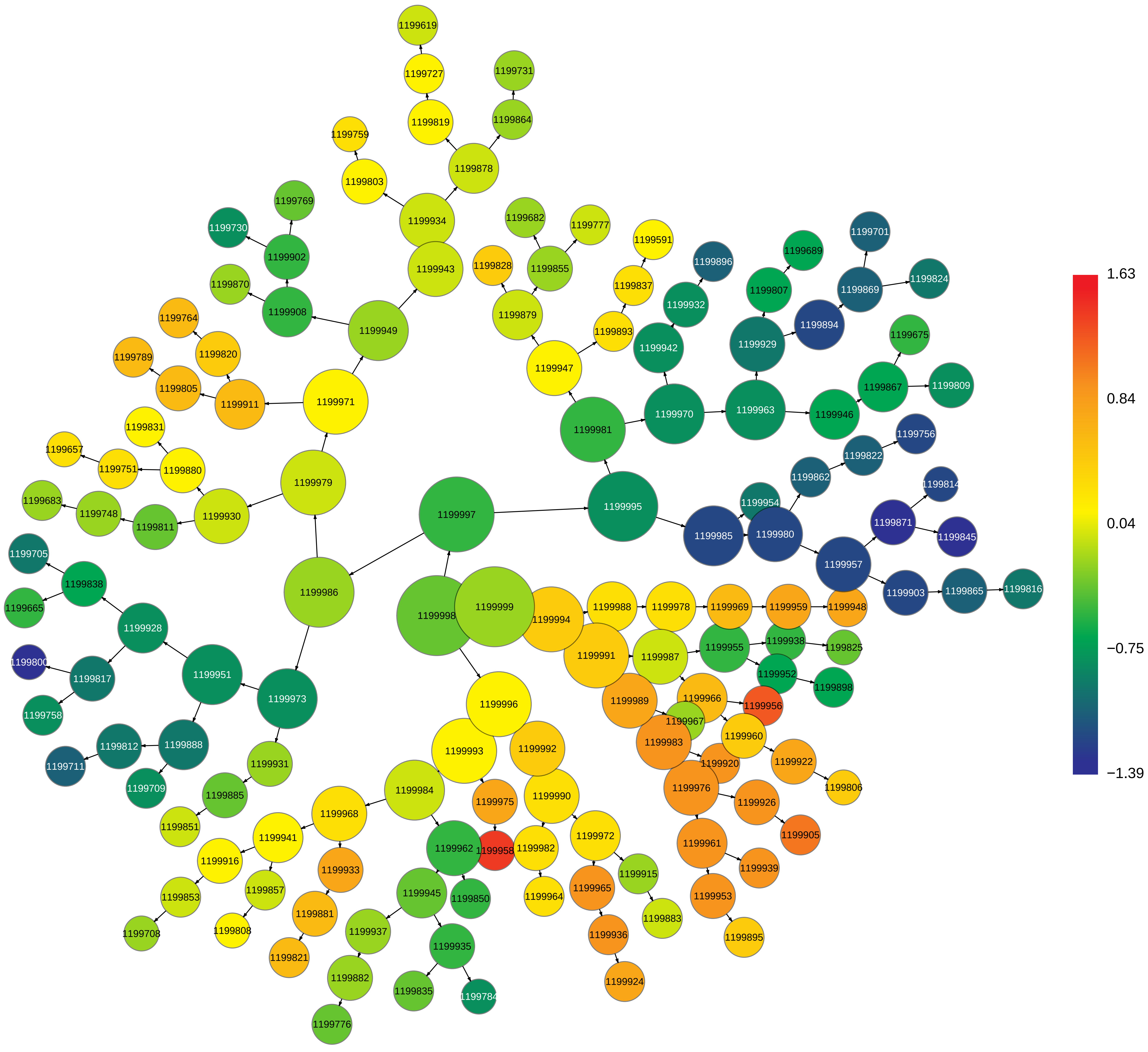
1199619
1199727
1199731
1199819
1199864
1199759
1199878
1199803
1199769
1199934
1199730
1199682
1199777
1199902
1199591
1199896
1199689
1199701
1199828
1199943
1199855
1199837
1199870
1199807
1199824
1199932
1199869
1199908
1199764
1199949
1199879
1199894
1199893
1199929
1199942
1199675
1199820
1199789
1199947
1199867
1199809
1199805
1199911
1199971
1199970
1199963
1199946
1199981
1199831
1199756
1199822
1199657
1199880
1199751
1199979
1199862
1199814
1199683
1199954
1199748
1199930
1199997
1199995
1199811
1199871
1199985
1199980
1199845
1199705
1199957
1199838
1199986
1199903
1199865
1199816
1199665
1199998
1199999
1199994
1199988
1199978
1199969
1199959
1199948
1199928
1199938
1199991
1199987
1199955
1199825
1199800
1199817
1199951
1199952
1199898
1199973
1199989
1199966
1199996
1199956
1199967
1199758
1199888
1199812
1199983
1199960
1199993
1199992
1199920
1199931
1199922
1199711
1199709
1199885
1199976
1199806
1199984
1199990
1199926
1199975
1199968
1199851
1199905
1199941
1199962
1199958
1199982
1199972
1199961
1199916
1199933
1199939
1199857
1199915
1199965
1199853
1199945
1199850
1199964
1199953
1199881
1199883
1199708
1199808
1199937
1199936
1199895
1199935
1199821
1199882
1199924
1199835
1199784
1199776
1.63
0.84
0.04
−0.75
−1.39

NK_T_CITRUS_2clusters

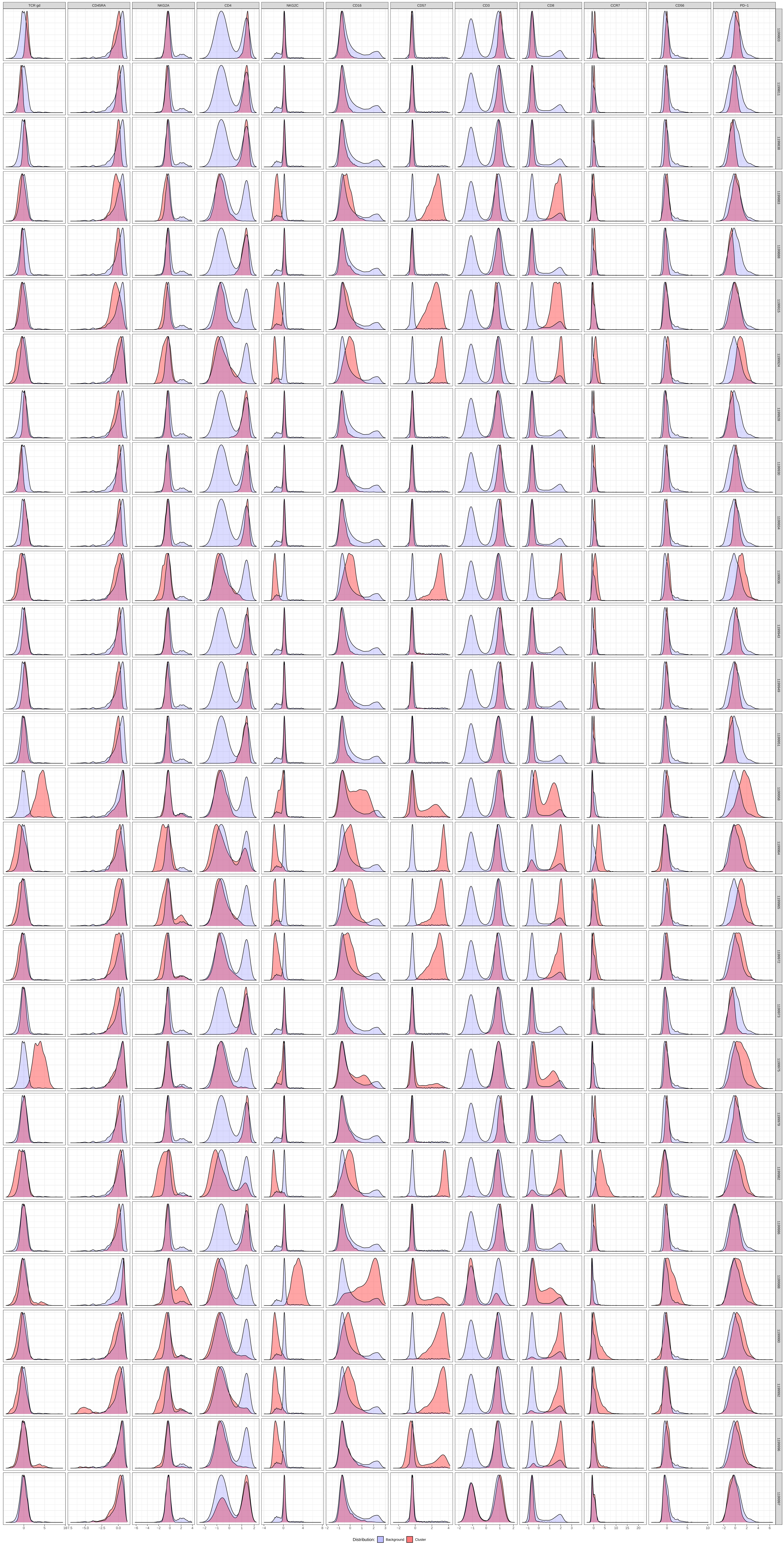
TCR gd
CD45RA
NKG2A
CD4
NKG2C
CD16
CD57
CD3
CD8
CCR7
CD56
PD−1
1199803
1199811
1199838
1199883
1199888
1199915
1199924
1199928
1199930
1199934
1199936
1199943
1199949
1199951
1199958
1199964
1199965
1199972
1199973
1199975
1199979
1199982
1199986
1199988
1199990
1199992
1199996
1199997
Distribution:
Background
Cluster

NK_T_CITRUS_3plots

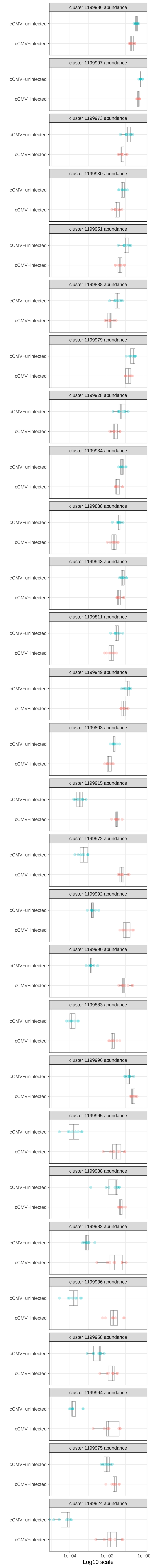
cluster 1199986 abundance
cluster 1199997 abundance
cluster 1199973 abundance
cluster 1199930 abundance
cluster 1199951 abundance
cluster 1199838 abundance
cluster 1199979 abundance
cluster 1199928 abundance
cluster 1199934 abundance
cluster 1199888 abundance
cluster 1199943 abundance
cluster 1199811 abundance
cluster 1199949 abundance
cluster 1199803 abundance
cluster 1199915 abundance
cluster 1199972 abundance
cluster 1199992 abundance
cluster 1199990 abundance
cluster 1199883 abundance
cluster 1199996 abundance
cluster 1199965 abundance
cluster 1199988 abundance
cluster 1199982 abundance
cluster 1199936 abundance
cluster 1199958 abundance
cluster 1199964 abundance
cluster 1199975 abundance
cluster 1199924 abundance
cCMV−uninfected
cCMV−infected
1e−04
1e−02
1e+00
Log10 scale
